# Supplementary material for: Structure-based development and preclinical evaluation of the SARS-CoV-2 3C-like protease inhibitor simnotrelvir
Source: Nat Commun. 2023 Oct 13;14:6463. doi: 10.1038/s41467-023-42102-y (PMC10575921; doi:10.1038/s41467-023-42102-y)
Supplement: Supplementary file 1 — Supplementary Information [file 41467_2023_42102_MOESM1_ESM.pdf]

Structure-based development and preclinical evaluation of the SARS-CoV-2 3C-like protease inhibitor simnotrelvir

Xiangrui Jiang[1,2,11], Haixia Su[1,11], Weijuan Shang[3,11], Feng Zhou[4,5,11], Yan Zhang[1,11], Wenfeng Zhao[1,11], Qiumeng Zhang[1], Hang Xie[1,6], Lei Jiang[5], Tianqing Nie[1,6], Feipu Yang[1], Muya Xiong[1,2], Xiaoxing Huang[5], Minjun Li[7], Ping Chen[8], Shaoping Peng[4,8], Gengfu Xiao[3], Hualiang Jiang[1,12], Renhong Tang[4,5]*, Leike Zhang[3,9]*, Jingshan Shen[1,2]*, Yechun Xu[1,2,10]*

[1]State Key Laboratory of Drug Research, Shanghai Institute of Materia Medica, Chinese Academy of Sciences, Shanghai 201203, China

[2]University of Chinese Academy of Sciences, Beijing 100049, China

[3]State Key Laboratory of Virology, Wuhan Institute of Virology, Center for Biosafety Mega-Science, Chinese Academy of Sciences, Wuhan 430071, China

[4]State Key Laboratory of Neurology and Oncology Drug Development, Nanjing 210023, China

[5]Simcere Zaiming Pharmaceutical Co., Ltd., Shanghai 200000, China

[6]School of Chinese Materia Medica, Nanjing University of Chinese Medicine, Nanjing 210023, China

[7]Shanghai Synchrotron Radiation Facility, Shanghai Advanced Research Institute, Chinese Academy of Sciences, Shanghai 201210, China

[8]Jiangsu Simcere Pharmaceutical Co., Ltd, Nanjing 210023, China

[9]Hubei jiangxia Laboratory, Wuhan 430200, China

[10]School of Pharmaceutical Science and Technology, Hangzhou Institute for Advanced Study, University of Chinese Academy of Sciences, Hangzhou 310024, China

[11]These authors contributed equally

[12]Deceased

*Correspondence: renhong.tang@simceregroup.com; zhangleike@wh.iov.cn; shenjingshan@simm.ac.cn; ycxu@simm.ac.cn

Table of Contents

	Initial page
**Supplementary Figures**	**3**
**Supplementary Tables**	**22**
**Spectral Data for Synthetic Compounds**	**29**

S1: Supplementary Figures

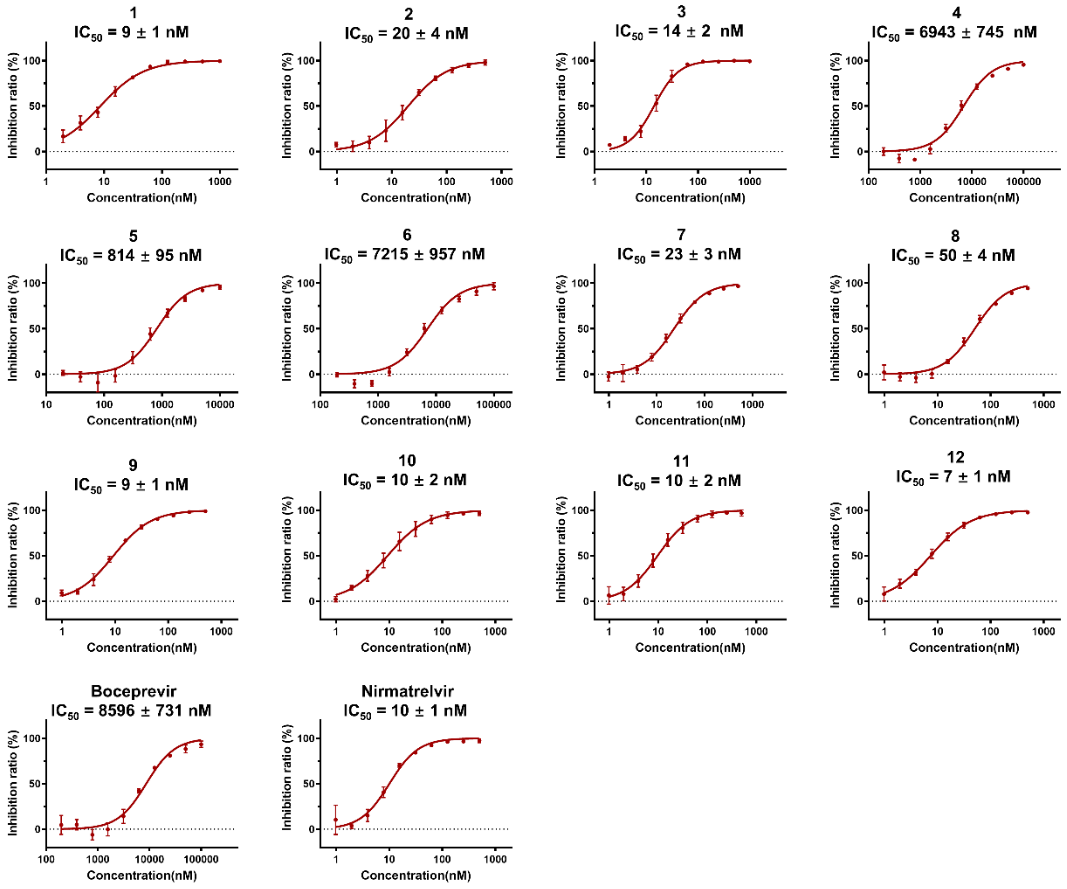

Supplementary Fig. 1 | Concentration-dependent inhibition of SARS-CoV-2 3CL^{pro} by compounds. Inhibition curves for compounds **1-12**, boceprevir and nirmatrelvir against SARS-CoV-2 3CL^{pro} resulted from the FRET-based enzymatic assays. Error bars represent mean ± SD of three independent experiments. Source data are provided as a Source Data file.

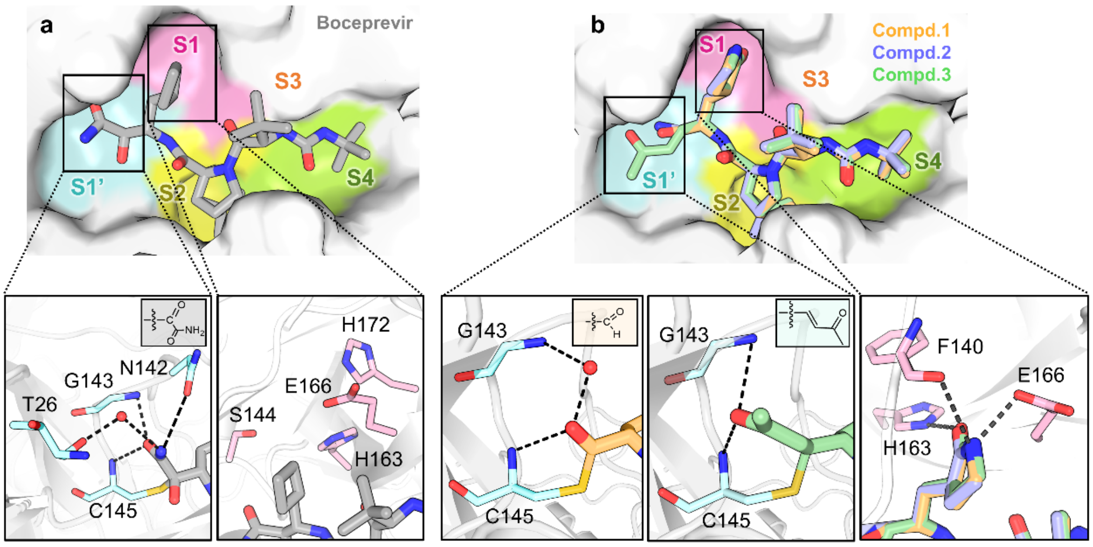

Supplementary Fig. 2 | Binding modes of boceprevir (a) and compounds 1-3 (b) with SARS-CoV-2 3CL^{pro} revealed by X-ray crystal structures. Molecular surface representations of SARS-CoV-2 3CL^{pro} in complex with boceprevir (PDB code: 6XQU), compounds **1** (PDB code: 8IFP), **2** (PDB code: 8IFQ) and **3** (PDB code: 8IFR). The S1'-S4 subsites are colored in cyan, magenta, yellow, orange, and green, respectively. Boceprevir and compounds **1-3** are shown as gray, orange, purple, and green sticks, respectively. H-bonds are represented by black dashed lines.

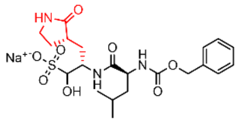

GC376

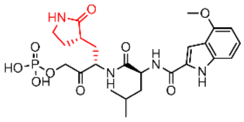

Lufotrelvir
(PF-07304814)

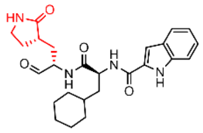

Bofutrelvir
(FB2001)

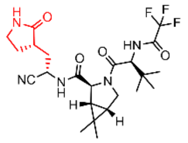

Nirmatrelvir

Supplementary Fig. 3 | Chemical structures of GC376, lufotrelvir, bofutrelvir, and nirmatrelvir.

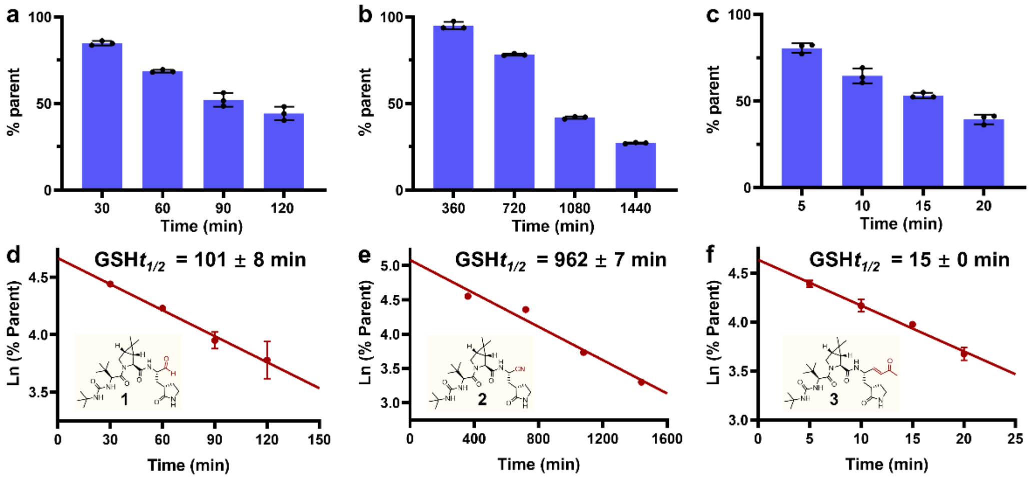

Supplementary Fig. 4 | Half-life time determination of compounds 1-3 reacting with GSH. a, b, c, The remaining GSH after incubation with compounds **1** (**a**), **2** (**b**) and **3** (**c**) for indicated time. **d, e, f,** Ln (the percentage of the remaining GSH) are plotted against incubation time to generate the half-life time of compounds **1** (**d**), **2** (**e**), and **3** (**f**) reacting with GSH. Error bars represent mean ± SD of three independent experiments. Source data are provided as a Source Data file.

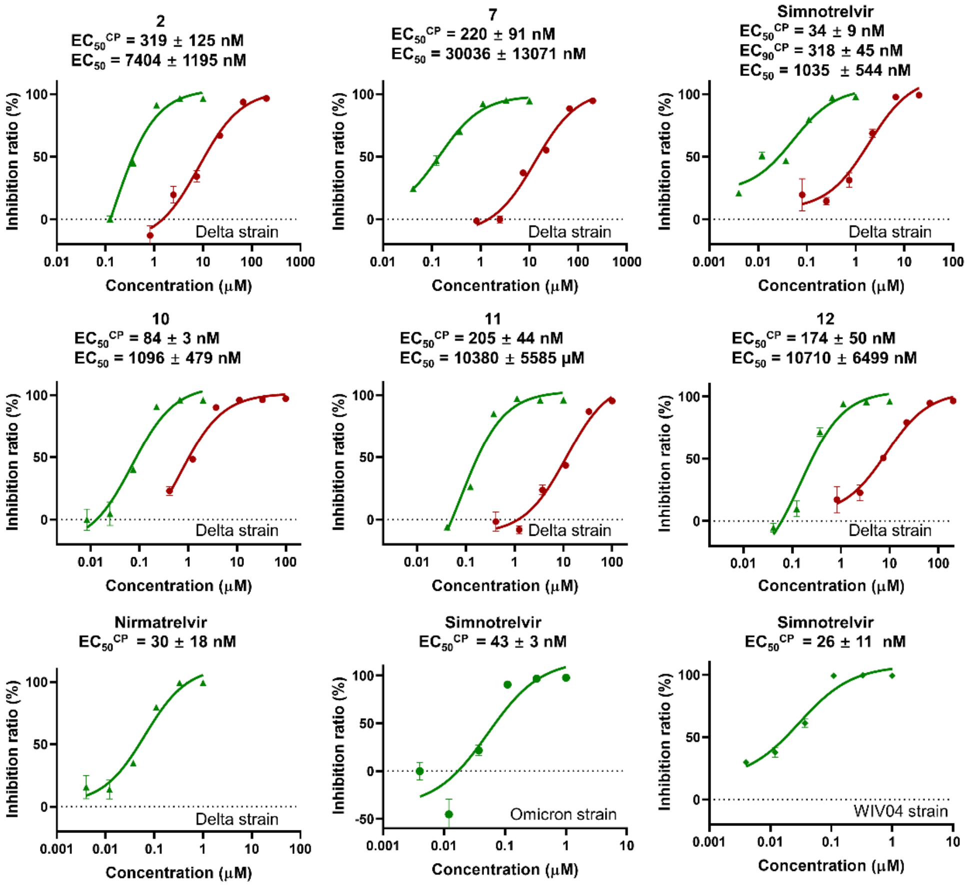

Supplementary Fig. 5 | *In vitro* cellular antiviral activity of compounds. Representative inhibition curves for compounds **2**, **7**, **10-12**, simnotrelvir, and nirmatrelvir against SARS-CoV-2 WIV04, Delta and Omicron strains in Vero E6 cells. The EC_{50}^{CP} means the EC_{50} value measured in the presence of 0.5 μM P-glycoprotein efflux inhibitor CP-100356. Error bars represent mean ± SD. At least three independent experiments were performed. Source data are provided as a Source Data file.

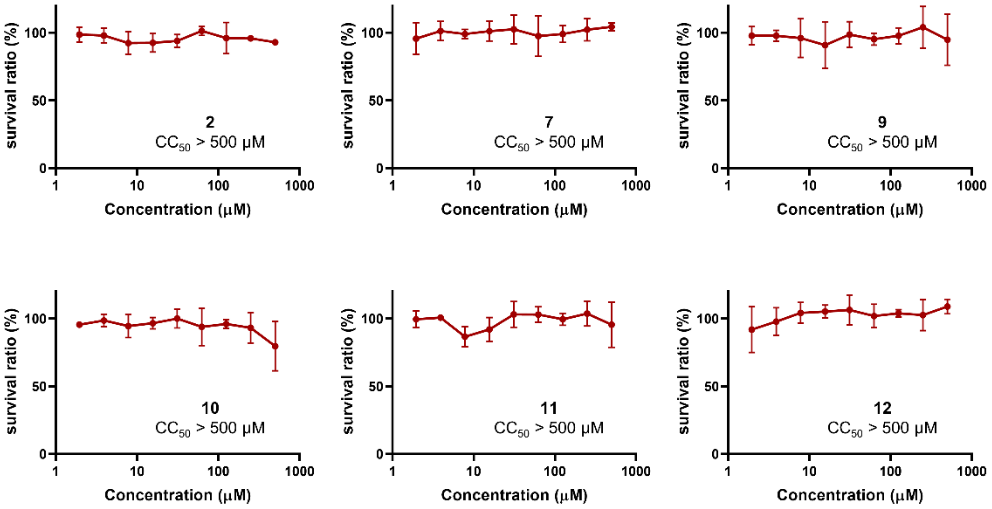

Supplementary Fig. 6 | Cytotoxicities of compounds 2, 7, and 9-12 in Vero E6 cells. Error bars represent mean ± SD of three independent experiments. Source data are provided as a Source Data file.

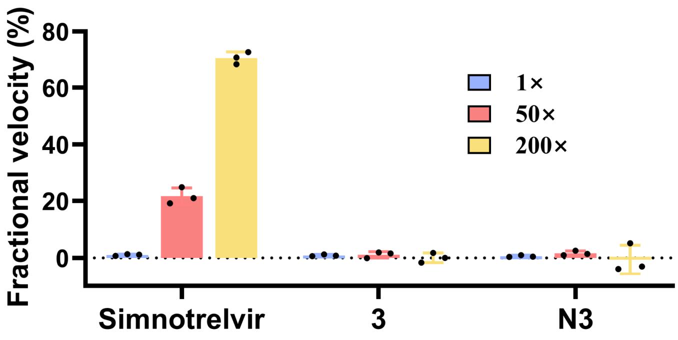

Supplementary Fig. 7 | Reversibility evaluation of SARS-CoV-2 3CL^{pro}-inhibitor complexes. The ratios of fractional velocity after 1-fold, 50-fold and 200-fold dilution are colored in blue, salmon and yellow, respectively. Error bars represent mean ± SD of three independent experiments. Source data are provided as a Source Data file.

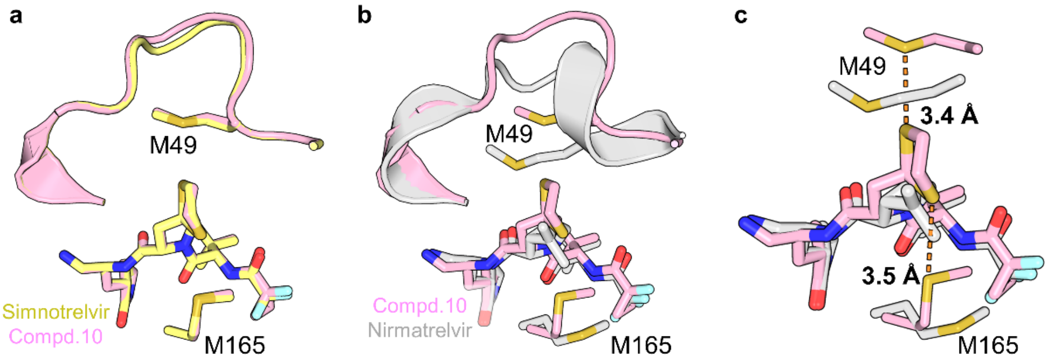

Supplementary Fig. 8 | Comparison of binding modes of compounds 10, simnotrelvir and nirmatrelvir with SARS-CoV-2 3CL^{pro}. a, The overlap of binding poses of simnotrelvir (yellow sticks) and compound **10** (pink sticks) by superimposing crystal structures of SARS-CoV-2 3CL^{pro} in complex with two inhibitors. **b, c,** The overlap of binding poses of compound **10** (pink sticks) and nirmatrelvir (white sticks). Residues 40-51 of SARS-CoV-2 3CL^{pro} are shown as cartoons. The distances are represented by orange dashed lines.

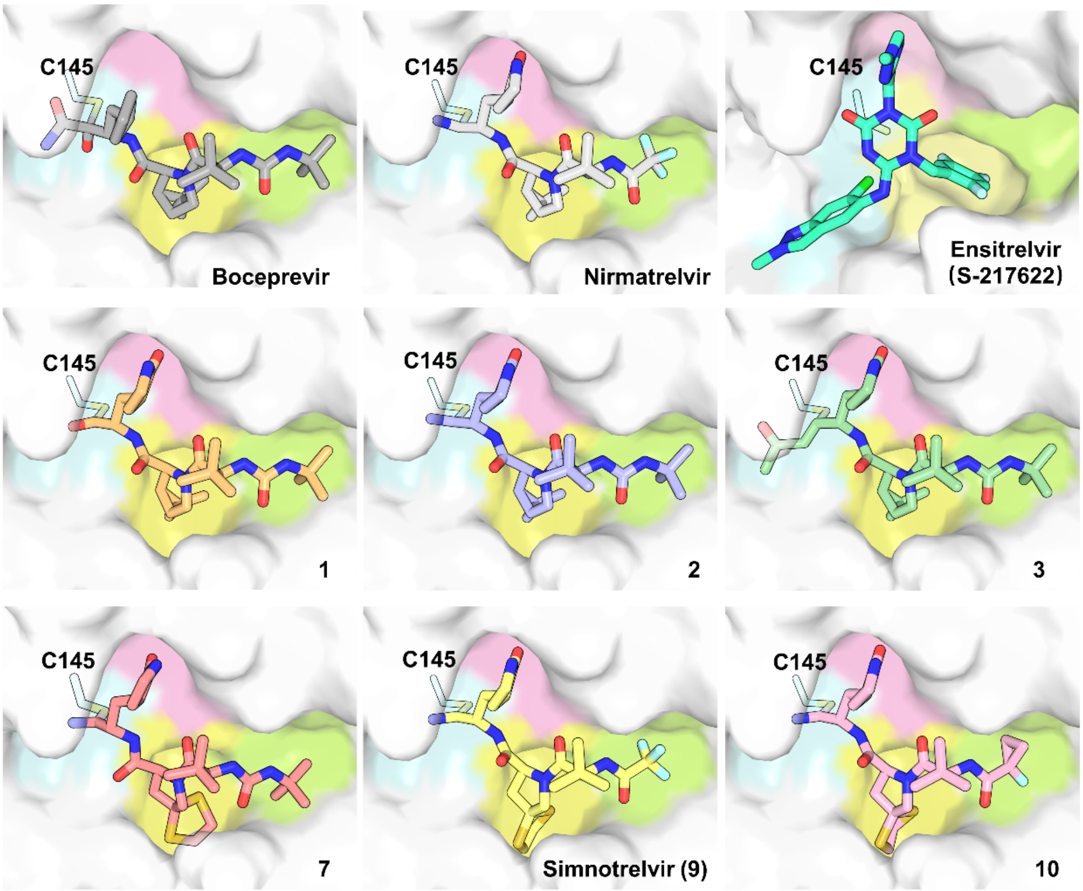

Supplementary Fig. 9 | Molecular surface representations of SARS-CoV-2 3CL^{pro} in complex with boceprevir, nirmatrelvir, ensitrelvir, simnotrelvir, and compounds 1, 2, 3, 7, and 10 in the co-crystal structures. The compounds are shown as sticks with different colors.

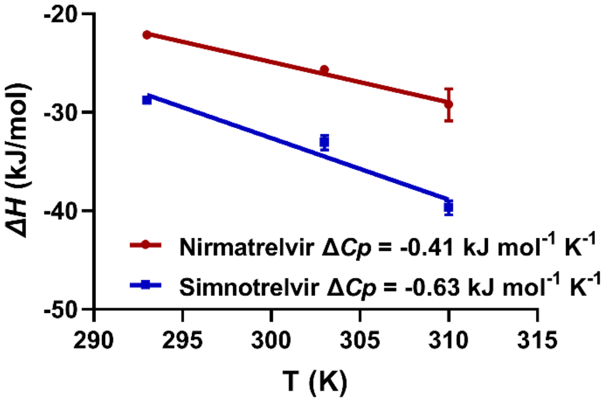

Supplementary Fig. 10 | Temperature dependence of the measured enthalpy (Δ*H*) of SARS-CoV-2 3CL^{pro} binding to nirmatrelvir (red) and simnotrelvir (blue). Three independent experiments were performed at each temperature. Source data are provided as a Source Data file.

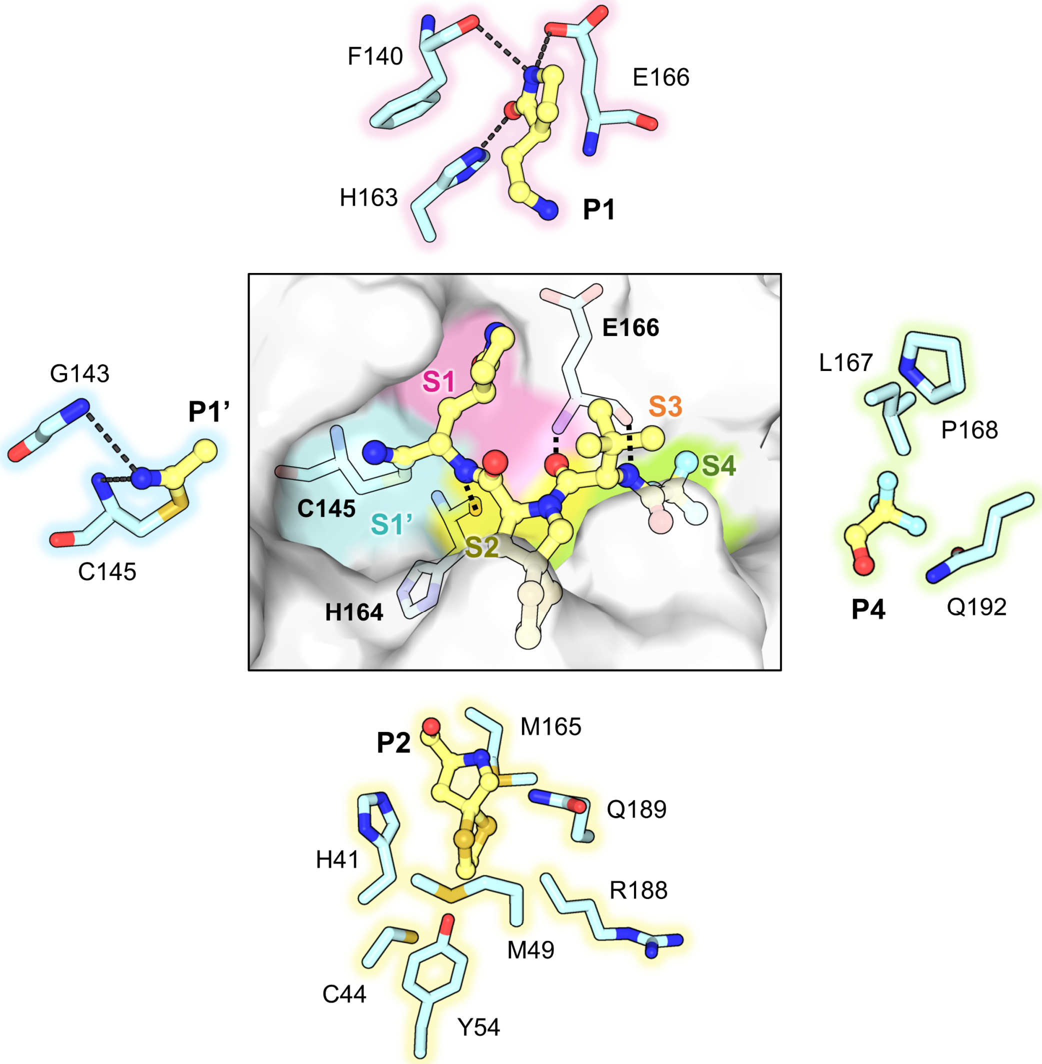

Supplementary Fig. 11 | Detailed interactions of simnotrelvir with SARS-CoV-2 3CL^{pro} revealed by the co-crystal structure. The protease is represented by molecular surface. The S1’-S4 subsites are colored in cyan, magenta, yellow, orange, and green, respectively. Simnotrelvir is represented as yellow ball and sticks. The residues interacting with simnotrelvir are shown as cyan sticks. H-bonds are displayed by black dashed lines.

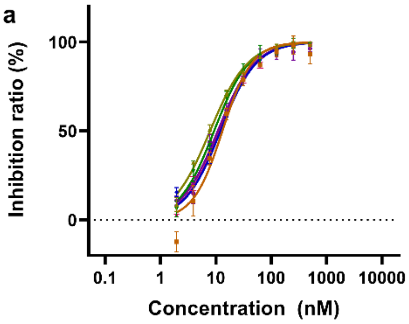

Mutations	Variants	IC_{50} (nM)
WT	i.a. B.1.1.7 (Alpha), B.1.617.2 (Delta)	9 ± 1
G15S	C.37 (Lambda)	13
T21I	B.1.1.318	8
L89F	B.1.2	9
K90R	B.1.351 (Beta)	12
P132H	B.1.1.529, BA.1, BA.2, BA.4, BA.5, XBB (Omicron)	11
L205V	P.2 (Zeta)	12

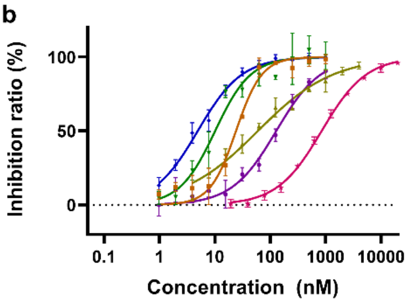

Proteases	IC_{50} (nM)
SARS-CoV 3CL^{pro}	24
MERS-CoV 3CL^{pro}	60
HKU1-CoV 3CL^{pro}	10
OC43-CoV 3CL^{pro}	5
H229E-CoV 3CL^{pro}	129
NL63-CoV 3CL^{pro}	849

Supplementary Fig. 12 | Concentration-dependent inhibition of 3CL^{pro} of six SARS-CoV-2 variants (a) and six human coronaviruses (b) by simnotrelvir resulted from the FRET-based enzymatic assays. Error bars were graphed as mean ± SD of triplicate. Source data are provided as a Source Data file.

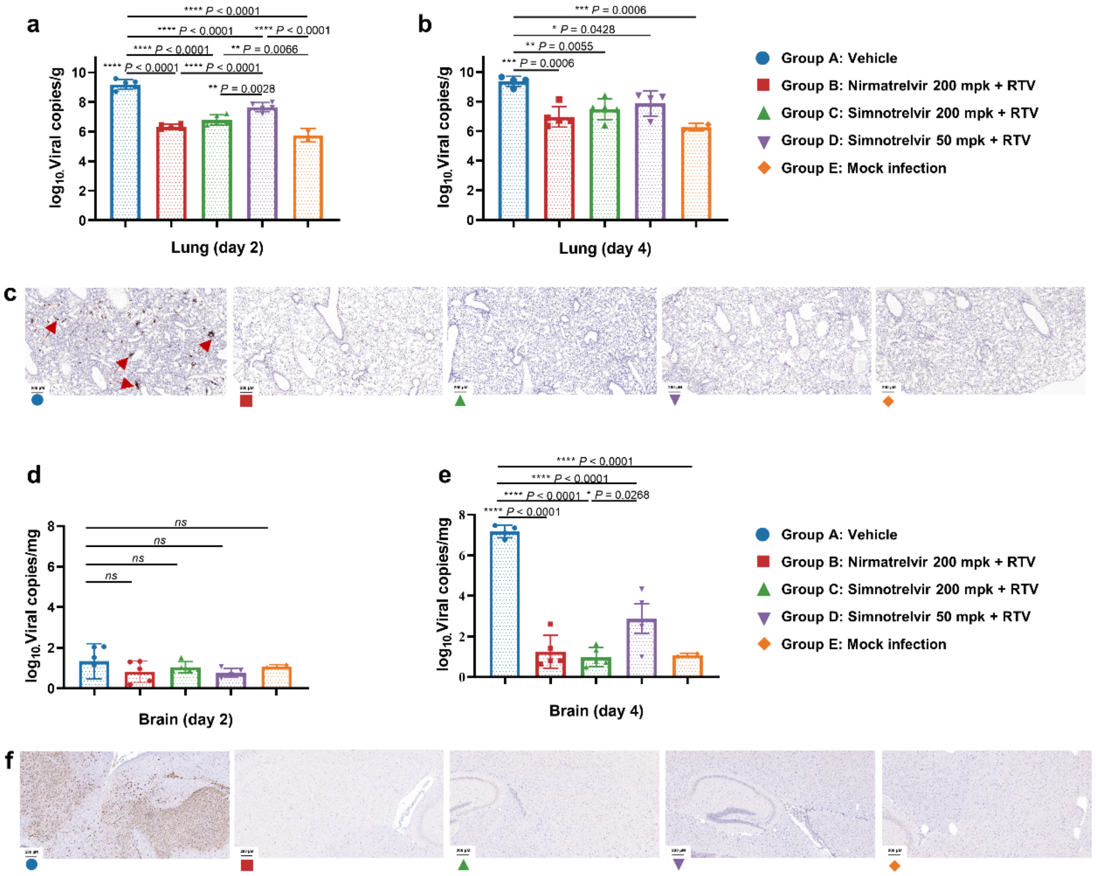

Supplementary Fig. 13 | *In vivo* antiviral activity of simnotrelvir in K18-hACE2 mice infected by SARS-CoV-2 Delta. a, The virus copy number in lungs at Day 2 post infection (n = 5, 5, 5, 5, and 2 for group A-E). **b,** The virus copy number in lungs at Day 4 post infection (n = 4, 5, 5, 4, and 2 for group A-E). **c,** Immunohistochemistry (IHC) analysis in lungs at Day 2 post infection by using an anti–SARS-CoV-2 nucleocapsid protein antibody **a,** The virus copy number in lungs at Day 2 post infection (n = 5, 5, 5, 5, and 2 for group A-E). Red arrows represent the stained virus nucleocapsid protein. **d,** Virus copy number in mice brains at Day 2 post infection (n = 5, 5, 5, 5, and 2 for group A-E). **e,** Virus copy number in mice brains at Day 4 post infection (n = 4, 5, 5, 4, and 2 for group A-E). **f,** IHC analysis of virus nucleocapsid protein in brains at Day 4 post infection (n = 4, 5, 5, 4, and 2 for group A-E). RTV represents ritonavir (50 mg/kg). The results of virus copy numbers were plotted as the mean ± SD. The scale bars for IHC staining represent 200 μm. Statistical analysis was performed by one-way ANOVA. *p<0.05, **p<0.01, and ***p< 0.001. Source data are provided as a Source Data file.

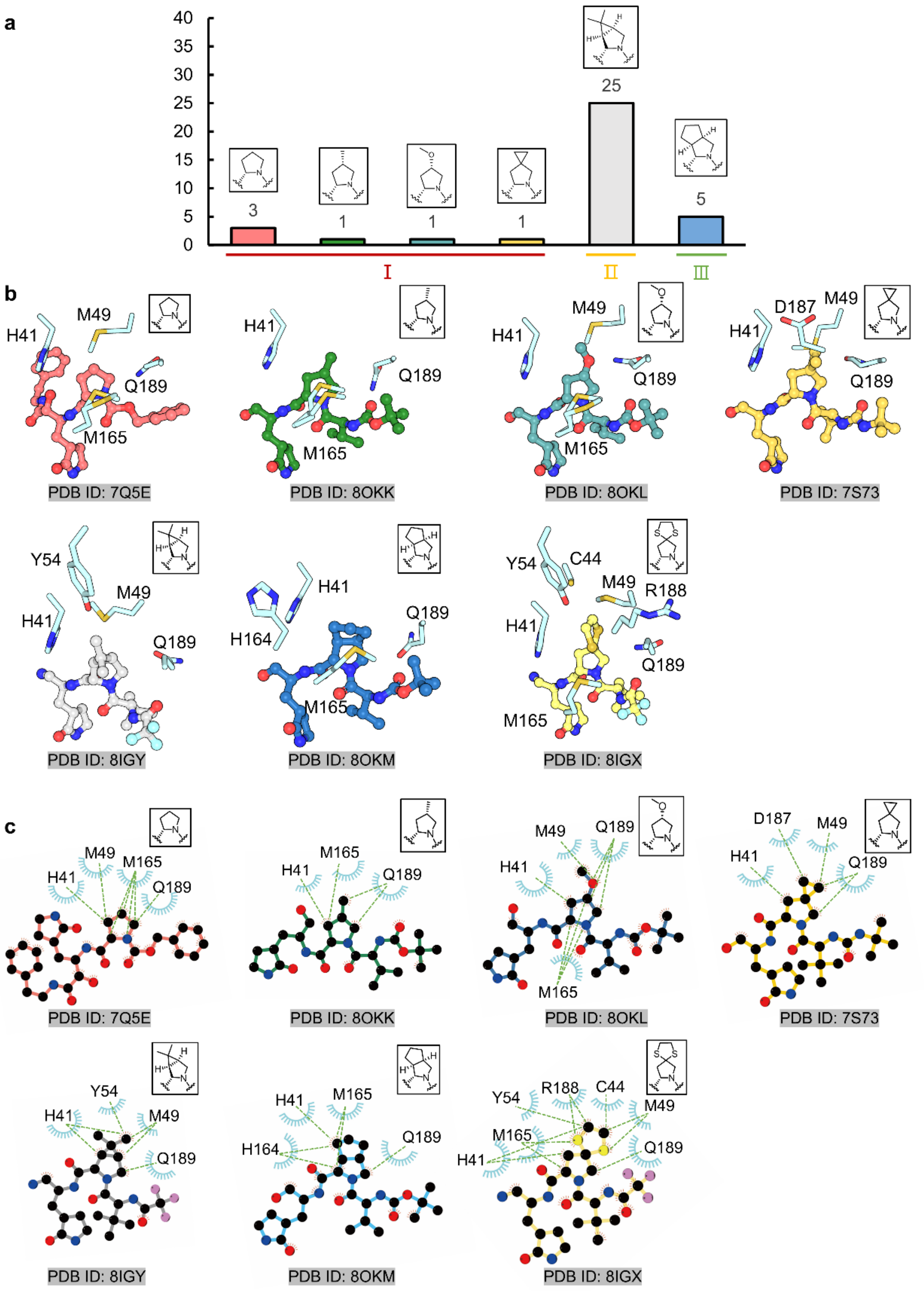

Supplementary Fig. 14 | The cyclic P2 segments of SARS-CoV-2 3CL^{pro} peptidomimetic inhibitors and their binding modes with SARS-CoV-2 3CL^{pro}. a, Classification of P2 segments of peptidomimetic inhibitors by analysis of 161 structures of the SARS-CoV-2 3CL^{pro} in complex with peptidomimetic inhibitors available in Protein Data Bank on June 3, 2023 (PDB codes: 7Q5E, 7Q5F, 8OKN, 8OKK, 8OKL,

7S73, 7EF3, 7SFB, 7SFH, 7SFI, 7S6W, 7S6Y, 7S70, 7S71, 7S72, 7S75, 7SF1, 7RFR, 7RFS, 7RFU, 6XQT, 7S6Z, 7SDC, 7TDU, 7TEH, 7TFR, 8B2T, 6WNP, 7LYI, 8DOX, 8DPR, 8OKM, 8IGN, 6XQS, 7D3I, 7LYH, 7RVP, 7JPZ, 7MAT, 7JQ1, 7JQ4, 7MAU, 7MAV, 7MB0, 7MB1, 7MB2, 7MB3, 6M0K, 7L8I, 7MAX, 7MAZ, 7RVU, 7RVV, 7SD9, 7SDA, 7RVM, 7RVO, 7RVQ, 7RVR, 7RVT, 7S74, 7RVS, 7RVX, 7UUC, 7VVP, 6Y2F, 7LCO, 7TIA, 7MAW, 8HHU, 7SH7, 7SH9, 7RVW, 7RVY, 7RW0, 7C8T, 7TJ0, 6LZE, 7SH8, 7JQ2, 7TIV, 7SGH, 7RVZ, 7R7H, 7RVN, 7RW1, 7WO1, 7WO2, 7WO3, 7WOH, 6WTJ, 6XMK, 7DGI, 7E19, 7TIW, 7TIX, 7TIZ, 7TQ2, 7TQ3, 7TQ4, 7TQ5, 7TQ6, 7XRS, 8CZW, 8CZX, 7BE7, 7DGH, 7T4A, 7T4B, 7T42, 7T43, 7T44, 7T45, 7T46, 7T48, 7T49, 7XAR, 8DD9, 7LZU, 7LZV, 7LZX, 7LZY, 7M01, 7M02, 7M03, 7M04, 6WTK, 6XHM, 6XR3, 7C8R, 7JP0, 7JQ0, 7JQ3, 7K0F, 7LCR, 7LCT, 7LDL, 7LKR, 7LKS, 7LKT, 7LKV, 7LKW, 7LKX, 7M00, 7TIU, 7TIY, 8DZB, 8E5X, 8E5Z, 8E6A, 8E61, 8F44, 8F45, 8F46, 8DOY, 7LZT, 7LZZ, 7MBI, 7ZQV, 7DGF, and 7WOF).
b, c, Hydrophobic interactions between the cyclic P2 segments of peptidomimetic inhibitors and the S2 subsite residues of SARS-CoV-2 $3CL^{pro}$.

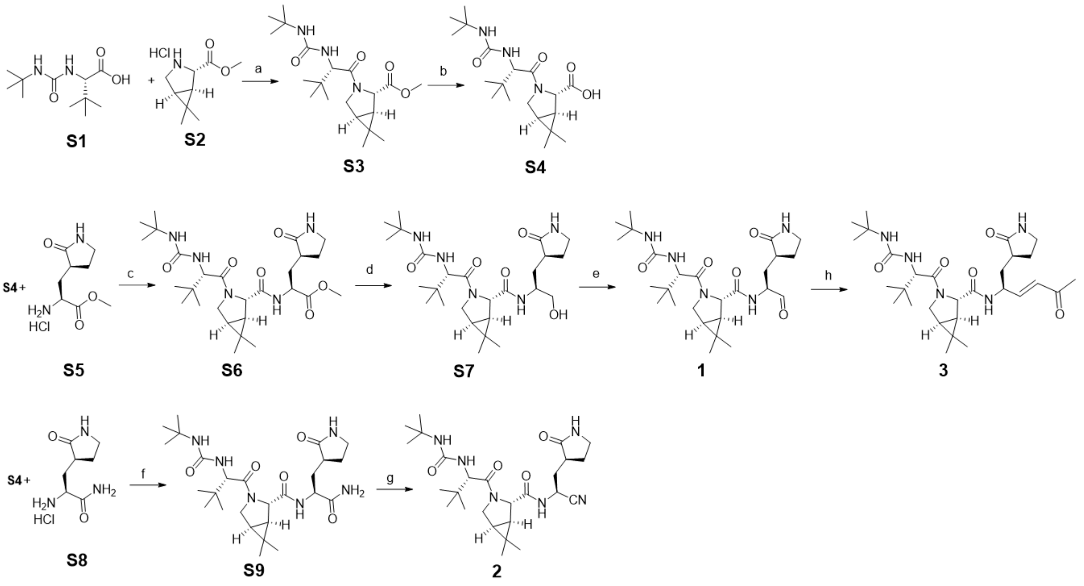

Supplementary Fig. 15 | Procedure and synthetic scheme for compounds 1-3. Reagents and conditions: (a) 2-(7-Azabenzotriazol-1-yl)-*N,N,N′,N′*-tetramethyluronium hexafluorophosphate (HATU), *N,N*-diisopropylethylamine (DIPEA), dichloromethane (DCM), 82%; (b) Lithium hydroxide monohydrate, H_2O, MeOH, tetrahydrofuran (THF), 93%; (c) HATU, DIPEA, DCM, 88%; (d) $NaBH_4$, THF, MeOH, 89%; (e) Dess-Martin Periodinane (DMP), $NaHCO_3$, DCM, 84%; (f) HATU, DIPEA, DCM, 34%; (g) Burgess Reagent, DCM, 51%; (h) Diethyl (2-oxopropyl)phosphonate, K_2CO_3, THF, 28%.

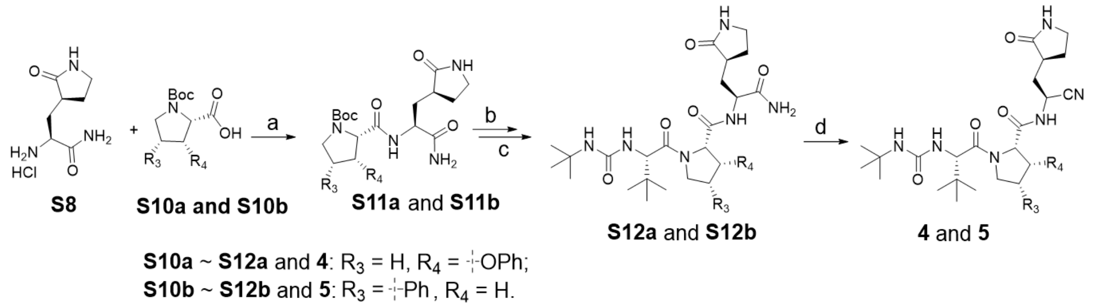

Supplementary Fig. 16 | Procedure and synthetic scheme for compounds 4 and 5. Reagents and conditions: (a) HATU, DIPEA DCM, *N*, *N*-dimethylformamide (DMF); (b) 4 M HCl in dioxane, DCM; (c) Compound **S1**, HATU, DIPEA, DCM, DMF; (d) Burgess Reagent, DCM.

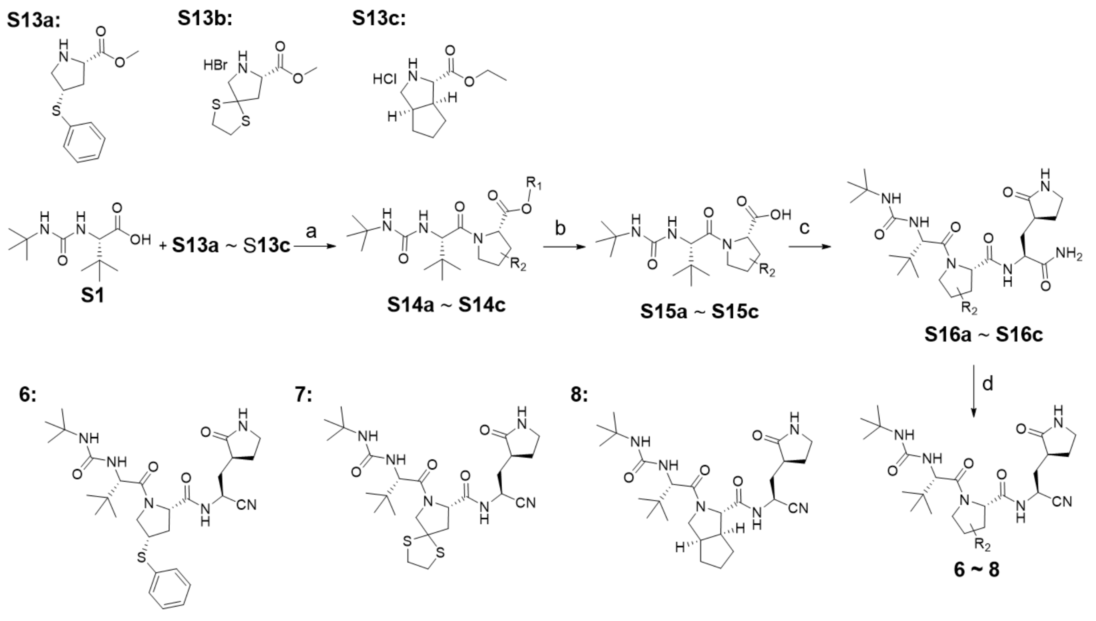

Supplementary Fig. 17 | Procedure and synthetic scheme for compounds 6-8. Reagents and conditions: (a) i. HATU, DIPEA DCM; or 1H-benzotriazol-1-yloxytris(dimethylamino)phosphonium hexafluorophosphate (BOP), 4-methylmorpholine (NMM), DCM, DMF; (b) Lithium hydroxide monohydrate, H_2O, MeOH, THF; (c) Compound **S8**, HATU, DIPEA, DCM; (d) Burgess Reagent, DCM.

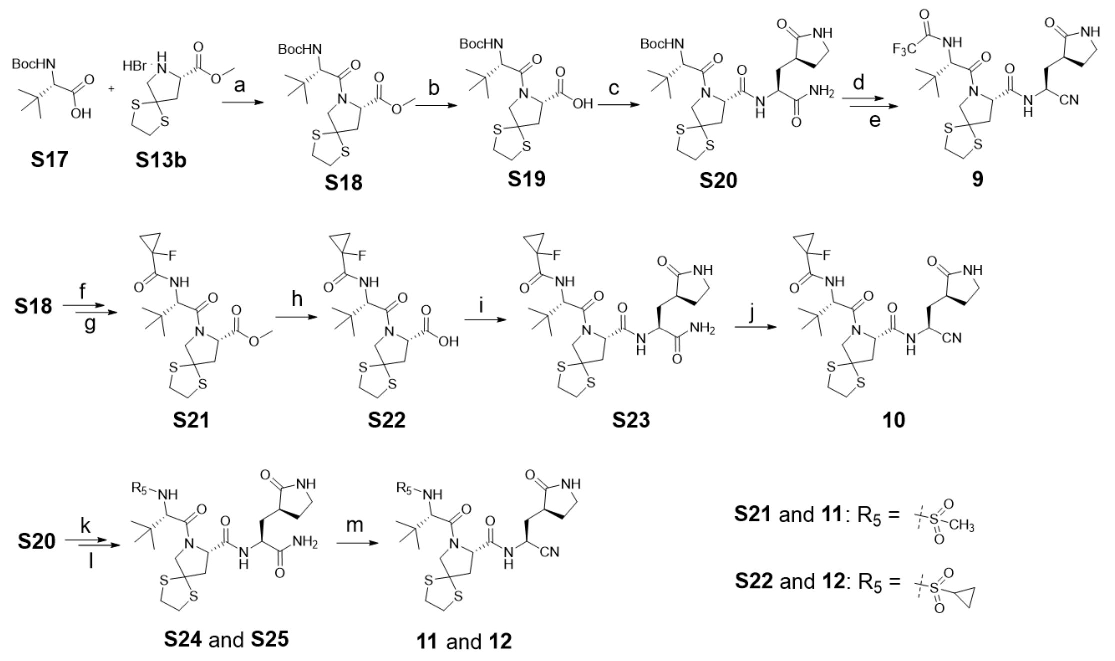

Supplementary Fig. 18 | Procedure and synthetic scheme for compounds 9-12. Reagents and conditions: (a) BOP, NMM, DCM, DMF, 60%; (b) Lithium hydroxide monohydrate, H_2O, MeOH, THF, 83%; (c) Compound **S8**, HATU, DIPEA, DCM, 40%; (d) 4 M HCl in dioxane, 1,4-dioxane; (e) triethylamine (Et_3N), trifluoroacetic anhydride, DCM, 40% over two steps; (f) 4 M HCl in dioxane, 1,4-dioxane; (g) 1-Fluoro-cyclopropanecarboxylic acid, HATU, DIPEA, DCM, 90%; (h) Lithium hydroxide monohydrate, H_2O, THF, 74%; (i) Compound **S8**, HATU, DIPEA, DCM, 60%; (j) Burgess Reagent, DCM, 41%; (k) 4 M HCl in dioxane, DCM; (l) Methanesulfonyl chloride or cyclopropanesulfonyl chloride, Et_3N, DCM; (m) Burgess Reagent, DCM.

S2: Supplementary Tables

Supplementary Table 1 | Thermodynamic profiles of compounds 2, 7 and 10-12, simnotrelvir, and nirmatrelvir binding to SARS-CoV-2 $3CL^{pro}$ measured by ITC

Compounds	K_d (nM)	ΔG (kJ/mol)	ΔH (kJ/mol)	$-T\Delta S$ (kJ/mol)
2	2968 ± 138	-32.08 ± 0.12	-7.07 ± 0.43	-25.01 ± 0.42
7	1683 ± 40	-33.51 ± 0.06	-13.35 ± 1.08	-20.16 ± 1.12
Nirmatrelvir	620 ± 58	-36.03 ± 0.24	-25.71 ± 0.35	-10.32 ± 0.30
Simnotrelvir	302 ± 49	-37.87 ± 0.39	-33.08 ± 0.59	-4.79 ± 0.36
10	680 ± 10	-35.79 ± 0.04	-26.65 ± 2.38	-9.14 ± 2.35
11	391 ± 67	-37.22 ± 0.41	-22.33 ± 1.08	-14.89 ± 1.39
12	623 ± 47	-36.02 ± 0.19	-31.06 ± 1.20	-4.96 ± 1.36

[*] Data are shown as mean ± SD of three independent experiments.

Supplementary Table 2 | Pharmacokinetic parameters resulted from single-dose intravenous and oral administration of simnotrelvir in rat and monkey.

Species	Dose (PO, mg/kg)	Dose (IV, mg/kg)	CL (mL/min/kg)	Vss (L/kg)	$t_{1/2}$ (h)	F (%)
Rat	15	15	59.3	1.36	0.45	35.3
Monkey	5	5	20.7	1.50	1.7	41.9

[*] Data are shown as mean of 6 biological replicates.

Supplementary Table 3 | Pharmacokinetic parameters resulted from oral administration of simnotrelvir with or without ritonavir in rat and monkey.

Compound	Species	Dose (mg/kg)	T_{max} (h)	C_{max} (ng/mL)	AUC_{0-t} (ng·h/mL)	$AUC_{0-\infty}$ (ng·h/mL)	$t_{1/2}$ (h)
simnotrelvir	Rat	15	0.25	1580	1700	1730	1.29
simnotrelvir +ritonavir		15	0.5	2760	8920	9000	1.99
simnotrelvir	Monkey	5	0.5	625	1660	1790	5.63
simnotrelvir +ritonavir		5	1.0	2440	17000	17200	2.38

* Data are shown as mean of 6 biological replicates.

** The dose of ritonavir administrated to rat and monkey is 30 and 15 mg/kg, respectively.

Supplementary Table 4 | Crystallography data collection and refinement statistics of SARS-CoV-2 3CL^{pro} in complex with seven compounds.

**Compounds**	**1**	**2**	**3**
**PDB ID**	8IFP	8IFQ	8IFR
Space Group	P 21 21 2	P 21 21 2	P 21 21 2
Cell Dimension: a (Å)	45.678	45.564	45.582
b (Å)	63.427	63.918	63.707
c (Å)	105.59	105.364	105.502
Wavelength (Å)	0.979	0.979	0.979
Reflections (unique)	29403 (2952)	21717 (2179)	36576 (3547)
Resolution Range (Å)	27.88-1.78	27.82-1.96	30.79-1.66
Highest-Resolution Shell (Å)	1.84-1.78	2.03-1.96	1.72-1.66
Redundancy	6.8 (7.2)	6.4 (6.5)	9.4 (9.9)
I/σ (I)	15.75 (2.31)	14.48 (3.33)	14.05 (2.20)
Highest-Resolution Shell $CC_{1/2}$	0.765	0.888	0.600
Completeness (%)	94.9 (99.6)	95.1 (98.3)	98.9 (98.1)
Rwork/Rfree	0.266/0.293	0.179/0.214	0.181/0.215
**RMS Values**			
Bond length (Å)	0.003	0.008	0.007
Bond angle (°)	0.615	0.977	0.870
**Numbers of Non-hydrogen Atoms**			
Protein	2294	2331	2323
Inhibitor	36	36	39
Water Oxygen	191	212	222
Others	0	0	0
Clashscore	3.09	1.94	2.81
MolProbity Score	1.17	0.96	1.07
**B-factor (Å^2)**			
Protein	25.02	24.79	27.43
Inhibitor	19.80	20.12	20.67
Water Oxygen	25.66	31.97	37.30
**Ramachandran plot**			
Favored (%)	97.69	98.02	98.68
Allowed (%)	2.31	1.65	1.32
Outliers (%)	0	0.33	0

Supplementary Table 4 (continued) | Crystallography data collection and refinement statistics of SARS-CoV-2 $3CL^{pro}$ in complex with seven compounds.

Compounds	7	10	Simnotrelvir	Nirmatrelvir
**PDB ID**	8IFS	8IFT	8IGX	8IGY
Space Group	P 1 21 1	P 21 21 2	P 21 21 2	P 21 21 2
Cell Dimension: a (Å)	54.542	45.571	45.578	45.582
b (Å)	82.106	63.153	62.831	63.619
c (Å)	83.394	105.683	105.323	105.282
Wavelength (Å)	0.979	0.979	0.979	0.979
Reflections (unique)	27013 (2752)	29018 (2852)	24454 (2386)	20923 (2183)
Resolution Range (Å)	27.00-2.46	31.58-1.80	21.42-1.90	27.81-1.96
Highest-Resolution Shell (Å)	2.55-2.46	1.86-1.80	1.97-1.90	2.03-1.96
Redundancy	6.5 (6.7)	12.9 (12.8)	12.6 (11.1)	9.0 (7.8)
I/σ (I)	9.57 (2.38)	10.52 (1.66)	9.33(1.95)	15.93 (2.68)
Highest-Resolution Shell $CC_{1/2}$	0.954	0.752	0.787	0.828
Completeness (%)	95.0 (98.6)	99.9 (100.0)	99.5 (99.8)	92.2 (99.8)
Rwork/Rfree	0.266/0.296	0.184/0.204	0.176/0.213	0.187/0.205
**RMS Values**				
Bond length (Å)	0.004	0.009	0.009	0.006
Bond angle (°)	0.662	0.981	0.897	0.804
**Numbers of Non-hydrogen Atoms**				
Protein	4454	2328	2335	2287
Inhibitor	74	36	36	35
Water Oxygen	9	215	167	118
Others	0	0	0	0
Clashscore	1.72	1.95	1.72	2.44
MolProbity Score	0.93	1.13	0.93	1.03
**B-factor ($Å^2$)**				
Protein	61.74	28.17	27.17	27.22
Inhibitor	58.33	22.74	24.65	25.08
Water Oxygen	57.96	36.46	33.66	36.85
**Ramachandran plot**				
Favored (%)	97.82	97.04	98.02	98.02
Allowed (%)	2.18	2.96	1.98	1.98
Outliers (%)	0	0	0	0

Supplementary Table 5 | The scoring system for lung histopathology assessment.

Score	0	1	2	3	4
**Alveolar atrophy or dilatation**	**None**	Mild, < 10% area	Moderate, 10% to 25% area	Significant, 25% to 50% area	Severe, ≥ 50% area
**Alveolar hemorrhage**	**None**	Mild, < 10% area	Moderate, 10% to 25% area	Significant, 25% to 50% area	Severe, ≥ 50% area
**Alveolar wall thickening**	**None**	Mild, < 10% area	Moderate, 10% to 25% area	Significant, 25% to 50% area	Severe, ≥ 50% area
**Infiltration of inflammatory cells**	**None**	Few inflammatory cell accumulation near blood vessels	Obvious inflammatory cell surrounding blood vessels and alveolar spaces	Massive inflammatory cell in alveolar and interstitial locations	/

Supplementary Table 6 | The scoring system for brain histopathology assessment.

Score	0	1	2	3	4
**Bleeding**	None	< 10% area	11% to 50% area	51% to 75% area	>75% area
**Neuron degeneration in hippocampus**	None	Few, < 5% area	Obvious, 5% to 25% area	Frequent, >25% area	/
**Neuron degeneration in cortex**	None	Few, < 5% area	Obvious, 5% to 25% area	Frequent, >25% area	/

S3: Spectral Data for Synthetic Compounds

Methyl (1R,2S,5S)-3-((S)-2-(3-(tert-butyl)ureido)-3,3-dimethylbutanoyl)-6,6-dimethyl-3-azabicyclo[3.1.0]hexane-2-carboxylate (S3). Compound **S1** (575 mg, 2.5 mmol) and compound **S2** (513 mg, 2.5 mmol) were dissolved in DCM (20 mL) under N_2 atmosphere, HATU (1 g, 2.6 mmol) was added into the reaction and stirred at 25 °C for 1 h. Then DIPEA (1.30 mL, 7.5 mmol) was added to the reaction and stirred at 25 °C for 4 h. DCM (50 mL) and 1N HCl aqueous solution (25 mL) were added to the reaction. After separation, the organic phase was washed with water (25 mL), saturated brine (25 mL), and dried over anhydrous sodium sulphate, evaporated in vacuum and purified by column chromatography to afford the compound **S3** as white solid (780 mg, yield: 82%). ^{1}H NMR (500 MHz, DMSO-d_6) δ 5.96 (s, 1H), 5.90 (d, J = 10.0 Hz, 1H), 4.18 (s, 1H), 4.15 (d, J = 9.9 Hz, 1H), 3.77 (dd, J = 10.3, 5.3 Hz, 1H), 3.64 (s, 3H), 1.51 (dd, J = 7.5, 5.2 Hz, 1H), 1.40 (d, J = 7.5 Hz, 1H), 1.17 (d, J = 2.9 Hz, 10H), 1.00 (s, 3H), 0.91 (s, 9H), 0.82 (s, 3H). ^{13}C NMR (101 MHz, DMSO-d_6) δ 172.04, 171.76, 157.67, 59.01, 57.03, 52.35, 49.44, 47.44, 34.48, 30.06, 29.55, 27.42, 26.68, 26.25, 19.38, 12.69. ESI-HRMS Calcd for $C_{20}H_{36}N_3O_4$ [M+H]$^+$: 382.2700, found 382.2700.

(1R,2S,5S)-3-((S)-2-(3-(tert-butyl)ureido)-3,3-dimethylbutanoyl)-6,6-dimethyl-3-azabicyclo[3.1.0]hexane-2-carboxylic acid (S4). Compound **S3** (620 mg, 1.6 mmol) was dissolved in THF (4 mL), and then lithium hydroxide monohydrate (76 mg, 1.8 mmol), water (2 mL) and MeOH (2 mL) were added. The mixture was stirred at 45 °C for 1 h. When reaction completed, most of the MeOH and THF were evaporated under vacuum, water (5 mL) and 1N HCl aqueous solution (about 1.8 mL) was added to the reaction, white solid was precipitated and filtered. The solid was washed by water and dried in vacuum to give the compound **S4** (547 mg, yield: 93%). ^{1}H NMR (400 MHz, DMSO-d_6) δ 12.63 (s, 1H), 5.96 (s, 1H), 5.89 (d, J = 10.1 Hz, 1H), 4.15 (d, J = 10.0 Hz, 1H), 4.10 (d, J = 2.4 Hz, 1H), 3.99 (d, J = 10.5 Hz, 1H), 3.79-3.67 (m, 1H), 1.47 (d, J = 5.9 Hz, 1H), 1.38 (d, J = 7.6 Hz, 1H), 1.16 (d, J = 2.5 Hz, 9H), 1.00 (d, J = 2.4 Hz, 3H), 0.91 (s, 9H), 0.81 (d, J = 2.4 Hz, 3H). ^{13}C NMR (101 MHz, DMSO-d_6) δ 173.16, 171.59, 157.69, 59.17, 57.02, 49.42, 47.43, 34.57, 30.29, 29.57, 27.29, 26.77, 26.34, 19.23, 12.77. ESI-HRMS Calcd for $C_{19}H_{34}N_3O_4$ [M+H]$^+$: 368.2544, found 368.2547.

Methyl (S)-2-((1R,2S,5S)-3-((S)-2-(3-(tert-butyl)ureido)-3,3-dimethylbutanoyl)-6,6-dimethyl-3-azabicyclo[3.1.0]hexane-2-carboxamido)-3-((S)-2-oxopyrrolidin-3-yl)propanoate (S6). Compound **S6** was prepared according to the procedure of compound **S3**, white solid, yield: 88%. ^{1}H NMR (500 MHz, DMSO-d_6) δ 8.55 (d, J = 8.6 Hz, 1H), 7.58 (s, 1H), 5.94 (s, 1H), 5.85 (d, J = 9.9 Hz, 1H), 4.43 (ddd, J = 12.2, 8.6, 3.9 Hz, 1H), 4.22 (s, 1H), 4.10 (d, J = 9.9 Hz, 1H), 3.93 (d, J = 10.1 Hz, 1H), 3.80

(dd, *J* = 10.1, 5.4 Hz, 1H), 3.64 (s, 3H), 3.14 (t, *J* = 9.2 Hz, 1H), 3.03 (td, *J* = 9.3, 6.9 Hz, 1H), 2.44 (ddd, *J* = 11.3, 8.3, 3.4 Hz, 1H), 2.16-2.00 (m, 2H), 1.58 (tdd, *J* = 13.7, 11.6, 6.6 Hz, 2H), 1.48 (dd, *J* = 7.6, 5.3 Hz, 1H), 1.23 (d, *J* = 7.7 Hz, 1H), 1.17 (s, 9H), 1.02 (s, 3H), 0.89 (s, 9H), 0.86 (s, 3H). ^{13}C NMR (101 MHz, DMSO-d_6) δ 178.69, 173.58, 170.98, 170.88, 157.39, 59.89, 56.78, 50.42, 48.94, 47.45, 37.29, 34.06, 33.96, 30.51, 29.11, 27.45, 27.26, 26.40, 26.01, 18.57, 12.59. ESI-HRMS Calcd for $C_{27}H_{46}N_5O_6$ $[M+H]^+$: 536.3443, found 536.3446.

(1R,2S,5S)-3-((S)-2-(3-(tert-butyl)ureido)-3,3-dimethylbutanoyl)-N-((S)-1-hydroxy-3-((S)-2-oxopyrrolidin-3-yl)propan-2-yl)-6,6-dimethyl-3-azabicyclo[3.1.0]hexane-2-carboxamide (S7). Compound **S6** (462 mg, 0.86 mmol) was dissolved in THF (10 mL). $NaBH_4$ (198 mg, 5.2 mmol) was added in batches to the reaction under ice bath, and then anhydrous MeOH (1 mL) was added drop by drop slowly. Removed the ice bath, the reaction was stirred at 25 °C for 2 h. NH_4Cl saturated solution (10 mL) and ethyl acetate (EA, 10 mL) were added and then separated. The aqueous phase was extrated by EA (10 mL) twice. The combined EA was washed by with saturated brine (20 mL), dried over anhydrous sodium sulphate, evaporated in vacuum and purified by column chromatography to obtain the compound **S7** as white solid (390 mg，yield: 89%). ^{1}H NMR (500 MHz, DMSO-d_6) δ 7.77 (d, *J* = 9.4 Hz, 1H), 7.43 (s, 1H), 5.93 (s, 1H), 5.84 (d, *J* = 10.0 Hz, 1H), 4.65 (t, *J* = 5.7 Hz, 1H), 4.13 (s, 1H), 4.08 (d, *J* = 9.9 Hz, 1H), 3.90 (d, *J* = 10.2 Hz, 1H), 3.79 (dd, *J* = 10.1, 5.4 Hz, 2H), 3.25 (dt, *J* = 10.5, 6.2 Hz, 1H), 3.16 (d, *J* = 5.0 Hz, 1H), 3.09 (t, *J* = 9.1 Hz, 1H), 2.97 (td, *J* = 9.4, 7.0 Hz, 1H), 2.47 – 2.34 (m, 1H), 2.16 (dt, *J* = 14.6, 7.7 Hz, 1H), 1.75-1.68 (m, 1H), 1.57-1.40 (m, 2H), 1.32-1.24 (m, 2H), 1.15 (s, 9H), 0.99 (s, 3H), 0.89 (s, 9H), 0.84 (s, 3H). ^{13}C NMR (101 MHz, DMSO-d_6) δ 179.66, 171.31, 171.17, 157.92, 64.64, 60.50, 57.33, 49.39, 48.78, 47.93, 37.52, 34.55, 33.50, 31.23, 29.57, 28.31, 27.80, 26.90, 26.49, 19.03, 13.08. ESI-HRMS Calcd for $C_{26}H_{46}N_5O_5$ $[M+H]^+$: 508.3493, found 508.3499.

(1R,2S,5S)-3-((S)-2-(3-(tert-butyl)ureido)-3,3-dimethylbutanoyl)-6,6-dimethyl-N-((S)-1-oxo-3-((S)-2-oxopyrrolidin-3-yl)propan-2-yl)-3-azabicyclo[3.1.0]hexane-2-carboxamide (1). Compound **S7** (230 mg, 0.45 mmol) was dissolved in dried DCM (8 mL) under N_2 atmosphere. DMP (207 mg, 0.49 mmol) and $NaHCO_3$ (82 mg, 0.98 mmol) were added to the solution. The reaction was stirred at 25 °C for 2 h. The solution was filtered, and the filter cake was washed by DCM (10 mL). The organic phase was washed by $Na_2S_2O_3$ saturated solution (10 mL), $NaHCO_3$ saturated solution (10 mL), saturated brine (10 mL) and dried over anhydrous sodium sulphate, evaporated in vacuum and purified by column chromatography to afford the compound **1** as white solid (192 mg, yield: 84%). ^{1}H NMR (400 MHz, DMSO-d_6) δ 9.47 (s, 1H), 8.52 (d, *J* = 8.2 Hz, 1H), 7.58 (s, 1H), 5.95 (d, *J* = 7.7 Hz, 1H), 5.86 (d, *J* = 9.8 Hz, 1H), 4.32 (t,

J = 10.1 Hz, 1H), 4.22 (s, 1H), 4.10 (d, J = 9.8 Hz, 1H), 3.94 (d, J = 10.3 Hz, 1H), 3.81 (t, J = 7.5 Hz, 1H), 3.15-3.03 (m, 2H), 2.43 (d, J = 10.2 Hz, 1H), 2.13 (s, 2H), 1.84 (s, 1H), 1.59 (s, 1H), 1.50 (d, J = 7.1 Hz, 1H), 1.33 (s, 1H), 1.17 (s, 9H), 1.02 (s, 3H), 0.89 (s, 9H), 0.86 (s, 3H). ^{13}C NMR (101 MHz, DMSO-d_6) δ 201.60, 178.84, 172.12, 171.37, 157.86, 60.25, 57.29, 56.30, 49.42, 47.85, 37.69, 37.36, 34.51, 31.20, 30.20, 29.78, 29.58, 27.85, 26.86, 26.45, 19.19, 13.03. ESI-HRMS Calcd for $C_{26}H_{44}N_5O_5$ [M+H]$^+$: 506.3337, found 506.3342. HPLC purity 98.5% (Rt = 4.688 min, Method A).

(1R,2S,5S)-N-((S)-1-amino-1-oxo-3-((S)-2-oxopyrrolidin-3-yl)propan-2-yl)-3-((S)-2-(3-(tert-butyl)ureido)-3,3-dimethylbutanoyl)-6,6-dimethyl-3-azabicyclo[3.1.0]hexane-2-carboxamide (S9). Compound **S9** was prepared according to the procedure of compound **S3**, white solid, yield: 34%. ^{1}H NMR (400 MHz, DMSO-d_6) δ 8.20 (d, J = 8.7 Hz, 1H), 7.53 (s, 1H), 7.36-7.20 (m, 1H), 7.11-6.93 (m, 1H), 5.93 (s, 1H), 5.85 (d, J = 9.9 Hz, 1H), 4.27 (td, J = 8.6, 4.3 Hz, 1H), 4.21 (s, 1H), 4.11 (d, J = 9.9 Hz, 1H), 3.92 (d, J = 10.2 Hz, 1H), 3.79 (dd, J = 10.1, 5.3 Hz, 1H), 3.12 (t, J = 9.0 Hz, 1H), 3.02 (td, J = 9.3, 7.0 Hz, 1H), 2.42 (dq, J = 14.4, 5.7, 3.7 Hz, 1H), 2.19-2.06 (m, 1H), 1.94 (ddd, J = 13.5, 11.9, 3.7 Hz, 1H), 1.67-1.57 (m, 1H), 1.57-1.47 (m, 1H), 1.47-1.41 (m, 1H), 1.34 (d, J = 7.7 Hz, 1H), 1.16 (s, 9H), 1.00 (s, 3H), 0.89 (s, 9H), 0.84 (s, 3H). ^{13}C NMR (101 MHz, DMSO-d_6) δ 178.69, 173.58, 170.98, 170.88, 157.39, 59.89, 56.78, 50.42, 48.94, 47.45, 37.29, 34.06, 33.96, 30.51, 29.11, 27.45, 27.26, 26.40, 26.01, 18.57, 12.59. ESI-HRMS Calcd for $C_{26}H_{45}N_6O_5$ [M+H]$^+$: 521.3446, found 521.3456.

(1R,2S,5S)-3-((S)-2-(3-(tert-butyl)ureido)-3,3-dimethylbutanoyl)-N-((S)-1-cyano-2-((S)-2-oxopyrrolidin-3-yl)ethyl)-6,6-dimethyl-3-azabicyclo[3.1.0]hexane-2-carboxamide (2). Compound **S9** (226 mg, 0.43 mmol) was dissolved in dried DCM (5 mL) under N_2 atmosphere. Burgess Reagent (119 mg, 0.5 mmol) was added and stirred at 25 °C for 10 h. The mixture was concentrated and purified by column chromatography to give the compound **2** as white solid (110 mg, yield: 51%). ^{1}H NMR (600 MHz, DMSO-d_6) δ 8.95 (d, J = 8.6 Hz, 1H), 7.65 (s, 1H), 5.93 (s, 1H), 5.85 (d, J = 10.1 Hz, 1H), 4.95 (ddd, J = 11.0, 8.6, 5.2 Hz, 1H), 4.12 (s, 1H), 4.09 (d, J = 10.0 Hz, 1H), 3.94 (d, J = 10.2 Hz, 1H), 3.81 (dd, J = 10.3, 5.5 Hz, 1H), 3.17-3.10 (m, 1H), 3.03 (td, J = 9.3, 7.0 Hz, 1H), 2.44 (tdd, J = 10.3, 8.3, 4.4 Hz, 1H), 2.15 (ddd, J = 13.5, 11.0, 4.4 Hz, 1H), 2.11-2.02 (m, 1H), 1.74-1.63 (m, 2H), 1.52 (dd, J = 7.7, 5.4 Hz, 1H), 1.26 (d, J = 7.6 Hz, 1H), 1.16 (s, 9H), 1.01 (s, 3H), 0.88 (s, 9H), 0.85 (s, 3H). ^{13}C NMR (101 MHz, DMSO-d_6) δ 178.01, 171.52, 171.49, 157.87, 120.15, 60.10, 57.30, 55.38, 49.43, 47.85, 38.19, 37.13, 34.57, 34.45, 30.78, 29.57, 27.95, 27.34, 26.84, 26.34, 19.30, 12.99. ESI-HRMS Calcd for $C_{26}H_{43}N_6O_4$ [M+H]$^+$: 503.3340, found 503.3338. HPLC purity 99.1% (Rt = 9.530 min, Method B).

(1R,2S,5S)-3-((S)-2-(3-(tert-butyl)ureido)-3,3-dimethylbutanoyl)-6,6-dimethyl-N-((S,E)-5-oxo-1-((S)-2-oxopyrrolidin-3-yl)hex-3-en-2-yl)-3-azabicyclo[3.1.0]hexane-2-carboxamide (3). Diethyl (2-oxopropyl)phosphonate (72 μL, 0.41 mmol) and K_2CO_3 (170 mg, 1.23 mmol) were dissolved in anhydrous THF (10 mL) under N_2 atmosphere. The mixture was stirred for 0.5 h under reflux and then compound **1** (173 mg, 0.34 mmol) in THF (1 mL) was added. The reaction was stirred for 2 h under reflux. When the reaction completed, water (10 mL) and EA (10 mL) were added to the solution and then separated. The aqueous phase was extrated by EA (10 mL) twice. The combined EA was washed by with saturated brine (20 mL), dried over anhydrous sodium sulphate, evaporated in vacuum and purified by column chromatography to give the compound **3** as white solid (52 mg, yield: 28%). ^{1}H NMR (500 MHz, DMSO-d_6) δ 8.30 (d, J = 9.0 Hz, 1H), 7.54 (s, 1H), 6.84 (dd, J = 16.1, 4.7 Hz, 1H), 6.05 (dd, J = 16.2, 1.7 Hz, 1H), 5.94 (s, 1H), 5.87 (d, J = 9.9 Hz, 1H), 4.56 (d, J = 10.9 Hz, 1H), 4.20 (s, 1H), 4.10 (d, J = 9.5 Hz, 1H), 3.93 (d, J = 10.2 Hz, 1H), 3.82 (dd, J = 10.1, 5.4 Hz, 1H), 3.13 (t, J = 9.2 Hz, 1H), 3.02 (td, J = 9.3, 7.0 Hz, 1H), 2.22 (s, 3H), 2.14 (dt, J = 14.1, 7.7 Hz, 1H), 1.86 (td, J = 13.1, 3.4 Hz, 1H), 1.68-1.56 (m, 1H), 1.53-1.40 (m, 2H), 1.24 (d, J = 7.6 Hz, 1H), 1.17 (s, 10H), 1.02 (s, 3H), 0.90 (s, 9H), 0.87 (s, 3H). ^{13}C NMR (201 MHz, DMSO-d_6) δ 198.63, 179.05, 171.36, 157.93, 149.01, 129.37, 60.42, 57.34, 49.42, 47.89, 47.53, 37.75, 36.02, 34.52, 31.20, 29.57, 27.94, 27.90, 27.40, 26.89, 26.47, 19.19, 13.07, 0.59. ESI-HRMS Calcd for $C_{29}H_{48}N_5O_5$ $[M+H]^+$: 546.3650, found 546.3649. HPLC purity 98.0% (Rt = 7.554 min, Method C).

Tert-butyl (2S,3R)-2-(((S)-1-amino-1-oxo-3-((S)-2-oxopyrrolidin-3-yl)propan-2-yl)carbamoyl)-3-phenoxypyrrolidine-1-carboxylate (S11a). Compound **S8** (311 mg, 1.5 mmol) and compound **S10a** (460 mg, 1.5 mmol) were dissolved in DCM (8 mL) and DMF (8 mL), HATU (570 mg, 1.5 mmol) was added into the reaction and stirred at 25 °C for 1 h. Then DIPEA (0.78 mL, 4.5 mmol) was added to the reaction and stirred at 25 °C for 2 h. When the reaction completed, DCM (10 mL) and 1N HCl aqueous solution (20 mL) were added to the reaction. After separation, the organic phase was washed with water (20 mL)) twice, the organic phase was washed with saturated brine (25 mL), dried over anhydrous sodium sulphate, evaporated in vacuum and purified by column chromatography to afford the compound **S11a** as white solid (490 mg, yield: 71%). ^{1}H NMR (500 MHz, DMSO-d_6) δ 8.17 (d, J = 8.6 Hz, 1H), 7.62 (d, J = 8.8 Hz, 1H), 7.35-7.11 (m, 4H), 6.93 (t, J = 7.3 Hz, 1H), 6.88 (t, J = 7.9 Hz, 2H), 4.97-4.89 (m, 1H), 4.33-4.19 (m, 2H), 3.76 (dd, J = 12.1, 5.1 Hz, 1H), 3.57-3.40 (m, 1H), 3.11 (d, J = 9.6 Hz, 1H), 2.93 (dt, J = 17.3, 8.5 Hz, 1H), 2.61 (dd, J = 9.7, 5.0 Hz, 1H), 2.30-2.16 (m, 1H), 2.15-1.94 (m, 3H), 1.74-1.45 (m, 2H), 1.42 (s, 9H). ^{13}C NMR (101 MHz, DMSO-d_6) δ 178.72, 174.10, 172.12, 157.37, 154.09, 129.98, 121.29, 115.92, 80.06,

75.67, 59.66, 51.27, 45.96, 38.19, 33.59, 28.51, 28.02, 10.72. ESI-HRMS Calcd for $C_{23}H_{33}N_4O_6$ [M+H]$^+$: 461.2395, found 461.2399.

(2S,3R)-N-((S)-1-amino-1-oxo-3-((S)-2-oxopyrrolidin-3-yl)propan-2-yl)-1-((S)-2-(3-(tert-butyl)ureido)-3,3-dimethylbutanoyl)-3-phenoxypyrrolidine-2-carboxamide (S12a). Compound **S11a** (189 mg, 0.41 mmol) and 4 M HCl in 1,4-dioxane (1 mL) were dissolved in DCM (1 mL), the reaction was stirred at 25 °C for 0.5 h. And then the solution was concentrated to remove the solvent, dry HCl salt was obtained. On the other hand, compound **1** (95 mg, 0.41 mmol) and HATU (156 mg, 0.41 mmol) were dissolved in DCM (8 mL) and DMF (8 mL) under N_2 atmosphere, the reaction was stirred at 25 °C for 0.5 h. Then DIPEA (0.22 mL, 1.23 mmol), and HCl salt were added to the reaction, the reaction mixture was stirred for 10 h at 25 °C. Then DCM (20 mL) and 1N HCl aqueous solution (20 mL) were added to the reaction. After separation, the organic phase was washed with water (20 mL) twice, the organic phase was washed with saturated brine (20 mL), dried over anhydrous sodium sulphate, evaporated in vacuum and purified by column chromatography to give compound **S12a** as white solid (196 mg, yield: 83%).^{1}H NMR (400 MHz, DMSO-d_6) δ 8.02 (d, J = 8.7 Hz, 1H), 7.56 (s, 1H), 7.36-7.23 (m, 3H), 7.07 (s, 1H), 6.96 (d, J = 7.7 Hz, 3H), 6.02 (d, J = 9.3 Hz, 1H), 5.97 (s, 1H), 5.04 (t, J = 6.3 Hz, 1H), 4.41 (t, J = 7.9 Hz, 1H), 4.31 (dd, J = 10.5, 5.5 Hz, 2H), 4.22 (d, J = 9.2 Hz, 1H), 3.58 (dd, J = 10.9, 5.7 Hz, 1H), 3.12 (t, J = 9.2 Hz, 1H), 3.01 (q, J = 8.7 Hz, 1H), 2.60 (dd, J = 13.9, 7.3 Hz, 1H), 2.44 (d, J = 10.2 Hz, 1H), 2.18 (d, J = 8.6 Hz, 1H), 2.01-1.89 (m, 2H), 1.64 (p, J = 9.2, 8.4 Hz, 1H), 1.49 (t, J = 12.2 Hz, 1H), 1.20 (s, 9H), 0.91 (s, 9H). ^{13}C NMR (126 MHz, DMSO-*d6*) δ 178.76, 173.48, 171.43, 170.81, 157.11, 129.62, 121.18, 115.59, 74.60, 58.39, 56.73, 52.44, 50.50, 49.04, 37.42, 34.99, 34.40, 34.24, 29.31, 27.60, 26.33. ESI-HRMS Calcd for $C_{29}H_{45}N_6O_6$ [M+H]$^+$: 573.3395, found 573.3397.

(2S,3R)-1-((S)-2-(3-(tert-butyl)ureido)-3,3-dimethylbutanoyl)-N-((S)-1-cyano-2-((S)-2-oxopyrrolidin-3-yl)ethyl)-3-phenoxypyrrolidine-2-carboxamide (4). Compound **4** was prepared according to the procedure of compound **2**, white solid, yield: 38%. ^{1}H NMR (500 MHz, DMSO-d_6) δ 8.80 (dd, J = 8.6, 3.3 Hz, 1H), 7.65 (s, 1H), 7.29 (t, J = 7.8 Hz, 2H), 6.97 (d, J = 7.8 Hz, 3H), 6.04-5.92 (m, 2H), 5.16-4.90 (m, 2H), 4.33 (ddd, J = 25.8, 9.4, 6.4 Hz, 2H), 4.21 (d, J = 9.5 Hz, 1H), 3.59 (dd, J = 10.7, 5.4 Hz, 1H), 3.14 (t, J = 9.3 Hz, 1H), 3.04 (q, J = 8.6 Hz, 1H), 2.63 (p, J = 7.0 Hz, 1H), 2.46 (q, J = 4.5 Hz, 1H), 2.16 (td, J = 13.4, 11.7, 5.6 Hz, 2H), 1.94 (dt, J = 12.9, 6.4 Hz, 1H), 1.71 (dtd, J = 22.3, 9.9, 9.3, 4.2 Hz, 2H), 1.20 (s, 9H), 0.90 (s, 9H). ^{13}C NMR (126 MHz, DMSO-d_6) δ 178.10, 171.57, 171.27, 157.51, 157.46, 130.05, 121.63, 120.11, 116.10, 74.99, 58.35, 57.11, 52.64, 49.46, 38.41, 37.22, 35.40, 34.85, 34.66, 29.75, 27.49, 26.74. ESI-HRMS Calcd for $C_{29}H_{43}N_6O_5$ [M+H]$^+$: 555.3289, found 555.3293.

HPLC purity 95.4% (Rt = 10.788 min, Method B).

Tert-butyl (2S,4R)-2-(((S)-1-amino-1-oxo-3-((S)-2-oxopyrrolidin-3-yl)propan-2-yl)carbamoyl)-4-phenyl-1l4-pyrrolidine-1-carboxylate (S11b). Compound **S11b** was prepared according to the procedure of compound **S11a**, white solid, yield: 68%. ^{1}H NMR (500 MHz, DMSO-d_6) δ 8.24 (d, 8.2 Hz, 1H), 7.66 (d, *J* = 11.2 Hz, 1H), 7.32 (td, *J* = 15.1, 13.7, 6.2 Hz, 4H), 7.25 (dd, *J* = 15.0, 7.9 Hz, 2H), 7.07 (d, *J* = 13.8 Hz, 1H), 4.34-4.19 (m, 2H), 3.90 (dt, *J* = 20.5, 8.2 Hz, 1H), 3.39 (t, *J* = 6.1 Hz, 1H), 3.25 (t, *J* = 10.4 Hz, 1H), 3.21-3.03 (m, 2H), 2.68-2.52 (m, 1H), 2.43 (d, *J* = 9.3 Hz, 1H), 2.22 (dt, *J* = 28.4, 10.4 Hz, 1H), 2.05-1.90 (m, 1H), 1.82 (dt, *J* = 23.1, 11.7 Hz, 1H), 1.75-1.45 (m, 2H), 1.37 (s, 9H). ^{13}C NMR (126 MHz, DMSO-d_6) δ 179.12, 174.09, 172.11, 157.34, 154.96, 154.12, 129.99, 121.32, 115.98, 80.08, 75.69, 60.22, 59.98, 52.55, 51.30, 38.20, 35.42, 33.62, 28.51, 28.03. ESI-HRMS Calcd for $C_{23}H_{33}N_4O_5$ [M+H]$^+$: 445.2445, found 445.2446.

(2S,4R)-N-((S)-1-amino-1-oxo-3-((S)-2-oxopyrrolidin-3-yl)propan-2-yl)-1-((S)-2-(3-(tert-butyl)ureido)-3,3-dimethylbutanoyl)-4-phenylpyrrolidine-2-carboxamide (S12b). Compound **S12b** was prepared according to the procedure of compound **S12a**, white solid, yield: 86%. ^{1}H NMR (400 MHz, DMSO-d_6) δ 8.31 (d, *J* = 8.8 Hz, 1H), 7.59 (s, 1H), 7.32 (d, *J* = 6.9 Hz, 5H), 7.27 (d, *J* = 7.0 Hz, 1H), 7.07 (s, 1H), 6.03 (d, *J* = 9.4 Hz, 1H), 5.99 (s, 1H), 4.41 (t, *J* = 8.6 Hz, 1H), 4.31 (q, *J* = 9.4, 8.3 Hz, 3H), 3.54-3.44 (m, 2H), 3.19-3.12 (m, 1H), 3.05 (d, *J* = 8.6 Hz, 1H), 2.57 (dd, *J* = 16.3, 7.8 Hz, 2H), 2.22 (dt, *J* = 14.5, 8.2 Hz, 1H), 1.98 (t, *J* = 13.2 Hz, 1H), 1.89 (d, *J* = 11.1 Hz, 1H), 1.65 (q, *J* = 10.3 Hz, 1H), 1.52 (t, *J* = 12.4 Hz, 1H), 1.20 (s, 9H), 0.92 (s, 9H). ^{13}C NMR (101 MHz, DMSO-d_6) δ 179.43, 174.09, 171.83, 171.11, 157.52, 140.19, 129.01, 127.65, 127.36, 60.74, 57.09, 54.91, 50.87, 49.42, 43.50, 37.78, 36.72, 35.66, 34.72, 29.76, 28.09, 26.77. ESI-HRMS Calcd for $C_{29}H_{45}N_6O_5$ [M+H]$^+$: 557.3446, found 557.3444.

(2S,4R)-1-((S)-2-(3-(tert-butyl)ureido)-3,3-dimethylbutanoyl)-N-((S)-1-cyano-2-((S)-2-oxopyrrolidin-3-yl)ethyl)-4-phenylpyrrolidine-2-carboxamide (5). Compound **5** was prepared according to the procedure of compound **2**, white solid, yield: 32%. ^{1}H NMR (500 MHz, DMSO-d_6) δ 9.00 (d, *J* = 8.7 Hz, 1H), 7.64 (s, 1H), 7.35 (d, *J* = 5.7 Hz, 4H), 7.27 (td, *J* = 5.9, 3.2 Hz, 1H), 6.00 (d, *J* = 9.5 Hz, 1H), 5.98 (s, 1H), 4.99 (ddd, *J* = 11.4, 8.7, 4.6 Hz, 1H), 4.37-4.23 (m, 3H), 3.45 (dd, *J* = 6.4, 3.6 Hz, 2H), 3.15 (t, *J* = 9.1 Hz, 1H), 3.04 (td, *J* = 9.3, 7.1 Hz, 1H), 2.56 (td, *J* = 10.9, 10.2, 4.6 Hz, 2H), 2.25-2.07 (m, 2H), 1.89 (q, *J* = 11.1 Hz, 1H), 1.79-1.63 (m, 2H), 1.20 (s, 9H), 0.91 (s, 9H). ^{13}C NMR (126 MHz, DMSO-d_6) δ 178.17, 171.96, 171.22, 157.53, 140.00, 129.04, 127.68, 127.43, 120.25, 60.48, 57.12, 54.78, 49.44, 43.51, 38.02, 37.06, 36.58, 35.59, 34.92, 29.77, 27.41, 26.74. ESI-HRMS Calcd for $C_{29}H_{43}N_6O_4$ [M+H]$^+$: 539.3340, found 539.3352. HPLC purity 95.1% (Rt = 11.808 min, Method B).

Methyl (2S,4S)-1-((S)-2-(3-(tert-butyl)ureido)-3,3-dimethylbutanoyl)-4-(phenylthio)pyrrolidine-2-carboxylate (S14a). Compound **S14a** was prepared according to the procedure of compound **S3**, white solid, yield: 97%. ^{1}H NMR (400 MHz, DMSO-d_6) δ 7.42 (d, J = 7.6 Hz, 2H), 7.36 (t, J = 7.3 Hz, 2H), 7.31-7.24 (m, 1H), 5.99 (d, J = 10.0 Hz, 2H), 4.41 (dt, J = 17.5, 8.2 Hz, 2H), 4.21 (d, J = 9.3 Hz, 1H), 3.96 (t, J = 7.6 Hz, 1H), 3.61 (d, J = 2.1 Hz, 3H), 3.41 (t, J = 9.4 Hz, 1H), 2.62 (d, J = 7.3 Hz, 1H), 1.79 (dd, J = 13.1, 7.6 Hz, 1H), 1.19 (s, 9H), 0.91 (s, 9H). ^{13}C NMR (101 MHz, DMSO-d_6) δ 171.85, 171.45, 157.44, 134.45, 130.62, 129.70, 127.40, 58.27, 56.97, 53.60, 52.23, 49.47, 43.20, 35.56, 35.34, 29.72, 26.61. ESI-HRMS Calcd for $C_{23}H_{36}N_3O_4S$ [M+H]$^+$: 450.2421, found 450.2421.

(2S)-1-((S)-2-(3-(tert-butyl)ureido)-3,3-dimethylbutanoyl)-4-(phenylthio)pyrrolidine-2-carboxylic acid (S15a). Compound **S15a** was prepared according to the procedure of compound **S4**, white solid, yield: 93%. ^{1}H NMR (400 MHz, DMSO-d_6) δ 12.57 (s, 1H), 7.43 (d, J = 7.7 Hz, 2H), 7.35 (t, J = 7.5 Hz, 2H), 7.28 (d, J = 7.3 Hz, 1H), 6.00 (d, J = 11.1 Hz, 2H), 4.39 (t, J = 8.9 Hz, 1H), 4.30 (t, J = 8.1 Hz, 1H), 4.21 (d, J = 9.3 Hz, 1H), 3.91 (t, J = 8.0 Hz, 1H), 3.39 (s, 1H), 2.67 (dd, J = 12.0, 5.8 Hz, 1H), 1.76 (q, J = 10.0 Hz, 1H), 1.20 (s, 9H), 0.91 (s, 9H). ^{13}C NMR (101 MHz, DMSO-d_6) δ 172.97, 171.24, 157.46, 134.52, 130.54, 129.69, 127.32, 58.55, 56.96, 53.78, 49.46, 43.00, 35.76, 35.47, 29.74, 26.67. ESI-HRMS Calcd for $C_{22}H_{33}N_3NaO_4S$ [M+Na]$^+$: 458.2084, found 458.2084.

(2S,4S)-N-((S)-1-amino-1-oxo-3-((S)-2-oxopyrrolidin-3-yl)propan-2-yl)-1-((S)-2-(3-(tert-butyl)ureido)-3,3-dimethylbutanoyl)-4-(phenylthio)pyrrolidine-2-carboxamide (S16a). Compound **S16a** was prepared according to the procedure of compound **S3**, white solid, yield: 98%. ^{1}H NMR (500 MHz, DMSO-d_6) δ 8.21 (d, J = 9.0 Hz, 1H), 7.55 (s, 1H), 7.47 (d, J = 1.5 Hz, 1H), 7.46 (t, J = 1.2 Hz, 1H), 7.36 (t, J = 7.6 Hz, 2H), 7.33 (d, J = 2.1 Hz, 1H), 7.31-7.26 (m, 1H), 7.05 (d, J = 2.0 Hz, 1H), 6.02 (d, J = 9.4 Hz, 1H), 5.96 (s, 1H), 4.36 (td, J = 9.6, 7.3 Hz, 2H), 4.28 (ddd, J = 12.3, 8.9, 3.4 Hz, 1H), 4.18 (d, J = 9.4 Hz, 1H), 3.87 (tt, J = 10.8, 6.8 Hz, 1H), 3.13 (t, J = 9.1 Hz, 1H), 3.02 (td, J = 9.3, 7.1 Hz, 1H), 2.61-2.51 (m, 2H), 2.22-2.14 (m, 1H), 1.98-1.91 (m, 1H), 1.74-1.61 (m, 2H), 1.50-1.44 (m, 1H), 1.20 (s, 10H), 0.89 (s, 9H). ^{13}C NMR (126 MHz, DMSO-d_6) δ 179.26, 173.95, 171.12, 162.78, 157.54, 134.47, 130.73, 129.69, 127.37, 59.90, 57.16, 54.21, 50.80, 49.45, 42.85, 38.72, 37.69, 36.31, 35.48, 34.79, 29.74, 28.00, 26.72. ESI-HRMS Calcd for $C_{29}H_{45}N_6O_5S$ [M+H]$^+$:589.3167, found 589.3166.

(2S,4S)-1-((S)-2-(3-(tert-butyl)ureido)-3,3-dimethylbutanoyl)-N-((S)-1-cyano-2-((S)-2-oxopyrrolidin-3-yl)ethyl)-4-(phenylthio)pyrrolidine-2-carboxamide (6). Compound **6** was prepared according to the procedure of compound **2**, white solid, yield: 46%. ^{1}H NMR (500 MHz, DMSO-d_6) δ 8.98 (d, J = 8.8 Hz, 1H), 7.66 (s, 1H), 7.48 (d, J = 7.4 Hz, 2H), 7.37 (t, J = 7.6 Hz, 2H), 7.30 (d, J = 7.2 Hz, 1H), 6.00 (d, J = 9.4 Hz, 1H),

5.95 (s, 1H), 4.96 (ddd, *J* = 11.5, 8.7, 4.6 Hz, 1H), 4.38 (dd, *J* = 10.0, 7.3 Hz, 1H), 4.23 (dd, *J* = 9.6, 7.3 Hz, 1H), 4.17 (d, *J* = 9.4 Hz, 1H), 3.92 (td, *J* = 8.9, 7.1, 3.9 Hz, 1H), 3.37 (d, *J* = 10.1 Hz, 1H), 3.14 (t, *J* = 9.1 Hz, 1H), 3.03 (dd, *J* = 9.3, 7.2 Hz, 1H), 2.55 (dd, *J* = 12.9, 6.8 Hz, 1H), 2.18 (ddd, *J* = 13.4, 11.3, 4.1 Hz, 1H), 2.09 (dt, *J* = 14.8, 7.7 Hz, 1H), 1.75-1.65 (m, 3H), 1.23 (d, *J* = 14.5 Hz, 1H), 1.20 (s, 9H), 0.88 (s, 9H). ^{13}C NMR (126 MHz, DMSO-d_6) δ 178.10, 171.27, 171.17, 157.53, 134.26, 130.93, 129.70, 127.49, 120.15, 59.67, 57.15, 54.10, 49.46, 42.84, 38.09, 37.05, 36.31, 35.43, 34.84, 29.74, 27.38, 26.67. ESI-HRMS Calcd for $C_{29}H_{43}N_6O_4S$ [M+H]$^+$: 571.3061, found 571.3061. HPLC purity 98.5% (Rt = 13.916 min, Method B).

Methyl (S)-7-((S)-2-(3-(tert-butyl)ureido)-3,3-dimethylbutanoyl)-1,4-dithia-7-azaspiro[4.4]nonane-8-carboxylate （S14b）. Compound **S1** (770 mg, 3.3 mmol) and compound **S13b** (1 g, 3.3 mmol) were dissolved in DCM/DMF (V:V=1:1, 20 mL) under N_2 atmosphere. Then NMM (860 mg, 8.5 mmol), BOP (1.77 g, 4.0 mmol) were added to the reaction at 25 °C and stirred for 12 h. Then DCM (20 mL) and 1N HCl aqueous solution (5 mL) were added to the reaction. After separation, the organic phase was washed with water (5 mL), the organic phase was washed with saturated brine (5 mL), dried over anhydrous sodium sulphate, evaporated in vacuum and purified by column chromatography to give the compound **S14b** as white solid (945 mg, yield: 66%). ^{1}H NMR (400 MHz, DMSO-d_6) δ 5.97 (d, *J* = 11.1 Hz, 1H), 4.41-4.27 (m, 2H), 4.25-4.18 (m, 1H), 3.91 (d, *J* = 10.8 Hz, 1H), 3.62 (s, 3H), 3.37 (tt, *J* = 8.2, 4.3 Hz, 4H), 2.70 (dd, *J* = 13.4, 7.9 Hz, 1H), 2.38 (dd, *J* = 13.2, 8.4 Hz, 1H), 1.19 (s, 9H), 0.92 (s, 9H). ^{13}C NMR (101 MHz, DMSO-d_6) δ 171.55, 171.30, 157.31, 67.44, 61.65, 58.92, 56.78, 52.34, 49.44, 44.53, 39.12, 35.34, 29.70, 26.58. ESI-HRMS Calcd for $C_{19}H_{33}N_3NaO_4S_2$ [M+Na]$^+$: 454.1805, found 454.1804.

(S)-7-((S)-2-(3-(tert-butyl)ureido)-3,3-dimethylbutanoyl)-1,4-dithia-7-azaspiro[4.4]-nonane-8-carboxylic acid (S15b). Compound **S15b** was prepared according to the procedure of compound **S4**, white solid, yield: 95%. ^{1}H NMR (400 MHz, DMSO-d_6) δ 12.58 (s, 1H), 6.01-5.93 (m, 2H), 4.33-4.19 (m, 3H), 3.87 (d, *J* = 10.9 Hz, 1H), 3.37 (td, *J* = 5.5, 4.8, 2.1 Hz, 3H), 2.68 (ddd, *J* = 13.2, 7.7, 1.3 Hz, 1H), 2.50 (p, *J* = 1.9 Hz, 1H), 2.34 (dd, *J* = 13.1, 8.8 Hz, 1H), 1.19 (s, 9H), 0.91 (s, 9H). ^{13}C NMR (101 MHz, DMSO-d_6) δ 172.32, 171.43, 157.37, 67.50, 61.77, 59.16, 56.82, 49.48, 44.69, 39.07, 35.46, 29.71, 26.64. ESI-HRMS Calcd for $C_{18}H_{31}N_3NaO_4S_2$ [M+Na]$^+$: 440.1648, found 440.1646.

(S)-N-((S)-1-amino-1-oxo-3-((S)-2-oxopyrrolidin-3-yl)propan-2-yl)-7-((S)-2-(3-(tert-butyl)ureido)-3,3-dimethylbutanoyl)-1,4-dithia-7-azaspiro[4.4]nonane-8-carboxamide (S16b). Compound **S16b** was prepared according to the procedure of compound **S3**, white solid, yield: 60%. ^{1}H NMR (400 MHz, DMSO-d_6) δ 8.29 (d, *J* =

8.8 Hz, 1H), 7.53 (s, 1H), 7.25 (s, 1H), 7.01 (s, 1H), 6.02-5.88 (m, 2H), 4.45-4.07 (m, 4H), 3.82 (d, *J* = 10.7 Hz, 1H), 3.37 (t, *J* = 7.2 Hz, 5H), 3.13 (t, *J* = 9.1 Hz, 1H), 3.04 (t, *J* = 8.5 Hz, 1H), 2.61-2.53 (m, 1H), 2.33-2.23 (m, 1H), 2.17 (p, *J* = 7.7 Hz, 1H), 2.00-1.86 (m, 1H), 1.62 (p, *J* = 9.8 Hz, 1H), 1.50 (t, *J* = 11.8 Hz, 1H), 1.19 (s, 9H), 0.89 (s, 9H). ^{13}C NMR (101 MHz, DMSO-*d*$_6$) δ 179.26, 173.90, 171.30, 170.66, 157.46, 67.54, 62.25, 60.57, 57.03, 50.93, 49.47, 45.01, 39.02, 37.72, 35.45, 34.57, 29.70, 27.98, 26.70. ESI-HRMS Calcd for $C_{25}H_{43}N_6O_5S_2$ [M+H]$^+$: 571.2731, found 571.2731.

(S)-7-((S)-2-(3-(tert-butyl)ureido)-3,3-dimethylbutanoyl)-N-((S)-1-cyano-2-((S)-2-oxopyrrolidin-3-yl)ethyl)-1,4-dithia-7-azaspiro[4.4]nonane-8-carboxamide (7). Compound **7** was prepared according to the procedure of compound **2**, white solid, yield: 30%. ^{1}H NMR (400 MHz, DMSO-*d*$_6$) ^{1}H NMR (400 MHz, DMSO-*d*$_6$) δ 8.99 (d, *J* = 8.6 Hz, 1H), 7.64 (s, 1H), 6.01-5.89 (m, 2H), 4.95 (s, 1H), 4.38-4.21 (m, 2H), 4.17 (d, *J* = 9.6 Hz, 1H), 3.87 (d, *J* = 10.7 Hz, 1H), 3.38 (s, 5H), 3.18-3.01 (m, 2H), 2.58 (d, *J* = 11.4 Hz, 1H), 2.28 (t, *J* = 11.4 Hz, 1H), 2.15 (d, *J* = 14.4 Hz, 2H), 1.68 (d, *J* = 13.1 Hz, 2H), 1.19 (s, 9H), 0.88 (s, 9H). ^{13}C NMR (101 MHz, DMSO-*d*$_6$) δ 178.09, 171.45, 170.73, 157.49, 120.07, 67.55, 61.75, 60.32, 57.07, 49.49, 45.24, 39.03, 38.18, 37.09, 35.33, 34.77, 29.69, 27.38, 26.66. ESI-HRMS Calcd for $C_{25}H_{41}N_6O_4S_2$ [M+H]$^+$: 553.2625, found 553.2629. HPLC purity 99.6% (Rt = 9.579 min, Method B.

Ethyl (1S,3aR,6aS)-2-((S)-2-(3-(tert-butyl)ureido)-3,3-dimethylbutanoyl)-octahydro-cyclopenta[c]pyrrole-1-carboxylate (S14c). Compound **S14c** was prepared according to the procedure of compound **S3**, white solid, yield: 88%. ^{1}H NMR (500 MHz, DMSO-*d*$_6$) δ 5.98 (s, 1H), 5.91 (d, *J* = 9.8 Hz, 1H), 4.26 (d, *J* = 9.9 Hz, 1H), 4.12 (d, *J* = 4.3 Hz, 1H), 4.07 (dtt, *J* = 15.0, 7.1, 3.6 Hz, 2H), 3.82 (dd, *J* = 10.6, 3.4 Hz, 1H), 3.71 (dd, *J* = 10.5, 7.5 Hz, 1H), 2.71-2.63 (m, 1H), 2.57 (dq, *J* = 7.8, 4.0 Hz, 1H), 1.89-1.80 (m, 1H), 1.80-1.72 (m, 1H), 1.67-1.58 (m, 1H), 1.58-1.41 (m, 3H), 1.19 (s, 9H), 1.17 (d, *J* = 2.1 Hz, 3H), 0.91 (s, 9H). ^{13}C NMR (101 MHz, DMSO-*d*$_6$) δ 171.65, 171.23, 157.21, 64.55, 60.36, 56.26, 53.13, 49.00, 46.84, 42.62, 34.43, 32.20, 31.25, 29.16, 26.29, 24.64, 14.04. ESI-HRMS Calcd for $C_{21}H_{38}N_3O_4$ [M+H]$^+$: 396.2857, found 396.2860.

(1S,3aR,6aS)-2-((S)-2-(3-(tert-butyl)ureido)-3,3-dimethylbutanoyl)octahydrocyclopenta[c]pyrrole-1-carboxylic acid (S15c). Compound **S15c** was prepared according to the procedure of compound **S4**, white solid, yield: 99%. ^{1}H NMR (500 MHz, DMSO-*d*$_6$) δ 12.41 (s, 1H), 5.99 (s, 1H), 5.91 (d, *J* = 9.9 Hz, 1H), 4.26 (d, *J* = 9.8 Hz, 1H), 4.08 (d, *J* = 4.1 Hz, 1H), 3.80 (dd, *J* = 10.7, 3.2 Hz, 1H), 3.70 (dd, *J* = 10.5, 7.5 Hz, 1H), 2.71-2.63 (m, 1H), 2.59 (dq, *J* = 7.9, 4.0 Hz, 1H), 1.85 (dt, *J* = 12.6, 7.6 Hz, 1H), 1.77 (dt, *J* = 12.8, 7.8 Hz, 1H), 1.62 (dt, *J* = 12.4, 7.0 Hz, 1H), 1.52 (ddd, *J* = 18.0, 8.3, 5.3 Hz, 2H), 1.44 (td, *J* = 13.2, 12.6, 6.1 Hz, 1H), 1.20 (s, 9H), 0.92 (s, 9H). ^{13}C NMR (101 MHz, DMSO-*d*$_6$) δ 173.46, 171.26, 157.43,

64.70, 56.38, 53.32, 49.17, 47.03, 42.70, 34.68, 32.60, 31.51, 29.30, 26.45, 24.83. ESI-HRMS Calcd for $C_{19}H_{34}N_3O_4$ [M+H]$^+$: 368.2544, found 368.2546.

(1S,3aR,6aS)-N-((S)-1-amino-1-oxo-3-((S)-2-oxopyrrolidin-3-yl)propan-2-yl)-2-((S)-2-(3-(tert-butyl)ureido)-3,3-dimethylbutanoyl)octahydrocyclopenta[c]pyrrole-1-carboxamide (S16c). Compound **S16c** was prepared according to the procedure of compound **S3**, white solid, yield: 73%. ^{1}H NMR (400 MHz, DMSO-*d*$_6$) δ 8.13 (d, *J* = 8.8 Hz, 1H), 7.55 (s, 1H), 7.24 (s, 1H), 7.03 (s, 1H), 5.95 (s, 1H), 5.90 (d, *J* = 9.8 Hz, 1H), 4.23 (t, *J* = 11.8 Hz, 2H), 4.11 (d, *J* = 4.6 Hz, 1H), 3.74 (t, *J* = 5.5 Hz, 2H), 3.12 (t, *J* = 9.1 Hz, 1H), 3.04 (d, *J* = 8.7 Hz, 1H), 2.88 (s, 2H), 2.72 (s, 2H), 2.14 (dt, *J* = 14.6, 8.0 Hz, 1H), 1.95 (t, *J* = 12.9 Hz, 1H), 1.75 (d, *J* = 7.0 Hz, 2H), 1.62 (s, 3H), 1.48-1.36 (m, 2H), 1.18 (s, 9H), 0.89 (s, 9H). ^{13}C NMR (101 MHz, DMSO-*d*$_6$) δ 178.82, 173.68, 171.82, 162.39, 157.35, 65.52, 56.48, 53.72, 50.42, 49.03, 47.30, 42.85, 38.31, 37.38, 35.86, 34.61, 31.53, 31.12, 29.23, 27.55, 26.44, 24.53. ESI-HRMS Calcd for $C_{26}H_{45}N_6O_5$ [M+H]$^+$: 521.3446, found 521.3455.

(1S,3aR,6aS)-2-((S)-2-(3-(tert-butyl)ureido)-3,3-dimethylbutanoyl)-N-((S)-1-cyano-2-((S)-2-oxopyrrolidin-3-yl)ethyl)octahydrocyclopenta[c]pyrrole-1-carboxamide (8). Compound **8** was prepared according to the procedure of compound **2**, white solid, yield: 41%. ^{1}H NMR (400 MHz, DMSO-*d*$_6$) δ 8.87 (d, *J* = 8.6 Hz, 1H), 7.64 (s, 1H), 5.94 (s, 1H), 5.88 (d, *J* = 9.7 Hz, 1H), 4.94 (td, *J* = 10.4, 9.2, 5.1 Hz, 1H), 4.20 (d, *J* = 9.7 Hz, 1H), 3.99 (d, *J* = 4.8 Hz, 1H), 3.77 (d, *J* = 5.0 Hz, 2H), 3.14 (t, *J* = 9.2 Hz, 1H), 3.03 (d, *J* = 8.6 Hz, 1H), 2.77-2.60 (m, 1H), 2.49-2.43 (m, 2H), 2.17 (ddd, *J* = 19.2, 10.8, 5.3 Hz, 1H), 2.08 (q, *J* = 6.5, 5.2 Hz, 1H), 1.80 (q, *J* = 6.4 Hz, 2H), 1.66 (dtd, *J* = 23.1, 15.0, 6.7 Hz, 5H), 1.45-1.37 (m, 1H), 1.18 (d, *J* = 2.0 Hz, 9H), 0.89 (s, 9H). ^{13}C NMR (126 MHz, DMSO-*d*$_6$) δ 178.08, 172.36, 171.59, 157.78, 120.26, 65.79, 56.95, 54.09, 49.45, 47.85, 43.47, 38.04, 37.10, 34.92, 34.70, 31.85, 31.42, 29.64, 27.35, 26.81, 24.93. ESI-HRMS Calcd for $C_{26}H_{43}N_6O_4$ [M+H]$^+$: 503.3340, found 503.3352. HPLC purity 99.5% (Rt = 8.844 min, Method B).

Methyl (S)-7-((S)-2-((tert-butoxycarbonyl)amino)-3,3-dimethylbutanoyl)-1,4-dithia-7-azaspiro[4.4]nonane-8-carboxylate (S18). Compound **S18** was prepared according to the procedure of compound **S14b**, white solid, yield: 60%. ^{1}H NMR (400 MHz, DMSO-*d*$_6$) δ 6.71 (d, *J* = 9.3 Hz, 1H), 4.39 (t, *J* = 8.2 Hz, 1H), 4.25 (d, *J* = 10.9 Hz, 1H), 4.15-4.06 (m, 1H), 3.93 (t, *J* = 10.6 Hz, 1H), 3.63 (s, 3H), 3.38 (dd, *J* = 6.1, 3.3 Hz, 4H), 2.70 (dt, *J* = 12.2, 6.0 Hz, 1H), 2.38 (dd, *J* = 13.2, 8.4 Hz, 1H), 1.38 (s, 9H), 0.95 (s, 9H). ^{13}C NMR (101 MHz, DMSO-*d*$_6$) δ 171.27, 170.52, 156.10, 78.72, 67.48, 61.71, 59.02, 58.86, 52.37, 44.46, 39.15, 35.20, 28.63, 26.60. ESI-HRMS Calcd for $C_{19}H_{33}N_2O_5S_2$ [M+H]$^+$: 433.1825, found 433.1833.

(S)-7-((S)-2-((tert-butoxycarbonyl)amino)-3,3-dimethylbutanoyl)-1,4-dithia-7-azaspiro[4.4]nonane-8-carboxylic acid (S19). Compound **S19** was prepared according to the procedure of compound **S4**, white solid, yield: 83%. ^{1}H NMR (400 MHz, DMSO-d_6) δ 12.61 (s, 1H), 6.67 (d, *J* = 9.4 Hz, 1H), 4.33-4.18 (m, 2H), 4.11 (d, *J* = 9.4 Hz, 1H), 3.88 (d, *J* = 10.9 Hz, 1H), 3.37-3.28 (m, 4H), 2.69 (dd, *J* = 13.1, 7.9 Hz, 1H), 2.34 (dd, *J* = 13.1, 8.9 Hz, 1H), 1.37 (s, 9H), 0.94 (s, 9H). ^{13}C NMR (101 MHz, DMSO-d_6) δ 172.30, 170.37, 156.07, 78.68, 67.55, 61.84, 59.25, 58.82, 44.63, 39.09, 35.30, 28.63, 26.64. ESI-HRMS Calcd for $C_{18}H_{31}N_2O_5S_2$ [M+H]$^+$: 419.1669, found 419.1669.

***Tert-butyl ((S)-1-((S)-8-(((S)-1-amino-1-oxo-3-((S)-2-oxopyrrolidin-3-yl)propan-2-yl)carbamoyl)-1,4-dithia-7-azaspiro[4.4]nonan-7-yl)-3,3-dimethyl-1-oxobutan-2-yl)carbamate* (*S20*).** Compound **S20** was prepared according to the procedure of compound **S3**, white solid, yield: 40%. ^{1}H NMR (400 MHz, DMSO-d_6) δ 8.30 (d, *J* = 8.7 Hz, 1H), 7.55 (s, 1H), 7.25 (s, 1H), 7.02 (s, 1H), 6.71 (d, *J* = 9.5 Hz, 1H), 4.40 (dd, *J* = 9.9, 7.1 Hz, 1H), 4.32-4.20 (m, 2H), 4.08 (d, *J* = 9.5 Hz, 1H), 3.83 (d, *J* = 10.7 Hz, 1H), 3.42-3.35 (m, 4H), 3.17-2.99 (m, 2H), 2.58 (dd, *J* = 13.0, 7.2 Hz, 1H), 2.48 (dd, *J* = 14.4, 9.4 Hz, 1H), 2.27 (dd, *J* = 13.0, 10.0 Hz, 1H), 2.16 (dt, *J* = 14.0, 7.6 Hz, 1H), 1.94 (td, *J* = 13.1, 3.7 Hz, 1H), 1.61 (dq, *J* = 11.8, 9.3 Hz, 1H), 1.49 (ddd, *J* = 14.4, 11.6, 3.6 Hz, 1H), 1.37 (s, 9H), 0.91 (s, 9H). ^{13}C NMR (101 MHz, DMSO-*d*6) δ 179.24, 173.93, 170.67, 170.27, 156.15, 78.64, 67.57, 62.28, 60.52, 58.92, 50.96, 44.96, 39.01, 37.77, 35.22, 34.46, 28.61, 27.97, 26.67. ESI-HRMS Calcd for $C_{25}H_{42}N_5O_6S_2$ [M+H]$^+$: 572.2571, found 572.2580.

(S)-N-((S)-1-cyano-2-((S)-2-oxopyrrolidin-3-yl)ethyl)-7-((S)-3,3-dimethyl-2-(2,2,2-trifluoroacetamido)butanoyl)-1,4-dithia-7-azaspiro[4.4]nonane-8-carboxamide (9). Compound **S20** (400 mg, 0.7 mmol) and 4 M HCl in 1,4-dioxane (2 mL) were dissolved in 1,4-dioxane (2 mL), the reaction was stirred at 25 °C for 2 h. Then the reaction was concentrated to remove the solvent and Et_3N (225 mg, 2.2 mmol), trifluoroacetic anhydride (165 mg, 0.8 mmol), DCM (4 mL) were added to the reaction at 0 °C. The reaction was allowed to warm to 25 °C and stirred for 10 h. Then DCM (10 mL) and 1N HCl aqueous solution (3 mL) were added to the reaction. After separation, the organic phase was washed with water (5 mL), the organic phase was washed with saturated brine (5 mL), dried over anhydrous sodium sulphate, evaporated in vacuum and purified by column chromatography to give **9** as white solid (154 mg, yield: 40%). ^{1}H NMR (400 MHz, DMSO-*d*6) δ 9.44 (d, *J* = 8.6 Hz, 1H), 9.04 (d, *J* = 8.5 Hz, 1H), 7.66 (s, 1H), 4.96 (dq, *J* = 8.9, 4.8 Hz, 1H), 4.57-4.47 (m, 1H), 4.33 (dd, *J* = 9.9, 7.1 Hz, 1H), 4.19 (d, *J* = 10.9 Hz, 1H), 3.91 (d, *J* = 10.9 Hz, 1H), 3.40 (tt, *J* = 13.0, 6.1 Hz, 4H), 3.14 (t, *J* = 9.3 Hz, 1H), 3.05 (q, *J* = 8.7 Hz, 1H), 2.61 (dd, *J* = 13.0, 7.1 Hz, 1H), 2.47 (d, *J* = 9.0 Hz, 1H), 2.30 (dd, *J* = 12.9, 10.0 Hz, 1H), 2.13 (ddt, *J* = 18.2, 13.5, 5.9

Hz, 2H), 1.78-1.65 (m, 2H), 0.98 (s, 9H). ^{13}C NMR (101 MHz, DMSO-d_6) δ 178.04, 170.51, 168.22, 157.04, 119.98, 117.75, 67.48, 62.43, 60.40, 58.45, 44.99, 39.18, 38.29, 37.18, 35.57, 34.77, 27.37, 26.55. ^{19}F NMR (376 MHz, DMSO-d_6) δ -72.93. ESI-HRMS Calcd for $C_{22}H_{31}F_3N_5O_4S_2$ $[M+H]^+$: 550.1764, found 550.1766. HPLC purity 99.9% (Rt = 11.336 min, Method D).

Methyl (S)-7-((S)-2-(1-fluorocyclopropane-1-carboxamido)-3,3-dimethylbutanoyl)-1,4-dithia-7-azaspiro[4.4]nonane-8-carboxylate (S21). Compound **S18** (540 mg, 1.2 mmol) and 4 M HCl in 1,4-dioxane (4 mL) were dissolved in 1,4-dioxane (4 mL), and stirred at 25 °C for 2 h. The reaction was concentrated to remove the solvent. Then 1-Fluoro-cyclopropanecarboxylic acid (130 mg, 1.2 mmol), HATU (712 mg, 1.9 mmol), DCM (8 mL) were added to the reaction and stirred at 25 °C for 1 h. Then DIPEA (340 mg, 2.6 mmol) was added to the reaction and stirred at 25 °C for 10 h. Then DCM (10 mL) and 1N HCl aqueous solution (5 mL) were added to the reaction. After separation, the organic phase was washed with water (5 mL), the organic phase was washed with saturated brine (5 mL), dried over anhydrous sodium sulphate, evaporated in vacuum and purified by column chromatography to give the compound **S21** as white solid (468 mg, yield: 90%). ^{1}H NMR (400 MHz, DMSO-d_6) δ 7.37-7.31 (m, 1H), 4.57 (d, J = 9.0 Hz, 1H), 4.47 (t, J = 8.0 Hz, 1H), 4.18 (d, J = 11.0 Hz, 1H), 3.95 (d, J = 10.9 Hz, 1H), 3.61 (d, J = 22.1 Hz, 3H), 3.37 (d, J = 5.9 Hz, 4H), 2.72 (dd, J = 13.3, 8.3 Hz, 1H), 2.42 (dd, J = 13.3, 7.8 Hz, 1H), 1.40-1.30 (m, 2H), 1.18 (td, J = 8.6, 4.0 Hz, 2H), 0.99 (s, 9H). ^{13}C NMR (101 MHz, DMSO-d_6) δ 171.11, 169.51, 168.95, 79.58, 67.38, 62.19, 59.06, 57.06, 52.46, 44.20, 39.32, 35.96, 26.50, 13.38. ESI-HRMS Calcd for $C_{18}H_{28}FN_2O_4S_2$ $[M+H]^+$: 419.1469, found 419.1472.

(S)-7-((S)-2-(1-fluorocyclopropane-1-carboxamido)-3,3-dimethylbutanoyl)-1,4-dithia-7-azaspiro[4.4]nonane-8-carboxylic acid (S22). Compound **S22** was prepared according to the procedure of compound **S4**, white solid, yield: 74%. ^{1}H NMR (400 MHz, DMSO-d_6) δ 12.74 (s, 1H), 7.33 (dd, J = 9.2, 2.8 Hz, 1H), 4.56 (d, J = 9.0 Hz, 1H), 4.35 (t, J = 8.2 Hz, 1H), 4.18 (d, J = 11.0 Hz, 1H), 3.92 (d, J = 11.0 Hz, 1H), 3.37 (dd, J = 5.6, 3.6 Hz, 4H), 2.71 (dd, J = 13.2, 8.2 Hz, 1H), 2.38 (dd, J = 13.2, 8.3 Hz, 1H), 1.42-1.29 (m, 2H), 1.21-1.14 (m, 2H), 0.99 (s, 9H). ^{13}C NMR (101 MHz, DMSO-d_6) δ 172.10, 169.36, 168.68, 79.60, 67.44, 62.36, 59.27, 57.03, 44.35, 39.28, 36.07, 26.54, 13.38. ESI-HRMS Calcd for $C_{17}H_{26}FN_2O_4S_2$ $[M+H]^+$: 405.1313, found 405.1320.

(S)-N-((S)-1-amino-1-oxo-3-((S)-2-oxopyrrolidin-3-yl)propan-2-yl)-7-((S)-2-(1-fluorocyclopropane-1-carboxamido)-3,3-dimethylbutanoyl)-1,4-dithia-7-azaspiro-[4.4]nonane-8-carboxamide (S23). Compound **S23** was prepared according to the procedure of compound **S3**, white solid, yield: 60%. ^{1}H NMR (400 MHz, DMSO-d_6) δ

8.32 (d, J = 8.7 Hz, 1H), 7.55 (s, 1H), 7.38 (dd, J = 9.2, 2.7 Hz, 1H), 7.31 (s, 1H), 7.05-6.97 (m, 1H), 4.60-4.42 (m, 2H), 4.33-4.15 (m, 2H), 3.83 (d, J = 10.9 Hz, 1H), 3.66-3.51 (m, J = 6.2, 5.7 Hz, 2H), 3.43-3.35 (m, 2H), 3.20-3.00 (m, 4H), 2.63-2.56 (m, 1H), 2.46-2.39 (m, 1H), 2.29 (dd, J = 13.1, 9.8 Hz, 1H), 2.22-2.12 (m, 1H), 2.01-1.90 (m, 1H), 1.72-1.40 (m, 2H), 1.38-1.12 (m, 2H), 0.96 (s, 9H). ^{13}C NMR (101 MHz, DMSO-d_6) δ 179.12, 173.84, 170.48, 169.10, 168.76, 79.51, 67.46, 62.91, 60.48, 57.13, 53.79, 44.72, 42.06, 39.23, 37.83, 36.00, 34.53, 27.94, 26.57, 13.38. ESI-HRMS Calcd for $C_{24}H_{37}FN_5O_5S_2$ $[M+H]^+$: 558.2215, found 558.2211.

(S)-N-((S)-1-cyano-2-((S)-2-oxopyrrolidin-3-yl)ethyl)-7-((S)-2-(1-fluorocyclopropane-1-carboxamido)-3,3-dimethylbutanoyl)-1,4-dithia-7-azaspiro[4.4]nonane-8-carboxamide (10). Compound **10** was prepared according to the procedure of compound **2**, white solid, yield: 41%. ^{1}H NMR (400 MHz, DMSO-d_6) δ 9.02 (d, J = 8.4 Hz, 1H), 7.67 (s, 1H), 7.40 (dd, J = 9.1, 2.6 Hz, 1H), 4.95 (ddd, J = 10.5, 6.7, 4.2 Hz, 1H), 4.53 (d, J = 9.0 Hz, 1H), 4.33 (dd, J = 9.8, 7.2 Hz, 1H), 4.21 (d, J = 11.0 Hz, 1H), 3.88 (d, J = 10.9 Hz, 1H), 3.46-3.33 (m, 4H), 3.21-3.10 (m, 1H), 3.06 (td, J = 9.3, 7.0 Hz, 1H), 2.67-2.56 (m, 1H), 2.49-2.39 (m, 1H), 2.30 (dd, J = 12.9, 9.8 Hz, 1H), 2.14 (ddt, J = 16.5, 14.3, 6.9 Hz, 2H), 1.71 (tdd, J = 14.9, 11.3, 7.1 Hz, 2H), 1.42-1.26 (m, 2H), 1.26-1.12 (m, 2H), 0.96 (s, 9H). ^{13}C NMR (101 MHz, DMSO-d_6) δ 177.99, 170.53, 169.27, 168.83, 119.97, 79.48, 67.47, 62.45, 60.31, 57.20, 44.91, 39.24, 38.43, 37.26, 35.87, 34.56, 27.42, 26.55, 13.38. ^{19}F NMR (376 MHz, DMSO-d_6) δ -75.06. ESI-HRMS Calcd for $C_{24}H_{35}FN_5O_4S_2$ $[M+H]^+$: 540.2109, found 540.2114. HPLC purity 98.9% (Rt = 8.212 min, Method B).

(S)-N-((S)-1-amino-1-oxo-3-((S)-2-oxopyrrolidin-3-yl)propan-2-yl)-7-((S)-3,3-dimethyl-2-(methylsulfonamido)butanoyl)-1,4-dithia-7-azaspiro[4.4]nonane-8-carboxamide (S24). Compound **S20** (400 mg, 0.8 mmol) and 4 M HCl in 1,4-dioxane (2 mL) were dissolved in DCM (10 mL), the reaction was stirred at 25 °C for 2 h. The reaction was concentrated to remove the solvent. Then Et_3N (258 mg, 2.6 mmol), methanesulfonyl chloride (115 mg, 1.0 mmol) and DCM (4 mL) were added to the reaction at 0 °C. The reaction was allowed to warm to 25 °C and stirred for 1 h. Then DCM (10 mL) and 1N HCl aqueous solution (3 mL) were added to the reaction. After separation, the organic phase was washed with water (5 mL), the organic phase was washed with saturated brine (5 mL), dried over anhydrous sodium sulphate, evaporated in vacuum and purified by column chromatography to give the compound **S24** as white solid (170 mg, yield: 44%). ^{1}H NMR (400 MHz, DMSO-d_6) δ 8.34 (d, J = 9.0 Hz, 1H), 7.53 (s, 1H), 7.30 (t, J = 5.1 Hz, 2H), 7.02 (s, 1H), 4.49 (dd, J = 9.9, 7.1 Hz, 1H), 4.29 (ddd, J = 12.3, 8.8, 2.9 Hz, 1H), 4.17 (d, J = 11.1 Hz, 1H), 3.92-3.75 (m, 2H), 3.49-3.41 (m, 1H), 3.35 (s, 3H), 3.09 (dt, J = 29.2, 8.9 Hz, 2H), 2.84 (s, 3H), 2.61 (dd, J = 12.8,

6.9 Hz, 1H), 2.53 (d, J = 2.1 Hz, 1H), 2.30 (dd, J = 12.8, 9.8 Hz, 1H), 2.18 (dd, J = 13.1, 7.1 Hz, 1H), 1.96 (td, J = 13.2, 3.5 Hz, 1H), 1.72-1.56 (m, 1H), 1.53-1.43 (m, 1H), 0.96 (s, 9H). ^{13}C NMR (101 MHz, DMSO-d_6) δ 179.19, 173.81, 170.52, 169.68, 67.62, 62.79, 61.36, 60.57, 55.38, 50.76, 44.55, 41.17, 39.00, 37.70, 35.79, 34.72, 27.89, 26.6. ESI-HRMS Calcd for $C_{21}H_{36}N_5O_6S_3$ [M+H]$^+$: 550.1822, found 550.1825.

(S)-N-((S)-1-cyano-2-((S)-2-oxopyrrolidin-3-yl)ethyl)-7-((S)-3,3-dimethyl-2-(methylsulfonamido)butanoyl)-1,4-dithia-7-azaspiro[4.4]nonane-8-carboxamide (11). Compound **11** was prepared according to the procedure of compound **2**, white solid, yield: 72%. ^{1}H NMR (400 MHz, DMSO-d_6) δ 9.07 (d, J = 8.7 Hz, 1H), 7.65 (s, 1H), 7.29 (d, J = 10.1 Hz, 1H), 4.97 (ddd, J = 11.1, 8.6, 4.8 Hz, 1H), 4.35 (dd, J = 10.1, 7.0 Hz, 1H), 4.19 (d, J = 11.0 Hz, 1H), 3.90-3.81 (m, 2H), 3.50-3.33 (m, 4H), 3.18-2.99 (m, 2H), 2.83 (s, 3H), 2.68-2.56 (m, 1H), 2.52 (d, J = 2.0 Hz, 1H), 2.36-2.25 (m, 1H), 2.13 (dddd, J = 27.5, 17.0, 10.1, 3.1 Hz, 2H), 1.70 (dddd, J = 11.7, 9.1, 7.1, 3.5 Hz, 2H), 0.94 (s, 9H). ^{13}C NMR (101 MHz, DMSO-d_6) δ 178.04, 170.57, 169.94, 120.01, 67.62, 62.31, 61.28, 60.44, 55.38, 44.75, 41.17, 39.04, 38.18, 37.13, 35.75, 34.80, 27.34, 26.57. ESI-HRMS Calcd for $C_{21}H_{34}N_5O_5S_3$ [M+H]$^+$: 532.1717, found 532.1728. HPLC purity 96.9% (Rt = 6.152 min, Method B).

(S)-N-((S)-1-amino-1-oxo-3-((S)-2-oxopyrrolidin-3-yl)propan-2-yl)-7-((S)-2-(cyclopropanesulfonamido)-3,3-dimethylbutanoyl)-1,4-dithia-7-azaspiro-[4.4]nonane-8-carboxamide (S25). Compound **S25** was prepared according to the procedure of compound **S24**, white solid, yield: 40%. ^{1}H NMR (400 MHz, DMSO-d_6) δ 8.31 (d, J = 9.0 Hz, 1H), 7.53 (s, 1H), 7.30 (s, 1H), 7.16 (d, J = 9.9 Hz, 1H), 7.01 (s, 1H), 4.48 (dd, J = 9.9, 7.1 Hz, 1H), 4.29 (ddd, J = 12.3, 9.0, 3.3 Hz, 1H), 4.18 (d, J = 11.0 Hz, 1H), 3.90-3.77 (m, 2H), 3.46-3.33 (m, 4H), 3.16-2.99 (m, 2H), 2.64-2.55 (m, 1H), 2.47 (d, J = 7.1 Hz, 1H), 2.36-2.25 (m, 1H), 2.23-2.11 (m, 1H), 1.95 (td, J = 13.1, 3.5 Hz, 1H), 1.63 (dq, J = 12.1, 9.2 Hz, 1H), 1.52-1.41 (m, 1H), 1.36-1.20 (m, 3H), 0.96 (s, 9H), 0.90 (dd, J = 6.8, 5.1 Hz, 1H), 0.82 (dt, J = 7.9, 3.2 Hz, 1H). ^{13}C NMR (101 MHz, DMSO-d_6) δ 179.23, 173.82, 170.64, 169.77, 67.58, 62.82, 61.35, 60.73, 55.38, 50.73, 44.78, 39.21, 37.70, 36.05, 34.81, 30.81, 27.93, 26.66, 5.77, 5.51. ESI-HRMS Calcd for $C_{23}H_{38}N_5O_6S_3$ [M+H]$^+$: 576.1979, found 576.1981.

(S)-N-((S)-1-cyano-2-((S)-2-oxopyrrolidin-3-yl)ethyl)-7-((S)-2-(cyclopropanesulfonamido)-3,3-dimethylbutanoyl)-1,4-dithia-7-azaspiro[4.4]nonane-8-carboxamide (12). Compound **12** was prepared according to the procedure of compound **2**, white solid, yield: 67%. ^{1}H NMR (400 MHz, DMSO-d_6) δ 9.06 (d, J = 8.7 Hz, 1H), 7.65 (s, 1H), 7.17 (d, J = 9.9 Hz, 1H), 4.97 (ddd, J = 11.1, 8.6, 4.9 Hz, 1H), 4.33 (dd, J = 10.1, 7.0 Hz, 1H), 4.19 (d, J = 11.1 Hz, 1H), 3.91-3.81 (m, 2H), 3.46-3.35 (m, 4H), 3.18-3.00 (m, 2H), 2.61 (dd, J = 13.0, 7.1 Hz, 1H), 2.48 (s, 1H), 2.30 (dd, J = 12.9, 10.0 Hz, 1H),

2.21-2.05 (m, 2H), 1.75-1.64 (m, 2H), 1.36-1.20 (m, 3H), 0.95 (s, 9H), 0.95-0.74 (m, 2H). ^{13}C NMR (101 MHz, DMSO-d_6) δ 178.07, 170.67, 170.00, 120.02, 67.59, 62.33, 61.32, 60.59, 55.38, 44.94, 39.23, 38.20, 37.14, 36.01, 34.83, 30.84, 27.36, 26.63, 5.72, 5.52. ESI-HRMS Calcd for $C_{23}H_{36}N_5O_5S_3$ $[M+H]^+$: 558.1873, found 558.1874. HPLC purity 95.2% (Rt = 7.664 min, Method B).

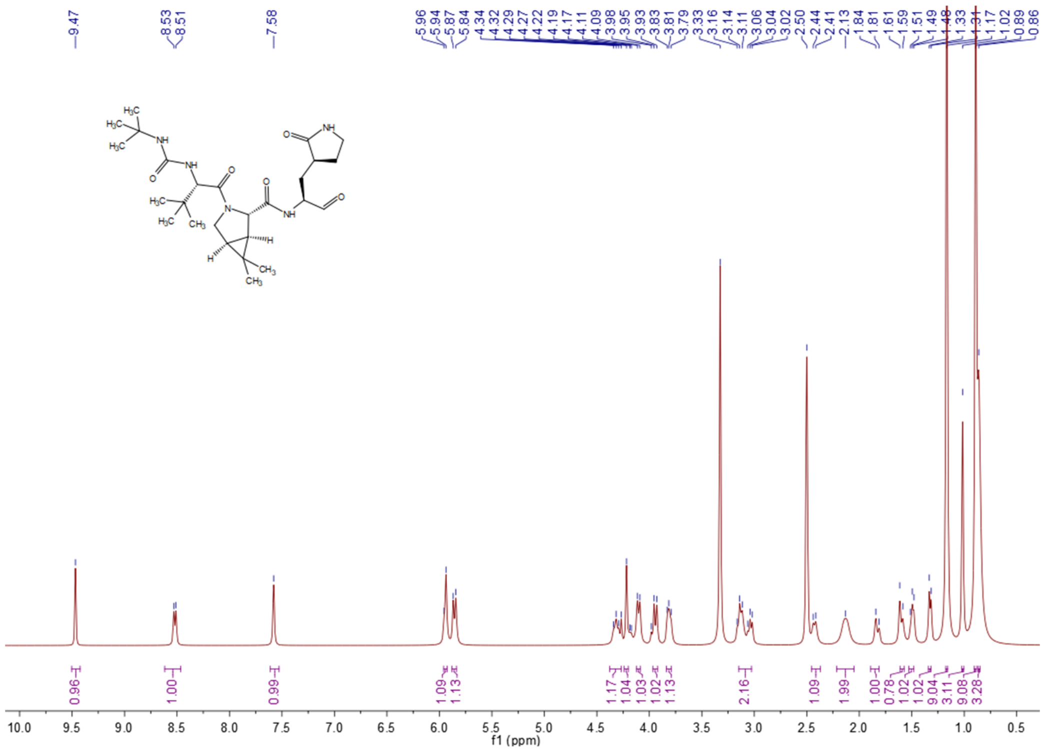

^{1}H NMR of **1**

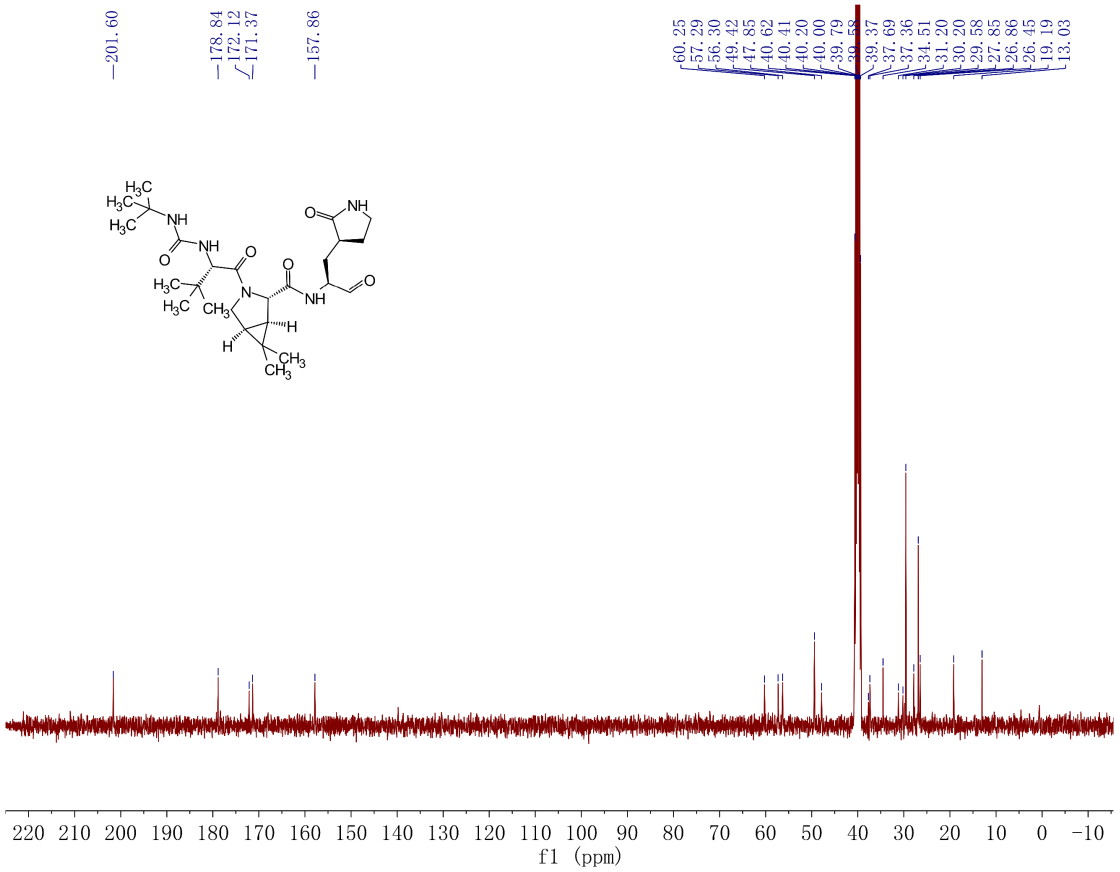

^{13}C NMR of **1**

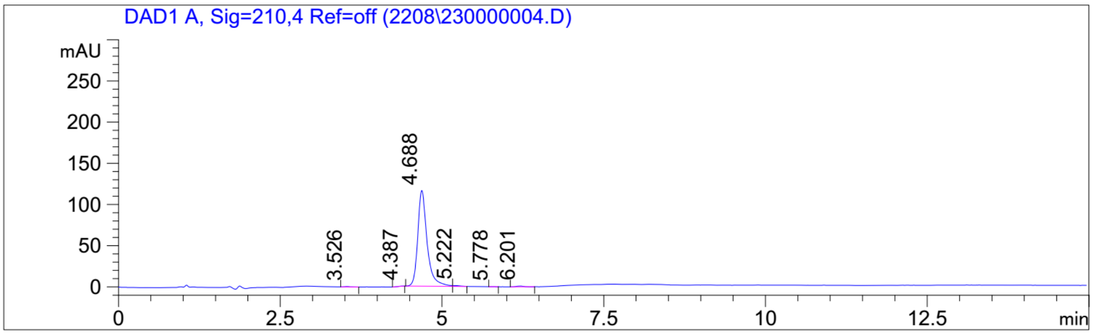

Peak #	RT [min]	Width [min]	Area [mAU*s]	Height [mAU]	Area %
1	3.526	0.0743	2.17273	0.42005	0.1840
2	4.387	0.0540	0.89901	0.29116	0.0761
3	4.688	0.1468	1163.26721	116.23634	98.4991
4	5.222	0.1090	8.26054	1.08246	0.6995
5	5.778	0.0264	0.08750	0.08251	0.0074
6	6.201	0.1160	6.30603	0.89754	0.5340

HPLC of **1**

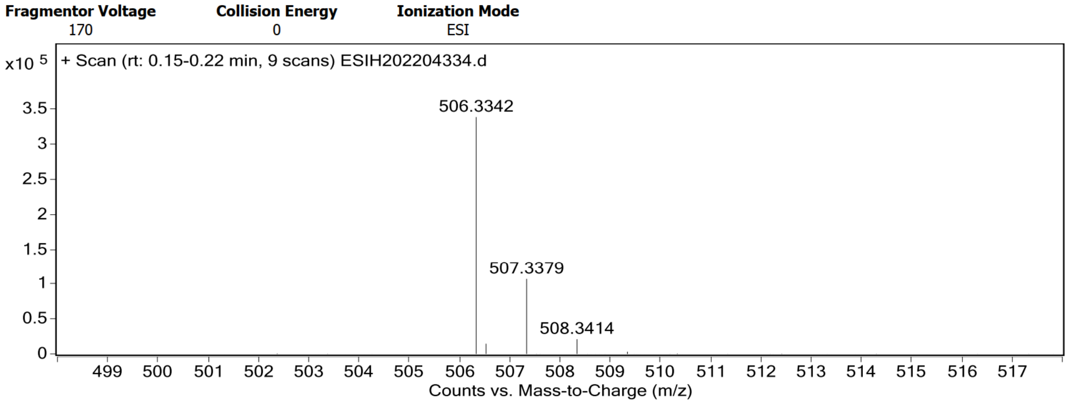

Formula Calculator Results

m/z	Calc m/z	Diff (mDa)	Diff (ppm)	Ion Formula	Ion
506.3342	506.3337	-0.53	-1.05	C26 H44 N5 O5	(M+H)+

--- End Of Report ---

HRMS of **1**

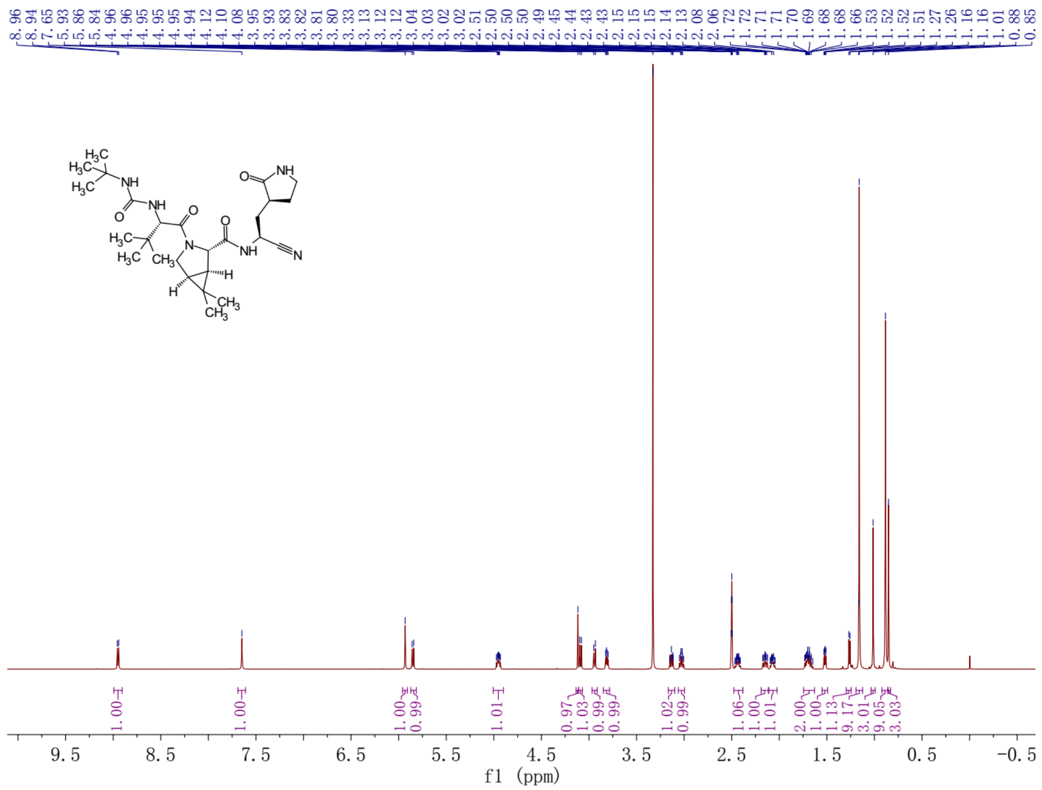

^{1}H NMR of **2**

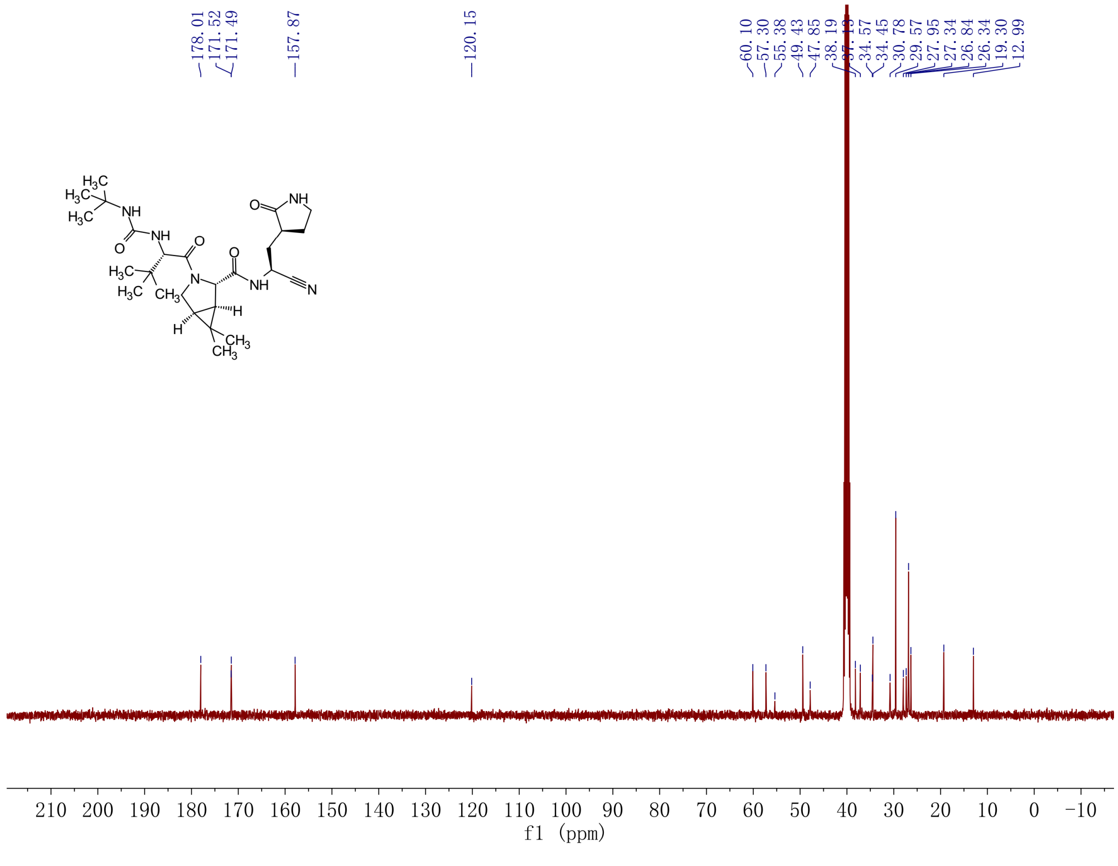

^{13}C NMR of **2**

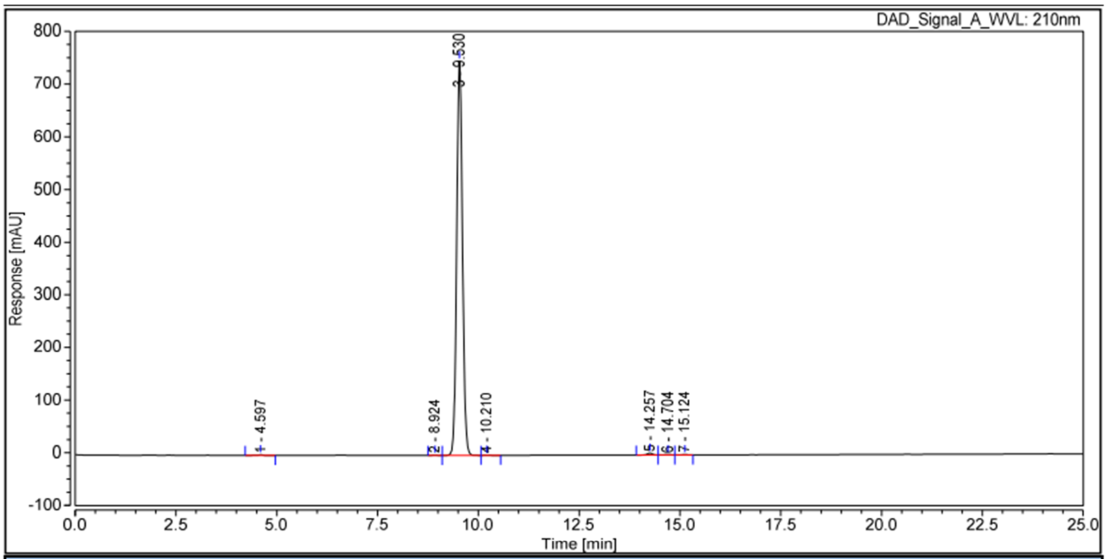

Integration Results

No.	Peak Name	Retention Time min	Area mAU*s	Height mAU	Relative Area %	S/N	Resolution (EP)
1		4.597	13.28421	1.361	0.1767	182.5	20.49
2		8.924	4.52881	0.531	0.0602	71.3	2.49
3		9.530	7449.77408	749.049	99.0860	100479.9	2.67
4		10.210	3.99713	0.399	0.0532	53.6	16.81
5		14.257	32.28688	3.614	0.4294	484.7	1.85
6		14.704	7.97347	0.764	0.1061	102.5	1.67
7		15.124	6.65165	0.674	0.0885	90.4	n.a.
**Total:**			**7518.496**	**756.391**	**100.0000**	**101464.89**	

HPLC of **2**

Fragmentor Voltage	Collision Energy	Ionization Mode
170	0	ESI

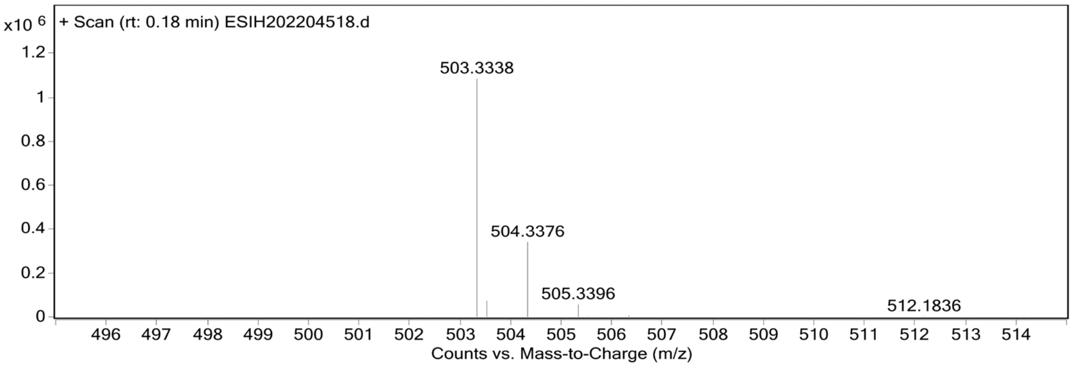

Formula Calculator Results

m/z	Calc m/z	Diff (mDa)	Diff (ppm)	Ion Formula	Ion
503.3338	503.334	0.25	0.5	C26 H43 N6 O4	(M+H)+

--- End Of Report ---

HRMS of **2**

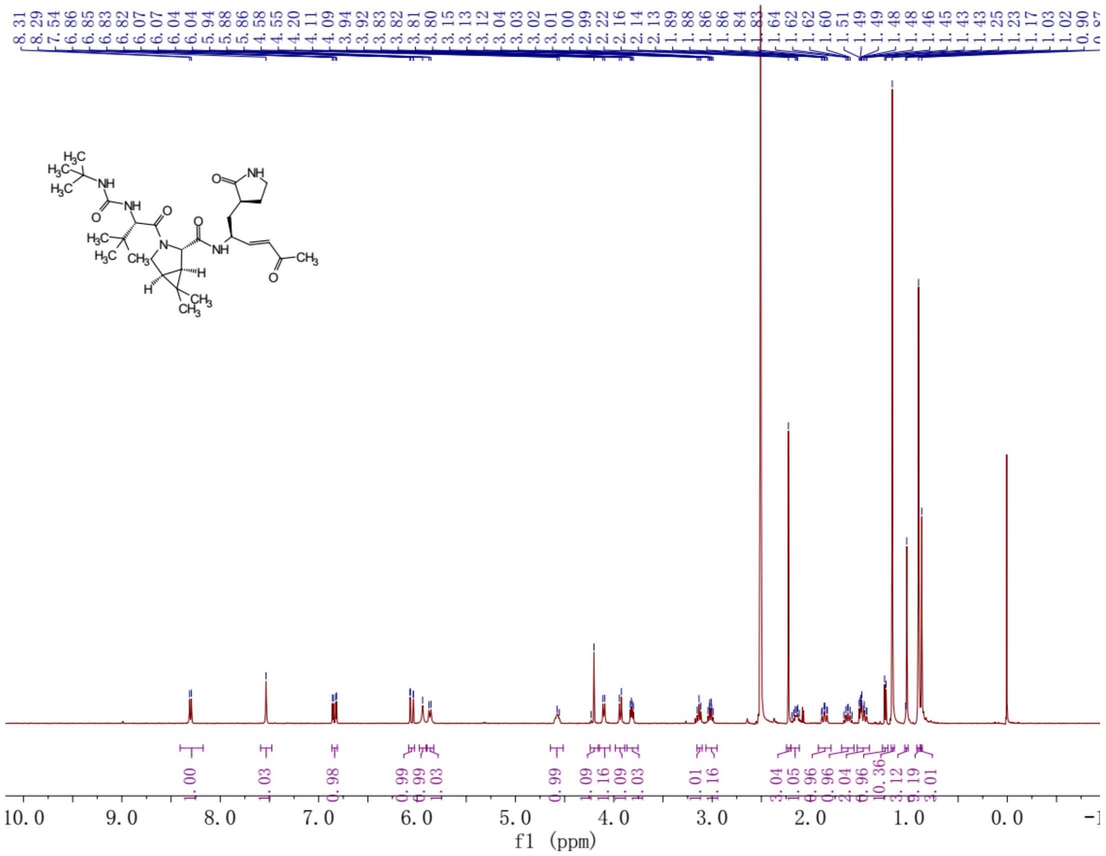

^{1}H NMR of **3**

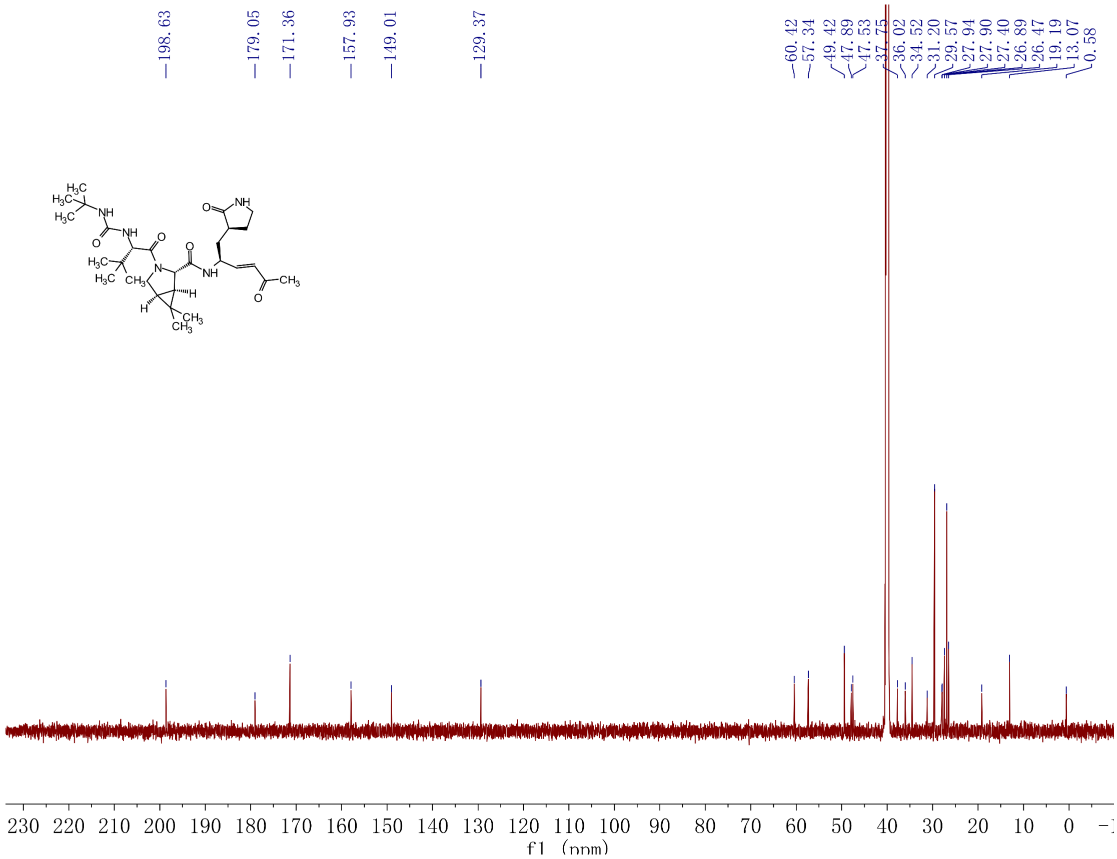

^{13}C NMR of **3**

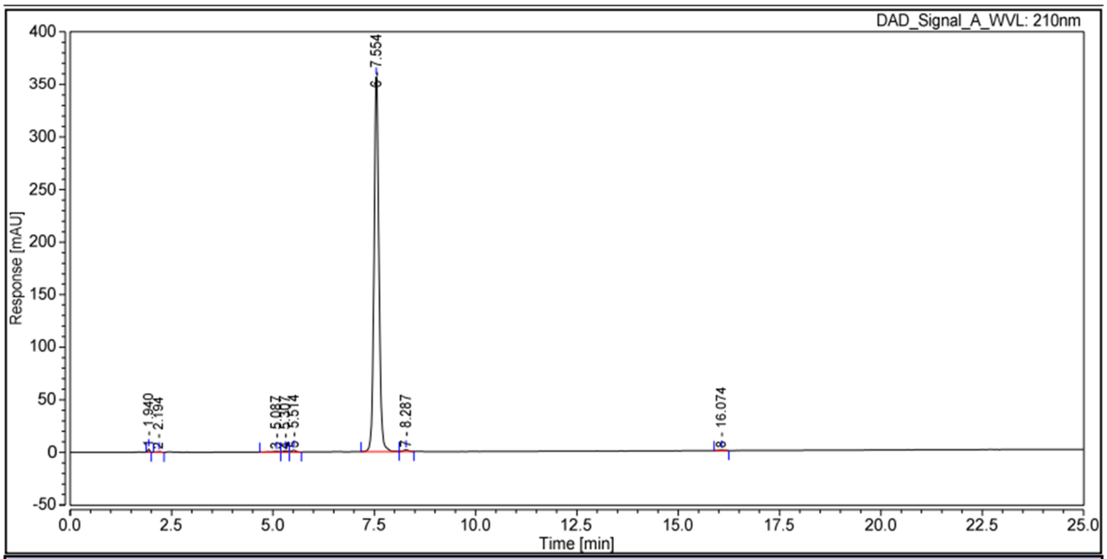

Integration Results

No.	Peak Width min	Retention Time min	Area mAU*s	Height mAU	Relative Area %	S/N	Resolution (EP)
1	0.07	1.940	9.27786	3.365	0.3055	159.2	1.90
2	0.17	2.194	3.72909	0.565	0.1228	26.7	13.45
3	0.24	5.087	8.49658	0.741	0.2798	35.1	1.02
4	0.20	5.307	9.19617	1.253	0.3028	59.3	1.10
5	0.18	5.514	12.25750	1.789	0.4036	84.7	10.41
6	0.21	7.554	2976.57107	356.349	98.0121	16865.9	3.36
7	0.22	8.287	13.94204	1.554	0.4591	73.6	33.75
8	0.24	16.074	3.47232	0.390	0.1143	18.4	n.a.
**Total:**			**3036.943**	**366.006**	**100.0000**	**17322.97**	

HPLC of **3**

Fragmentor Voltage	Collision Energy	Ionization Mode
170	0	ESI

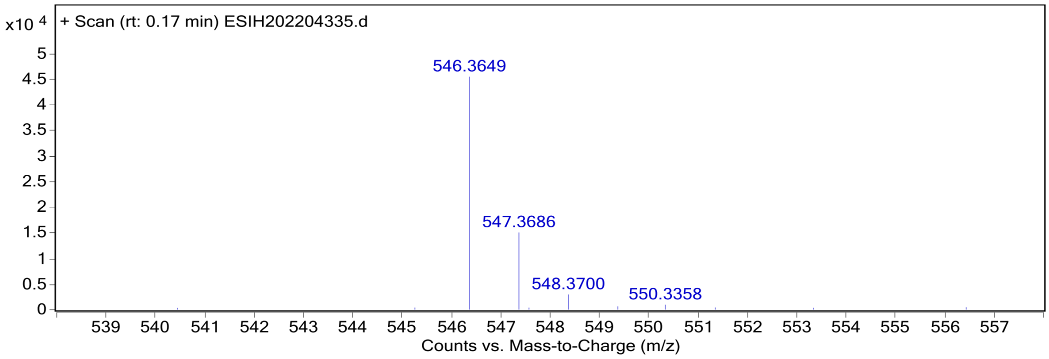

Formula Calculator Results

m/z	Calc m/z	Diff (mDa)	Diff (ppm)	Ion Formula	Ion
546.3649	546.365	0.07	0.12	C29 H48 N5 O5	(M+H)+

--- End Of Report ---

HRMS of **3**

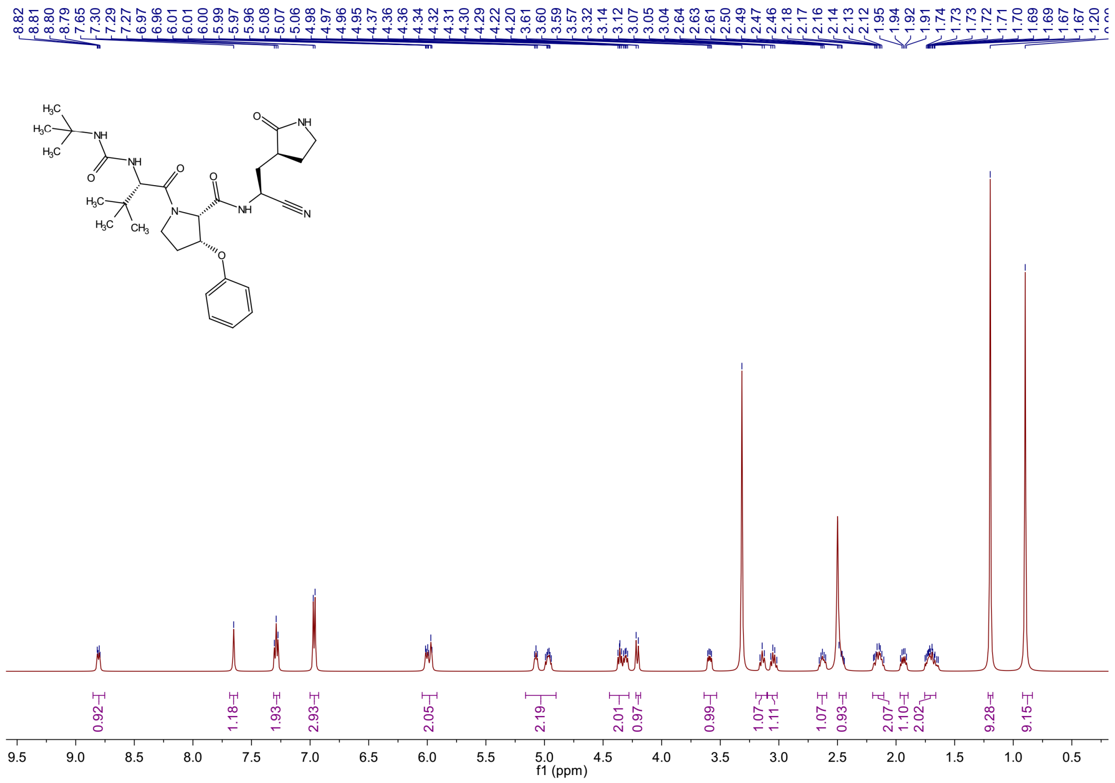

^{1}H NMR of **4**

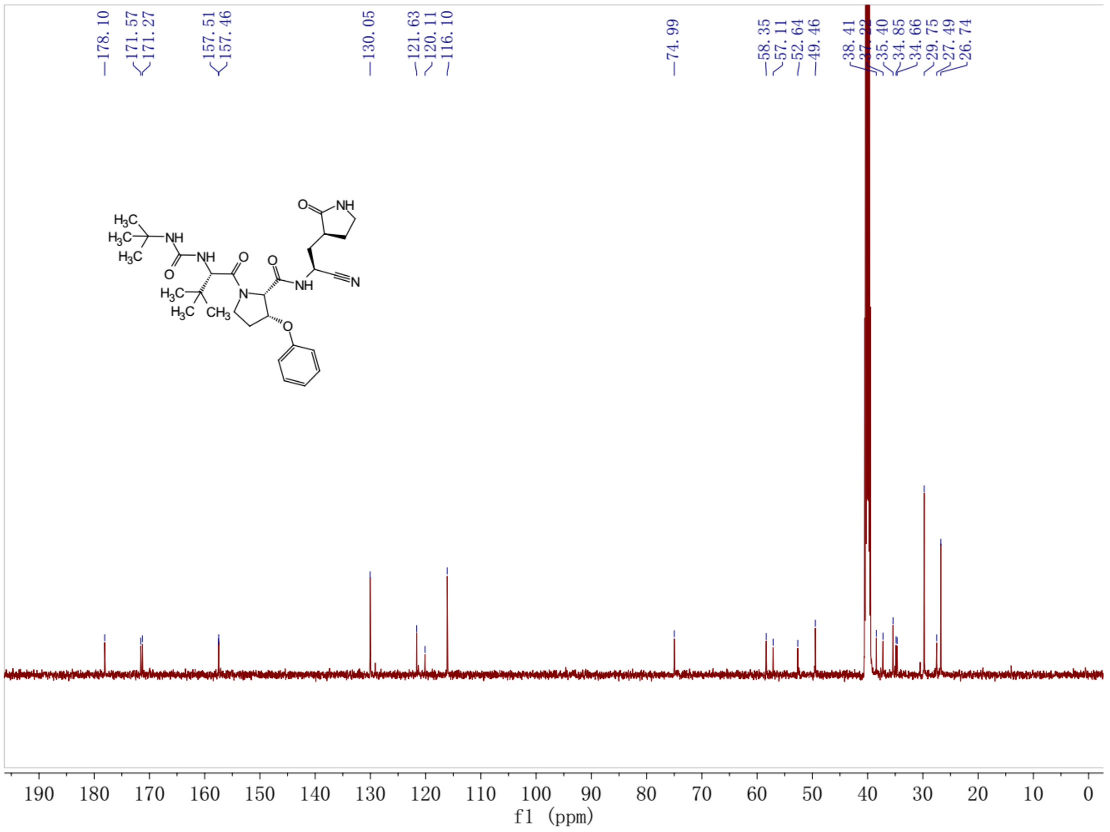

^{13}C NMR of **4**

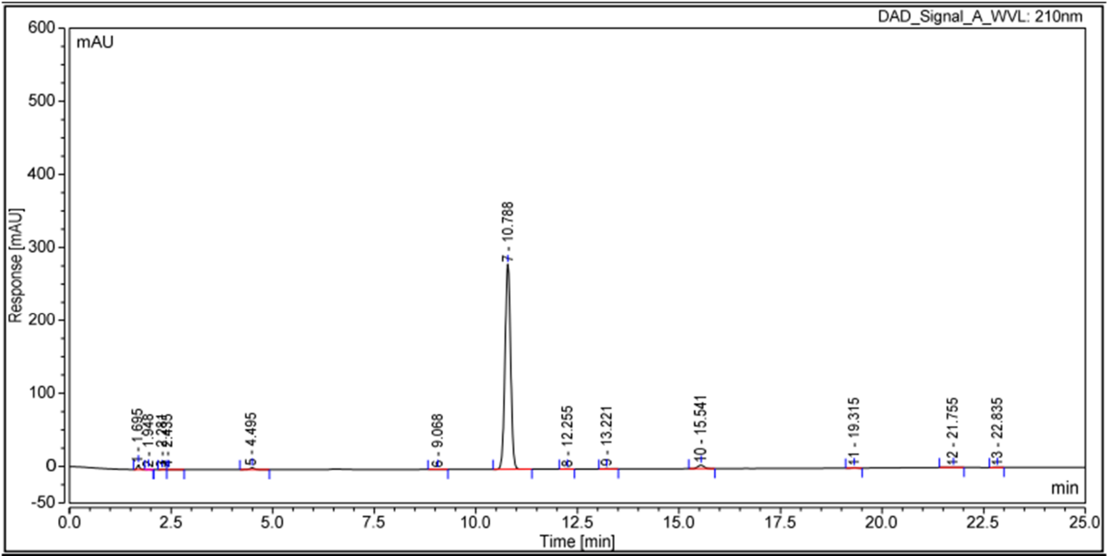

Integration Results

No.	Peak Name	Retention Time min	Area mAU*s	Height mAU	Relative Area %	S/N	Resolution (EP)
1		1.695	29.86031	6.716	1.0609	157.6	n.a.
2		1.948	3.62415	0.598	0.1288	14.0	n.a.
3		2.281	2.16971	0.244	0.0771	5.7	n.a.
4		2.435	1.18265	0.141	0.0420	3.3	n.a.
5		4.495	21.40865	2.532	0.7606	59.4	20.37
6		9.068	2.13633	0.213	0.0759	5.0	6.65
7		10.788	2684.14860	281.044	95.3676	6593.6	6.13
8		12.255	2.07891	0.241	0.0739	5.6	3.93
9		13.221	3.94154	0.390	0.1400	9.1	8.38
10		15.541	54.27084	4.920	1.9282	115.4	13.94
11		19.315	5.75180	0.609	0.2044	14.3	8.96
12		21.755	1.71986	0.136	0.0611	3.2	4.11
13		22.835	2.23618	0.254	0.0795	6.0	n.a.
**Total:**			**2814.530**	**298.038**	**100.0000**	**6992.26**	

HPLC of **4**

Fragmentor Voltage	Collision Energy	Ionization Mode
170	0	ESI

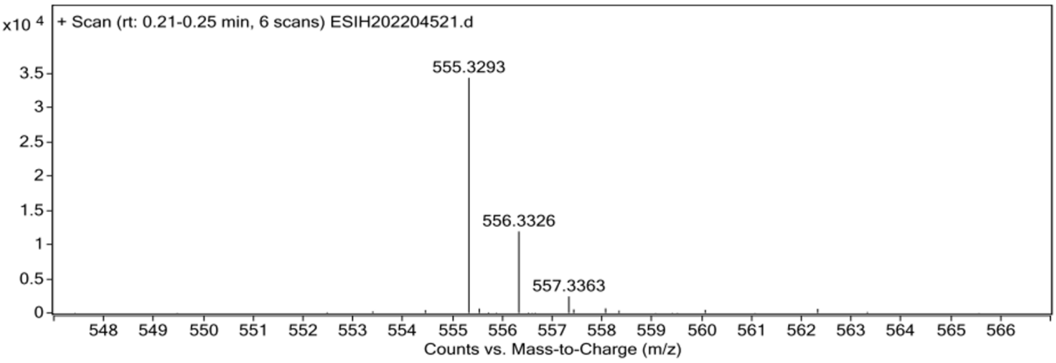

Formula Calculator Results

m/z	Calc m/z	Diff (mDa)	Diff (ppm)	Ion Formula	Ion
555.3293	555.3289	-0.4	-0.72	C29 H43 N6 O5	(M+H)+

--- End Of Report ---

HRMS of **4**

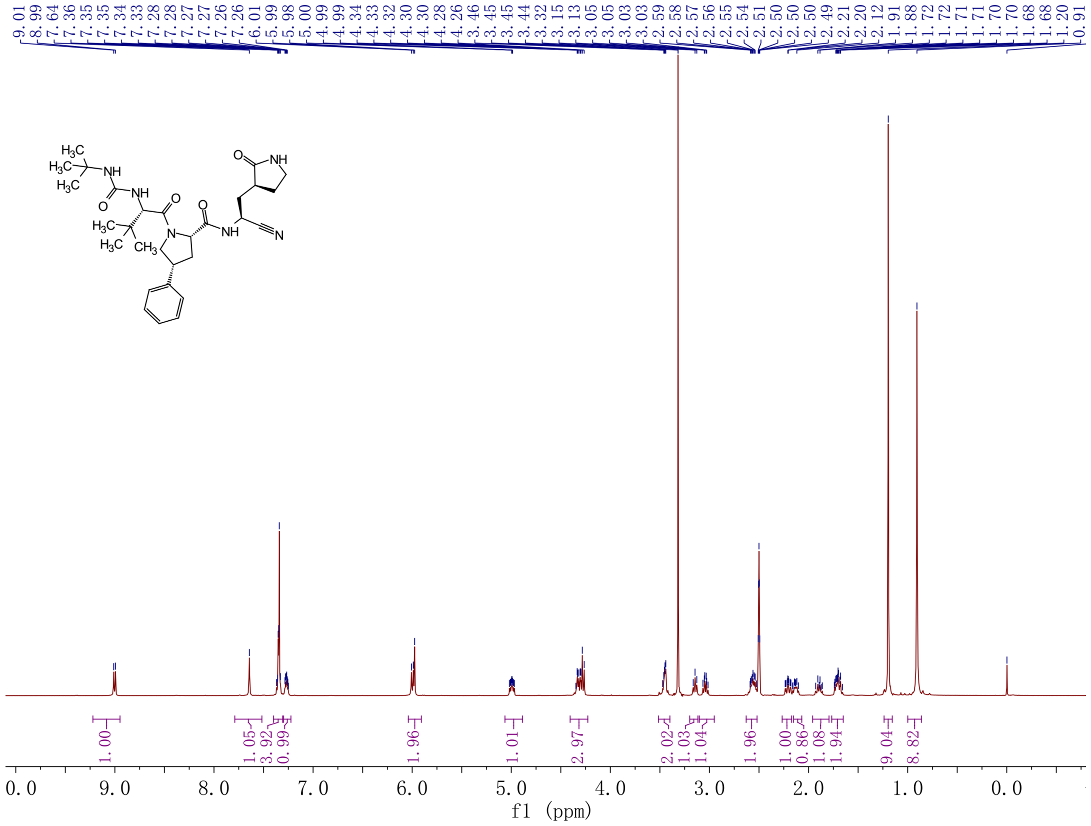

^{1}H NMR of **5**

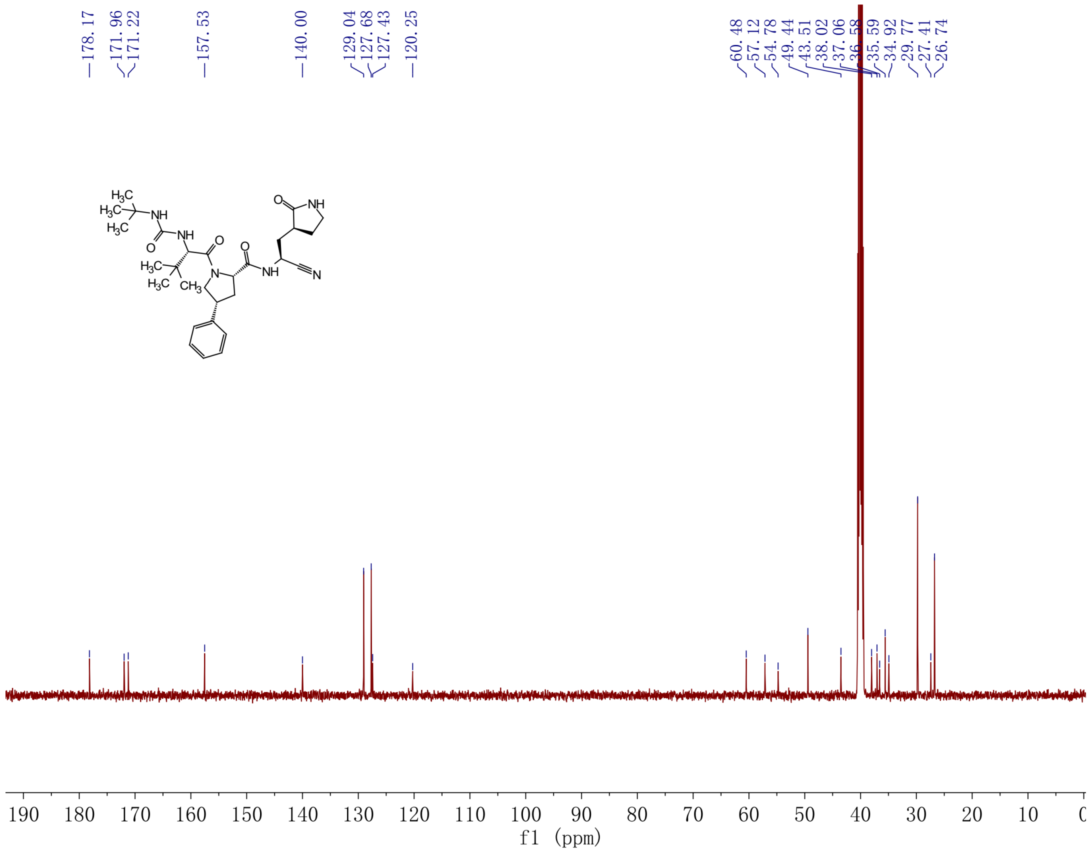

^{13}C NMR of **5**

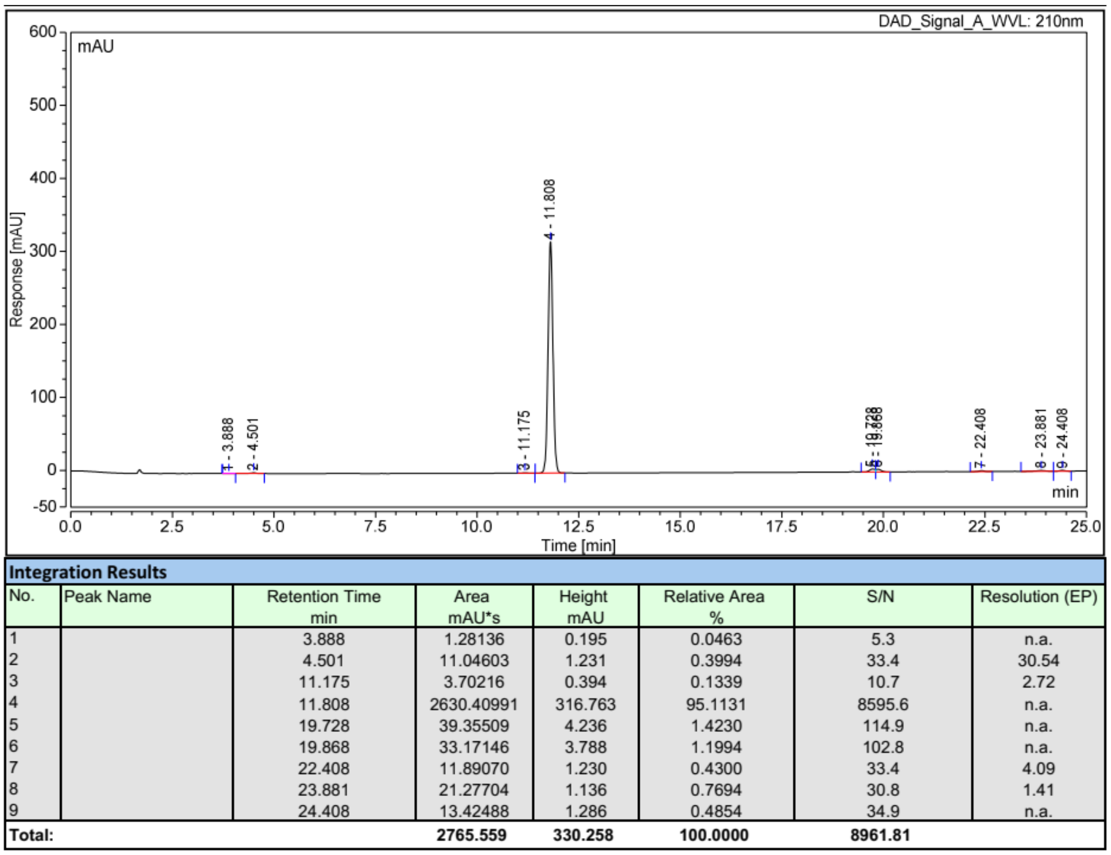

Integration Results

No.	Peak Name	Retention Time min	Area mAU*s	Height mAU	Relative Area %	S/N	Resolution (EP)
1		3.888	1.28136	0.195	0.0463	5.3	n.a.
2		4.501	11.04603	1.231	0.3994	33.4	30.54
3		11.175	3.70216	0.394	0.1339	10.7	2.72
4		11.808	2630.40991	316.763	95.1131	8595.6	n.a.
5		19.728	39.35509	4.236	1.4230	114.9	n.a.
6		19.868	33.17146	3.788	1.1994	102.8	n.a.
7		22.408	11.89070	1.230	0.4300	33.4	4.09
8		23.881	21.27704	1.136	0.7694	30.8	1.41
9		24.408	13.42488	1.286	0.4854	34.9	n.a.
**Total:**			**2765.559**	**330.258**	**100.0000**	**8961.81**	

HPLC of **5**

Fragmentor Voltage	Collision Energy	Ionization Mode
170	0	ESI

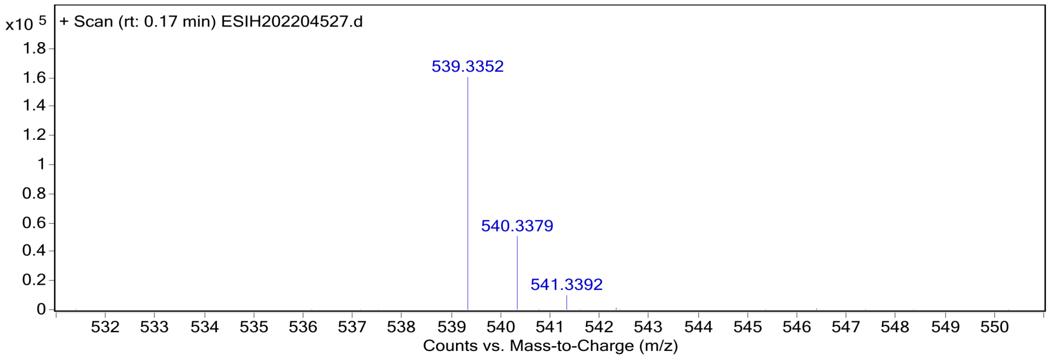

Formula Calculator Results

m/z	Calc m/z	Diff (mDa)	Diff (ppm)	Ion Formula	Ion
539.3352	539.334	-1.19	-2.21	C29 H43 N6 O4	(M+H)+

--- End Of Report ---

HRMS of **5**

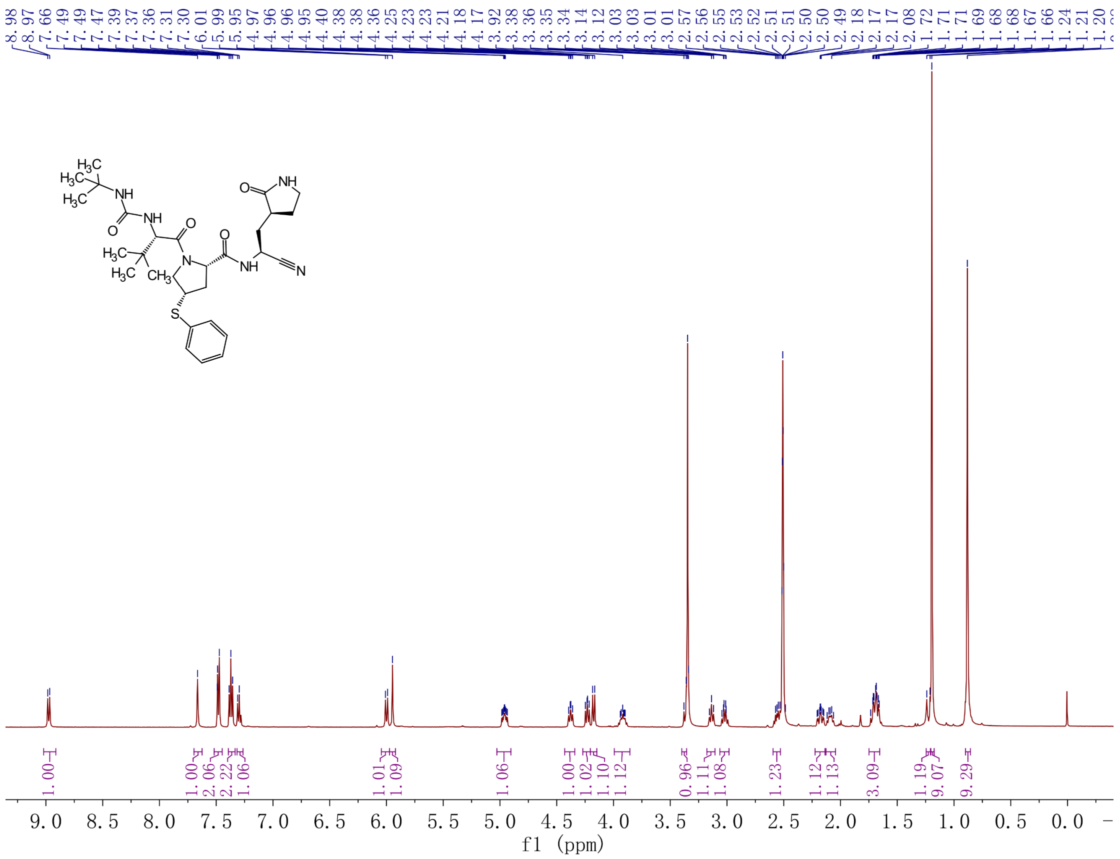

^{1}H NMR of **6**

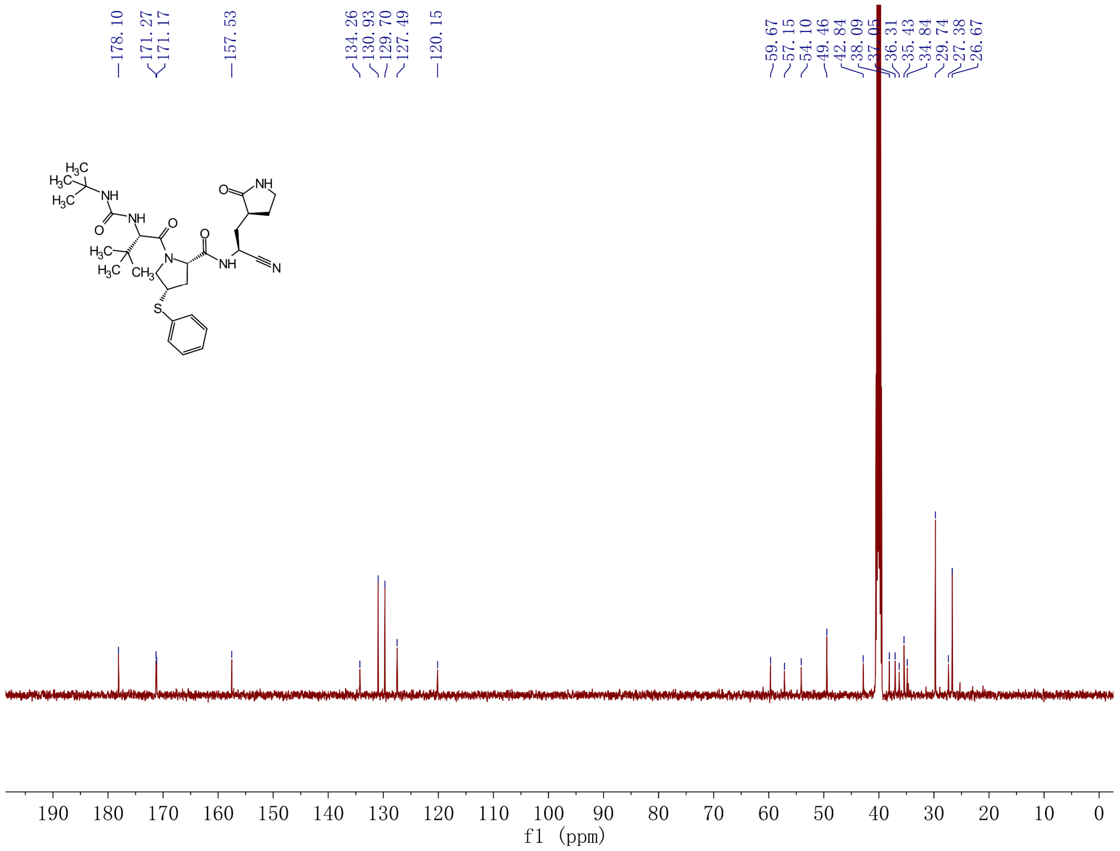

^{13}C NMR of **6**

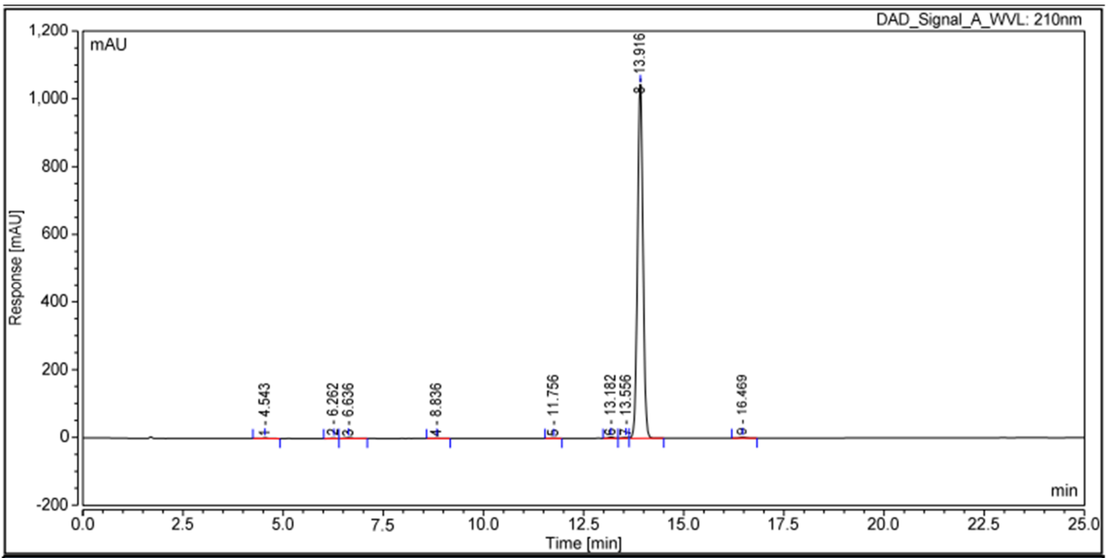

Integration Results

No.	Peak Name	Retention Time min	Area mAU*s	Height mAU	Relative Area %	S/N	Resolution (EP)
1		4.543	15.99655	1.836	0.1594	25.9	7.04
2		6.262	12.28754	1.110	0.1225	15.7	1.17
3		6.636	31.45998	2.271	0.3136	32.0	7.38
4		8.836	10.75783	1.091	0.1072	15.4	11.69
5		11.756	4.64191	0.502	0.0463	7.1	5.64
6		13.182	31.89006	3.180	0.3179	44.8	n.a.
7		13.556	20.55996	1.869	0.2049	26.3	n.a.
8		13.916	9879.11622	1045.165	98.4696	14736.4	9.97
9		16.469	25.94643	2.524	0.2586	35.6	n.a.
**Total:**			**10032.656**	**1059.547**	**100.0000**	**14939.15**	

HPLC of **6**

Fragmentor Voltage	Collision Energy	Ionization Mode
170	0	ESI

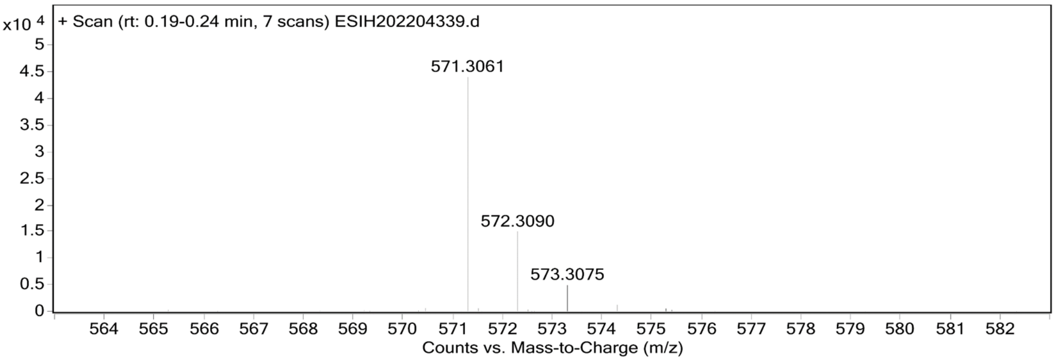

Formula Calculator Results

m/z	Calc m/z	Diff (mDa)	Diff (ppm)	Ion Formula	Ion
571.3061	571.3061	-0.04	-0.07	C29 H43 N6 O4 S	(M+H)+

--- End Of Report ---

HRMS of **6**

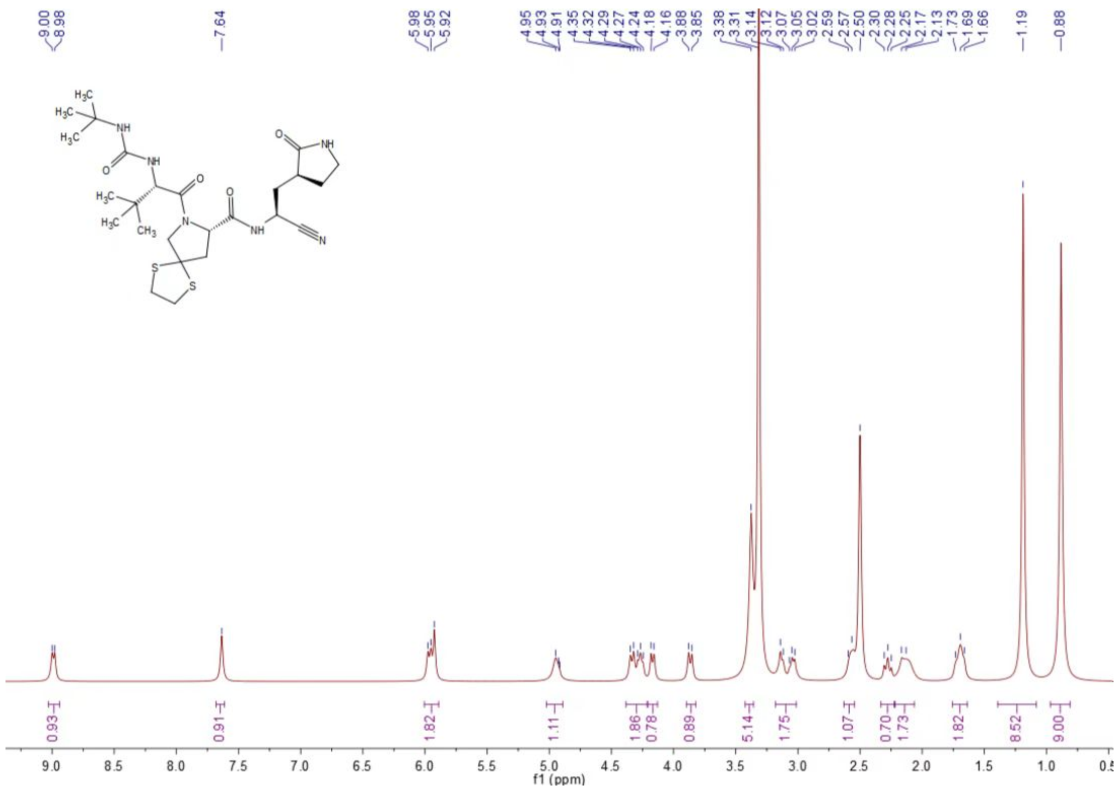

^{1}H NMR of **7**

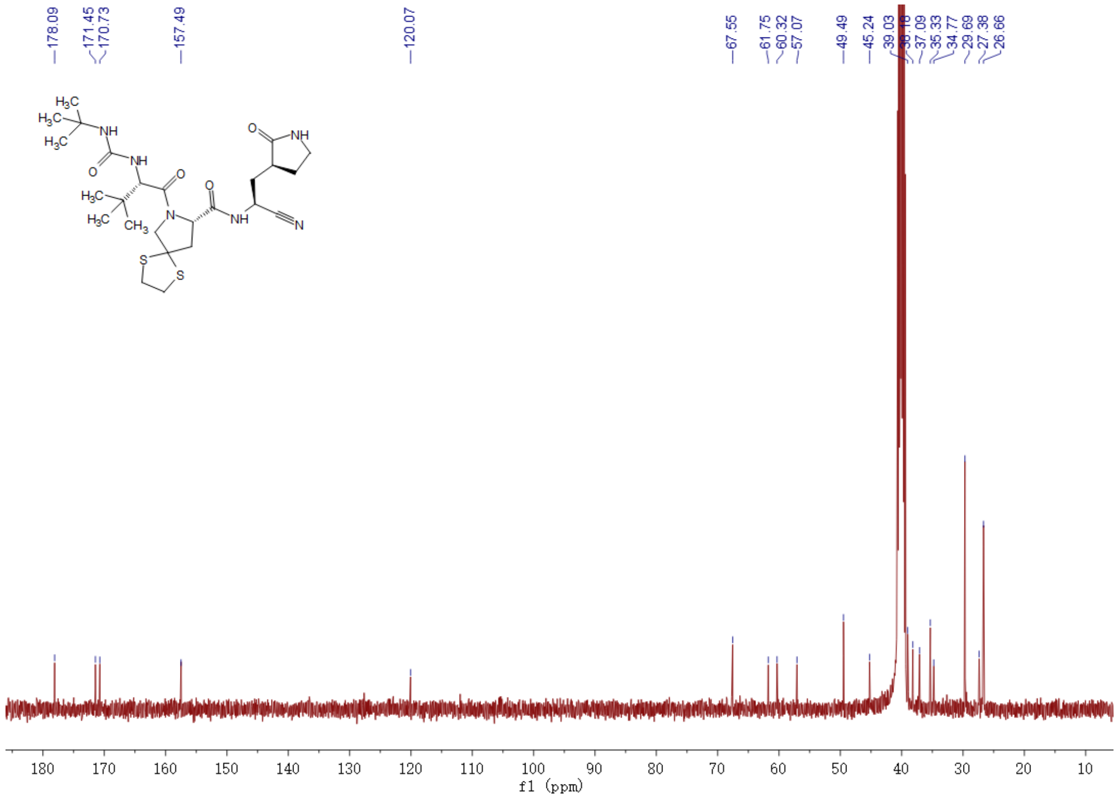

^{13}C NMR of **7**

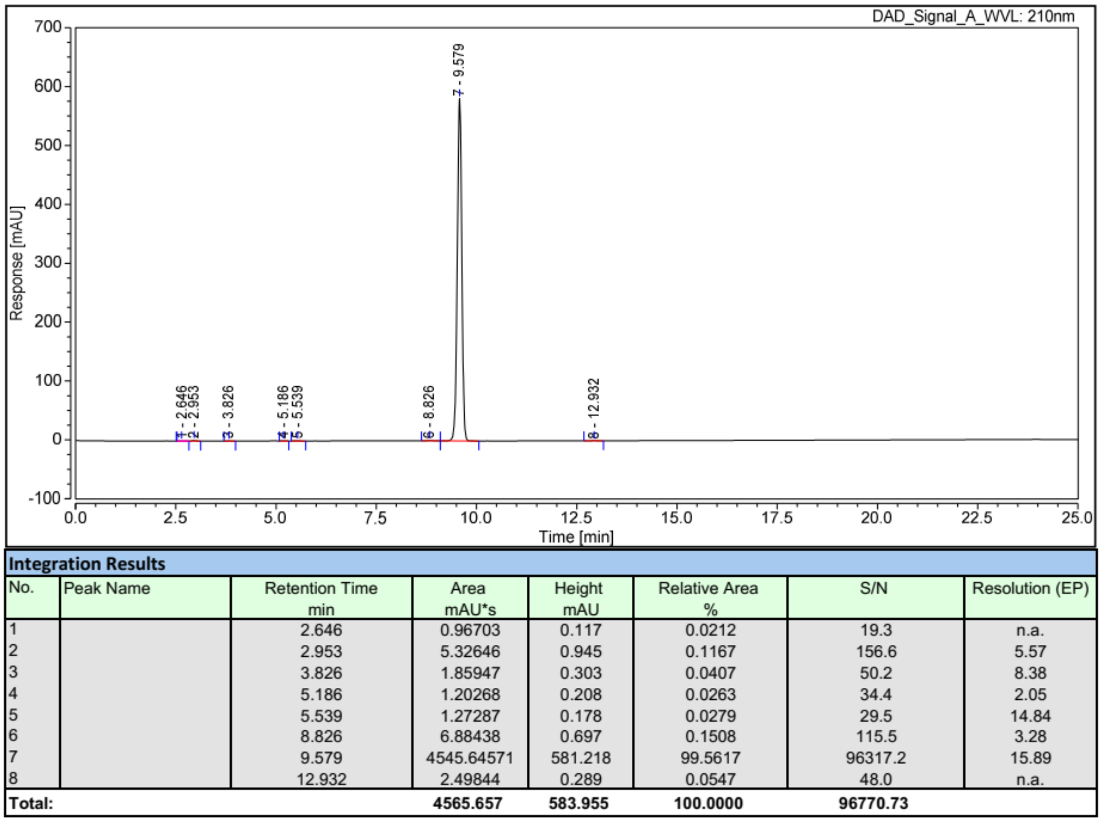

Integration Results

No.	Peak Name	Retention Time min	Area mAU*s	Height mAU	Relative Area %	S/N	Resolution (EP)
1		2.646	0.96703	0.117	0.0212	19.3	n.a.
2		2.953	5.32646	0.945	0.1167	156.6	5.57
3		3.826	1.85947	0.303	0.0407	50.2	8.38
4		5.186	1.20268	0.208	0.0263	34.4	2.05
5		5.539	1.27287	0.178	0.0279	29.5	14.84
6		8.826	6.88438	0.697	0.1508	115.5	3.28
7		9.579	4545.64571	581.218	99.5617	96317.2	15.89
8		12.932	2.49844	0.289	0.0547	48.0	n.a.
**Total:**			**4565.657**	**583.955**	**100.0000**	**96770.73**	

HPLC of **7**

Fragmentor Voltage	Collision Energy	Ionization Mode
225	0	ESI

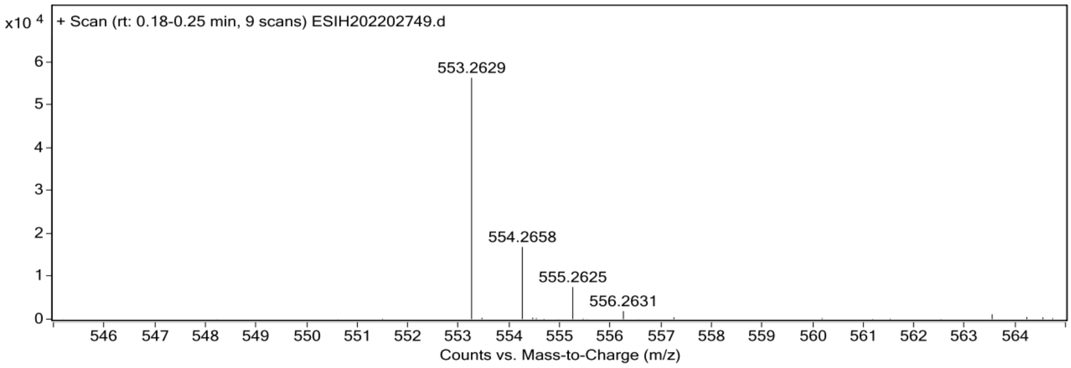

Formula Calculator Results

m/z	Calc m/z	Diff (mDa)	Diff (ppm)	Ion Formula	Ion
553.2629	553.2625	-0.41	-0.74	C25 H41 N6 O4 S2	(M+H)+

--- End Of Report ---

HRMS of **7**

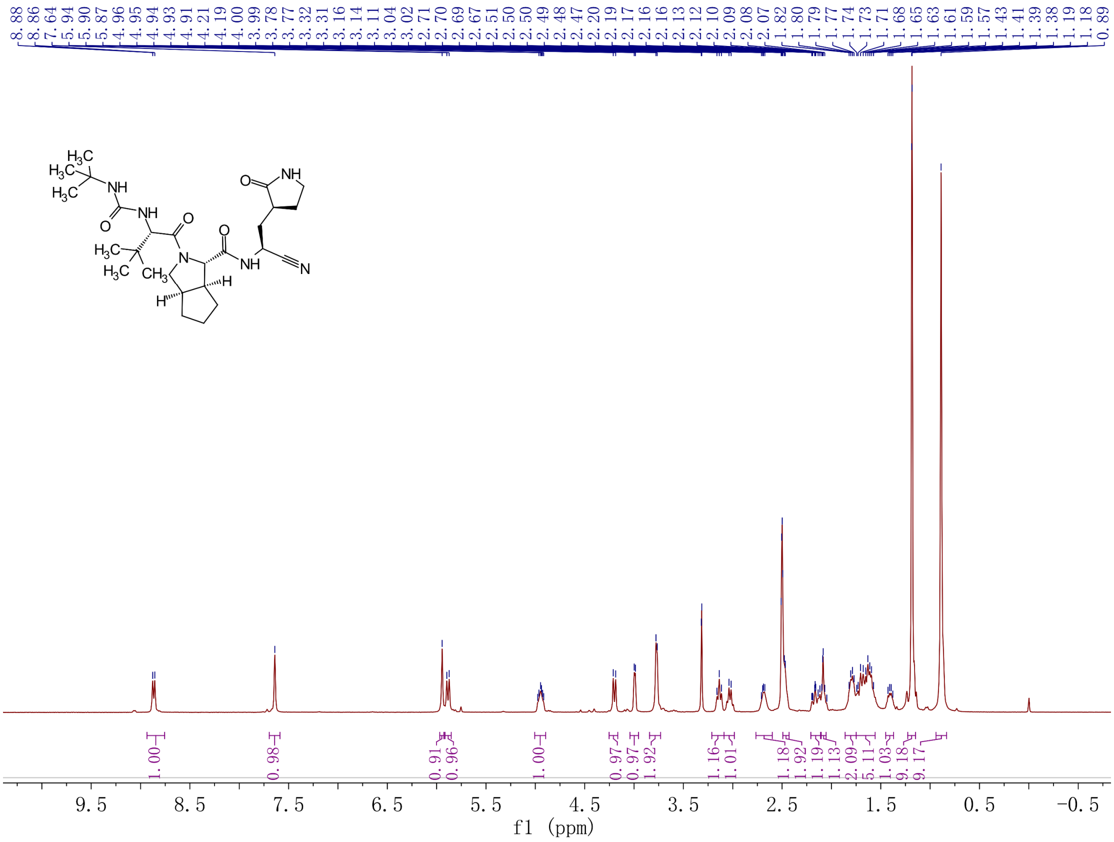

^{1}H NMR of **8**

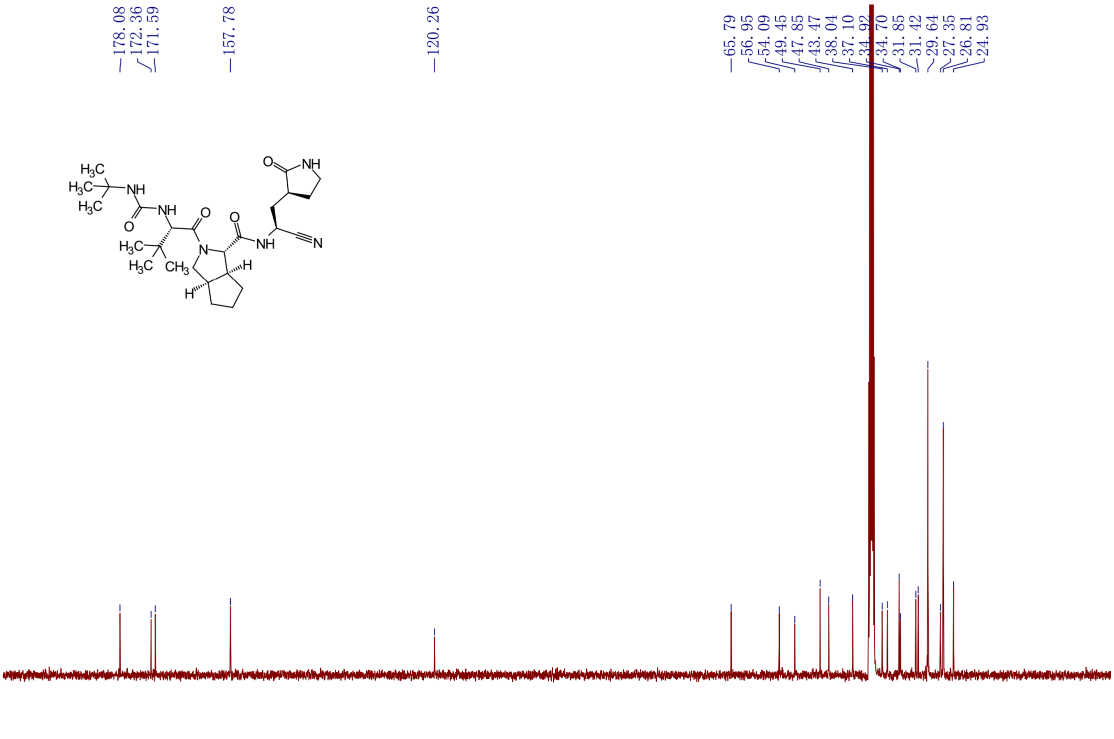

^{13}C NMR of **8**

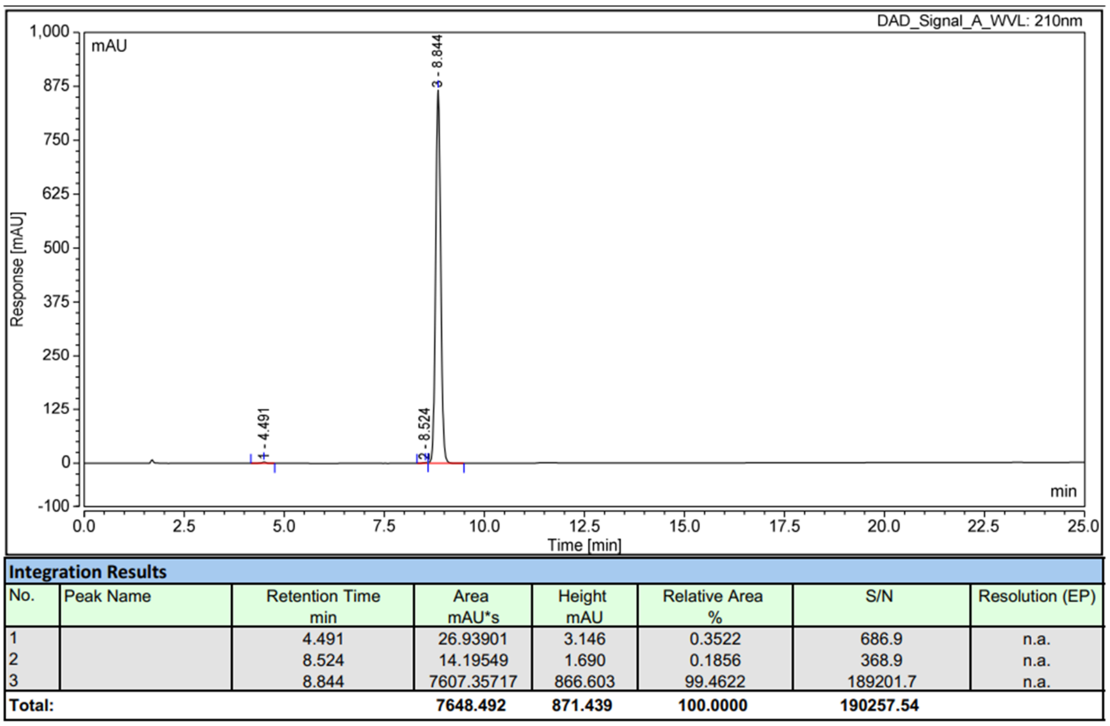

Integration Results

No.	Peak Name	Retention Time min	Area mAU*s	Height mAU	Relative Area %	S/N	Resolution (EP)
1		4.491	26.93901	3.146	0.3522	686.9	n.a.
2		8.524	14.19549	1.690	0.1856	368.9	n.a.
3		8.844	7607.35717	866.603	99.4622	189201.7	n.a.
**Total:**			**7648.492**	**871.439**	**100.0000**	**190257.54**	

HPLC of **8**

Fragmentor Voltage	Collision Energy	Ionization Mode
170	0	ESI

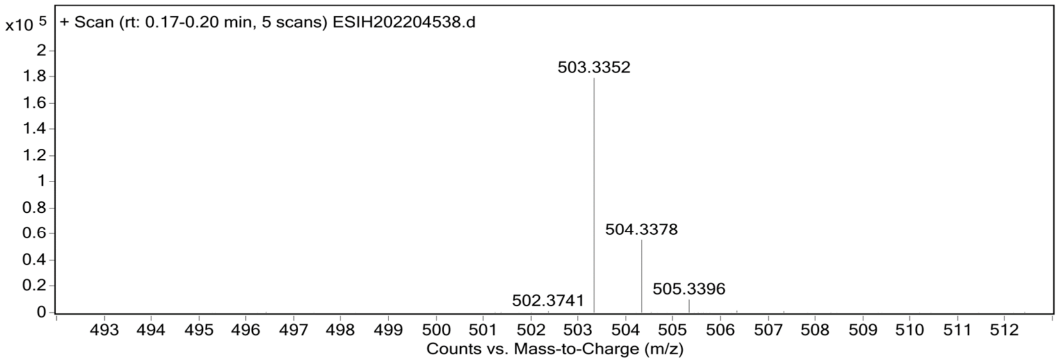

Formula Calculator Results

m/z	Calc m/z	Diff (mDa)	Diff (ppm)	Ion Formula	Ion
503.3352	503.334	-1.18	-2.34	C26 H43 N6 O4	(M+H)+

--- End Of Report ---

HRMS of **8**

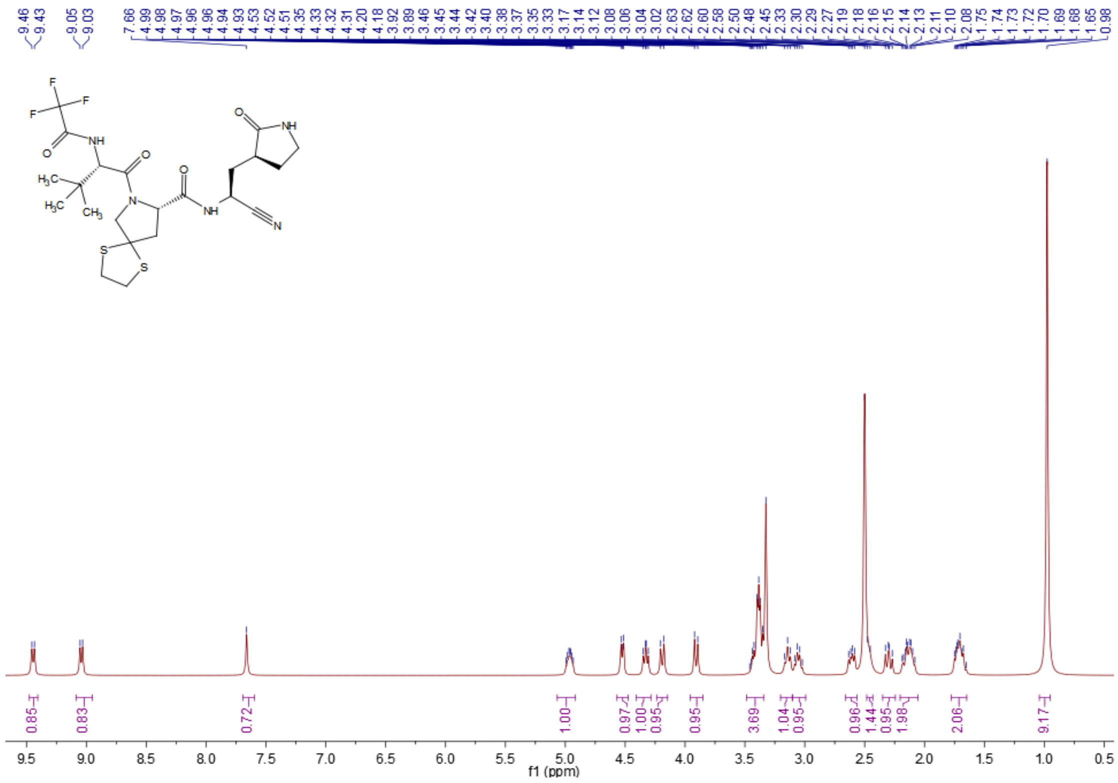

^{1}H NMR of **9**

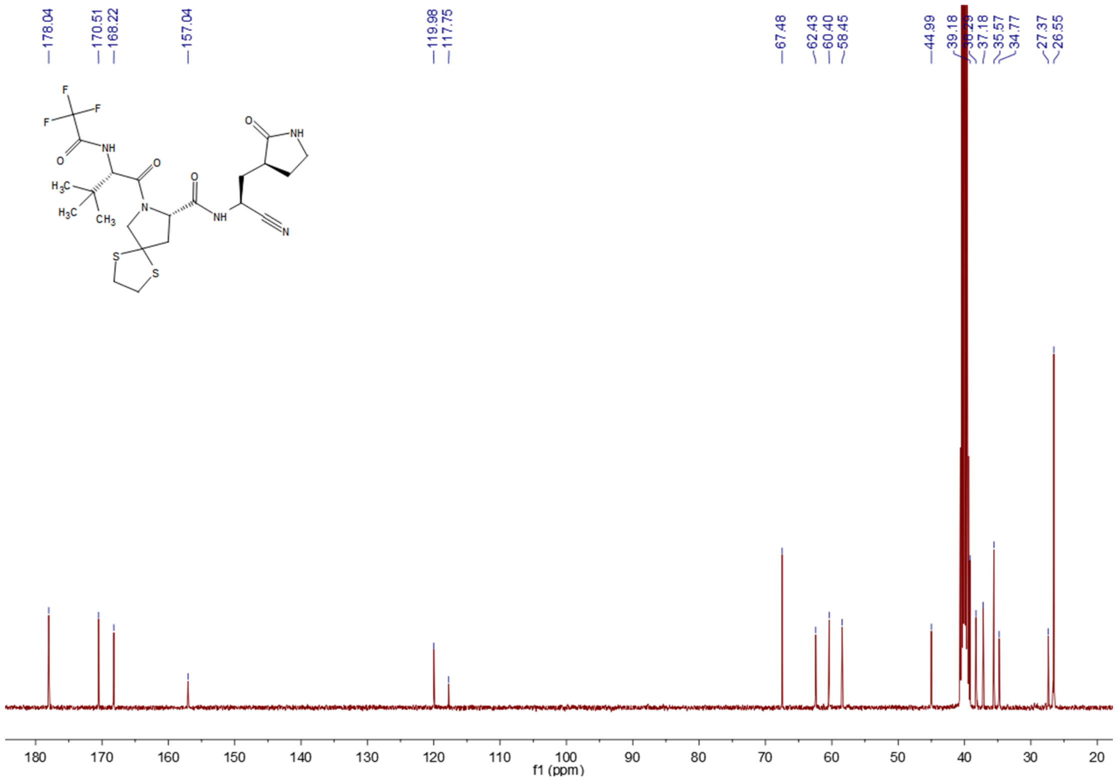

^{13}C NMR of **9**

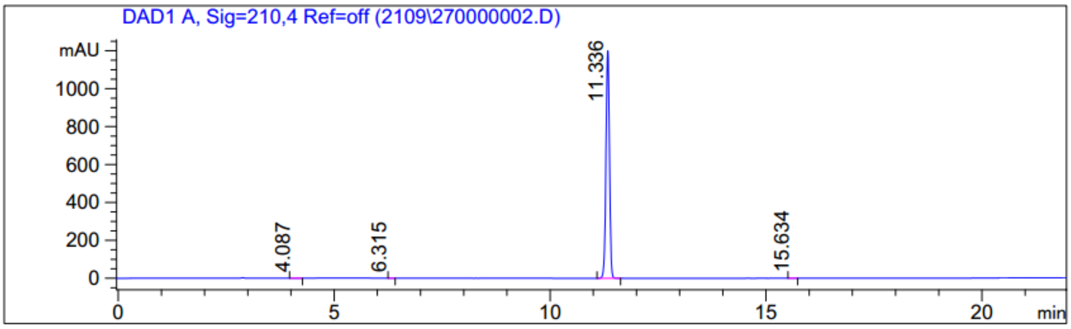

Peak #	RT [min]	Width [min]	Area [mAU*s]	Height [mAU]	Area %
1	4.087	0.1032	2.14293	0.30799	0.0316
2	6.315	0.0725	0.58775	0.11328	0.0087
3	11.336	0.0877	6771.52148	1203.29675	99.9009
4	15.634	0.0970	3.98947	0.63747	0.0589

HPLC of **9**

Fragmentor Voltage	Collision Energy	Ionization Mode
170	0	ESI

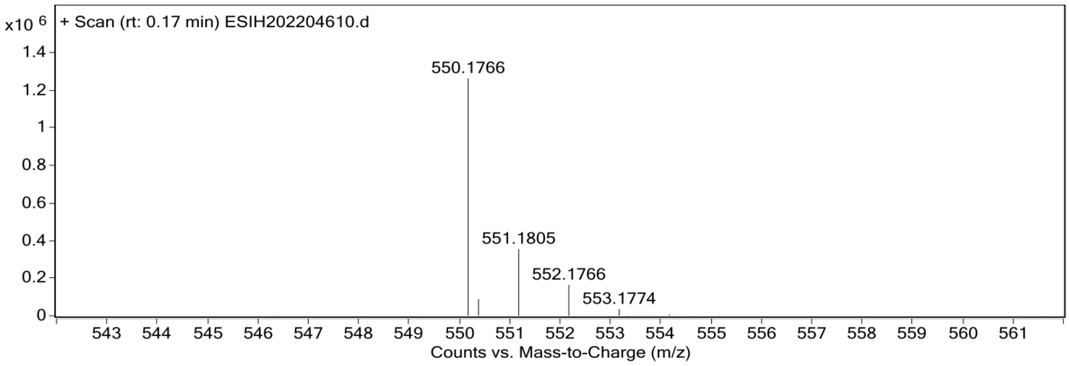

Formula Calculator Results

m/z	Calc m/z	Diff (mDa)	Diff (ppm)	Ion Formula	Ion
550.1766	550.1764	-0.24	-0.43	C22 H31 F3 N5 O4 S2	(M+H)+

--- End Of Report ---

HRMS of **9**

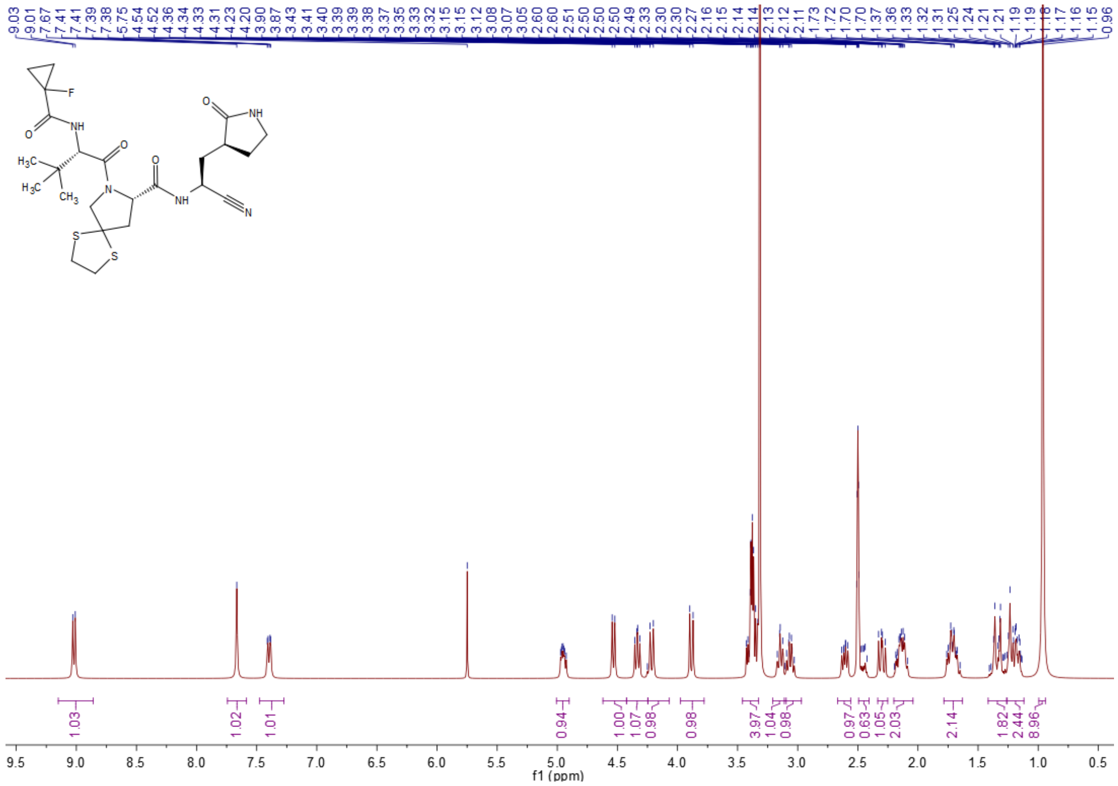

^{1}H NMR of **10**

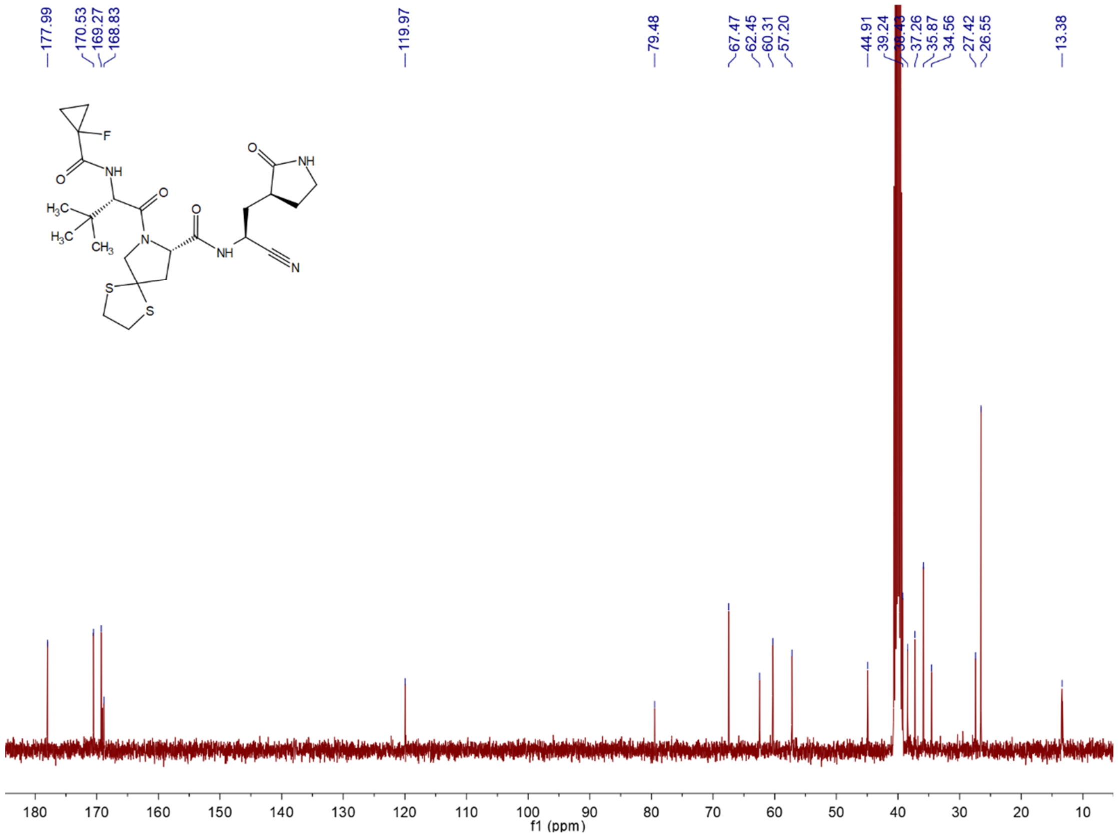

^{13}C NMR of **10**

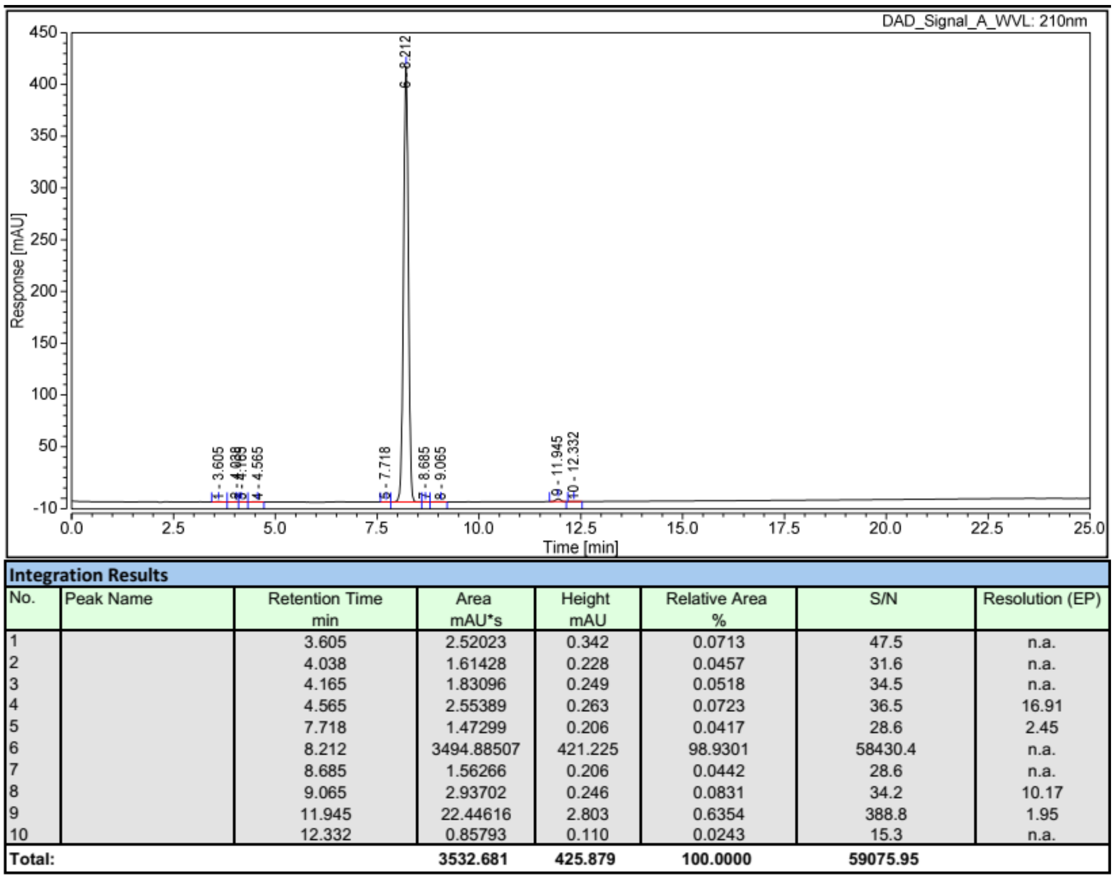

Integration Results

No.	Peak Name	Retention Time<br>min	Area<br>mAU*s	Height<br>mAU	Relative Area<br>%	S/N	Resolution (EP)
1		3.605	2.52023	0.342	0.0713	47.5	n.a.
2		4.038	1.61428	0.228	0.0457	31.6	n.a.
3		4.165	1.83096	0.249	0.0518	34.5	n.a.
4		4.565	2.55389	0.263	0.0723	36.5	16.91
5		7.718	1.47299	0.206	0.0417	28.6	2.45
6		8.212	3494.88507	421.225	98.9301	58430.4	n.a.
7		8.685	1.56266	0.206	0.0442	28.6	n.a.
8		9.065	2.93702	0.246	0.0831	34.2	10.17
9		11.945	22.44616	2.803	0.6354	388.8	1.95
10		12.332	0.85793	0.110	0.0243	15.3	n.a.
**Total:**			**3532.681**	**425.879**	**100.0000**	**59075.95**	

HPLC of **10**

**Fragmentor Voltage**	**Collision Energy**	**Ionization Mode**
175	0	ESI

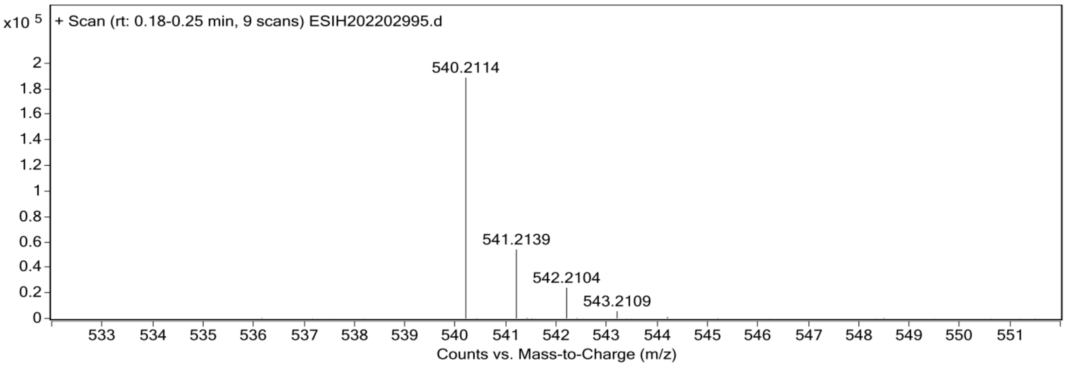

Formula Calculator Results

m/z	Calc m/z	Diff (mDa)	Diff (ppm)	Ion Formula	Ion
540.2114	540.2109	-0.48	-0.89	C24 H35 F N5 O4 S2	(M+H)+

--- End Of Report ---

HRMS of **10**

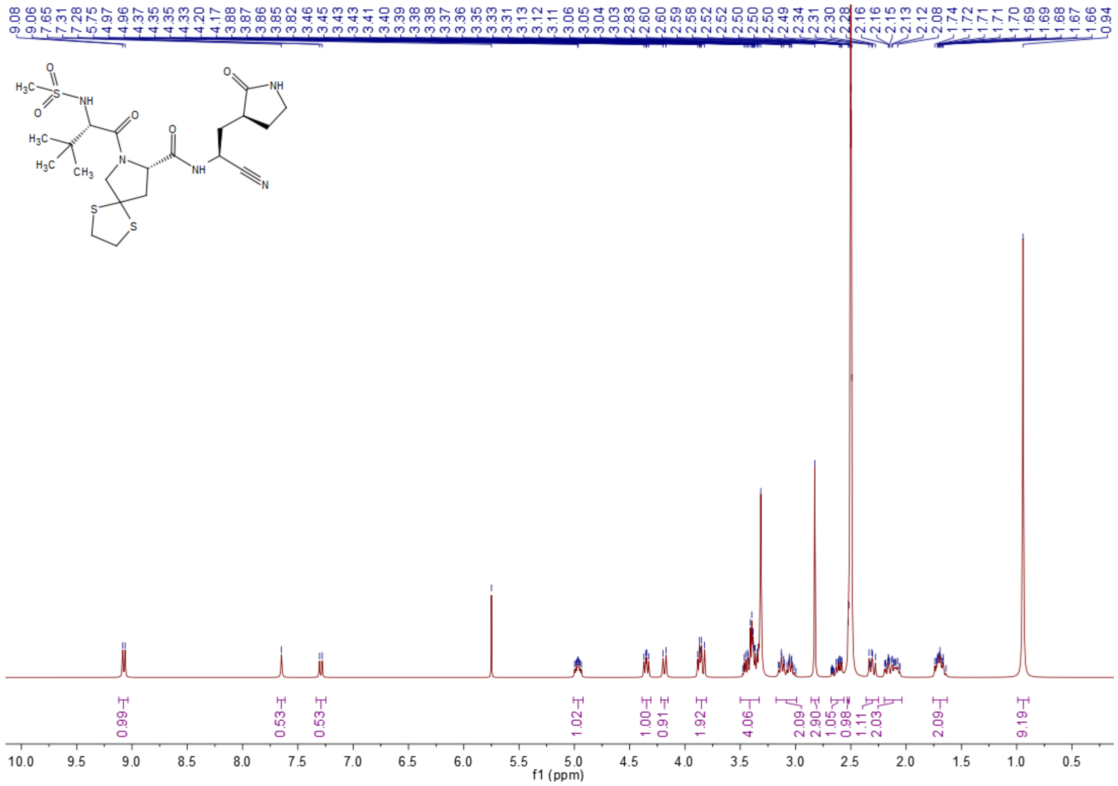

^{1}H NMR of **11**

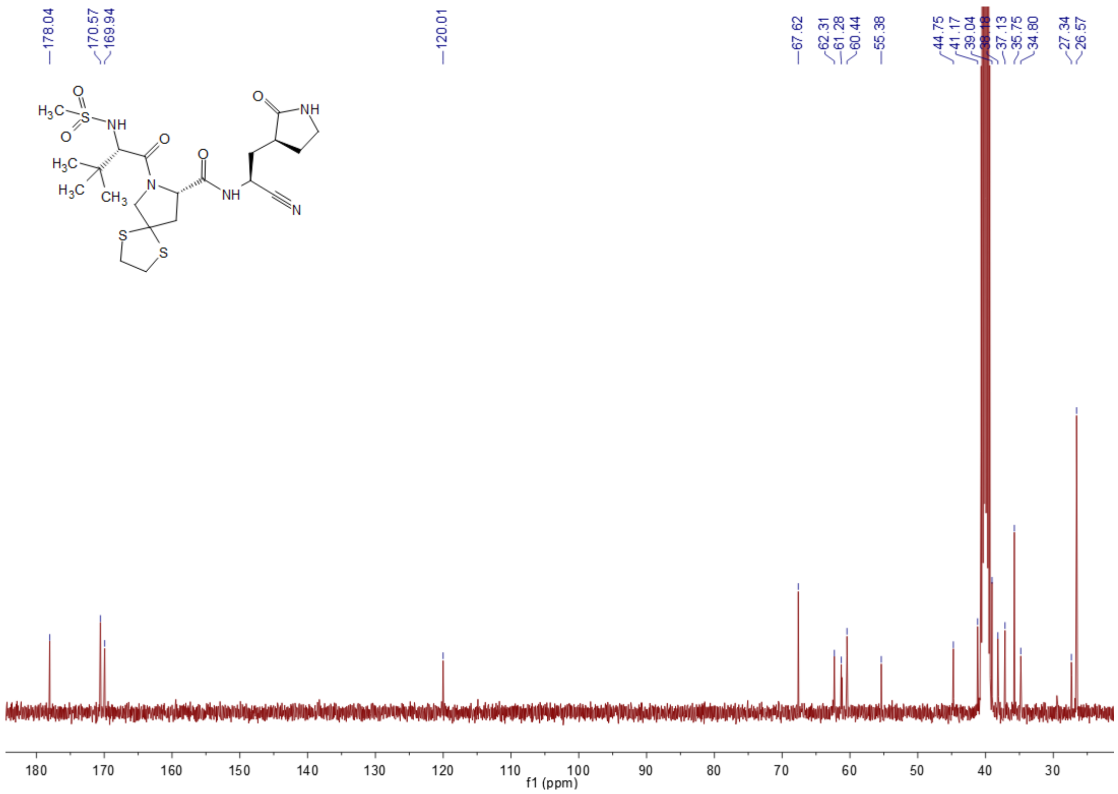

^{13}C NMR of **11**

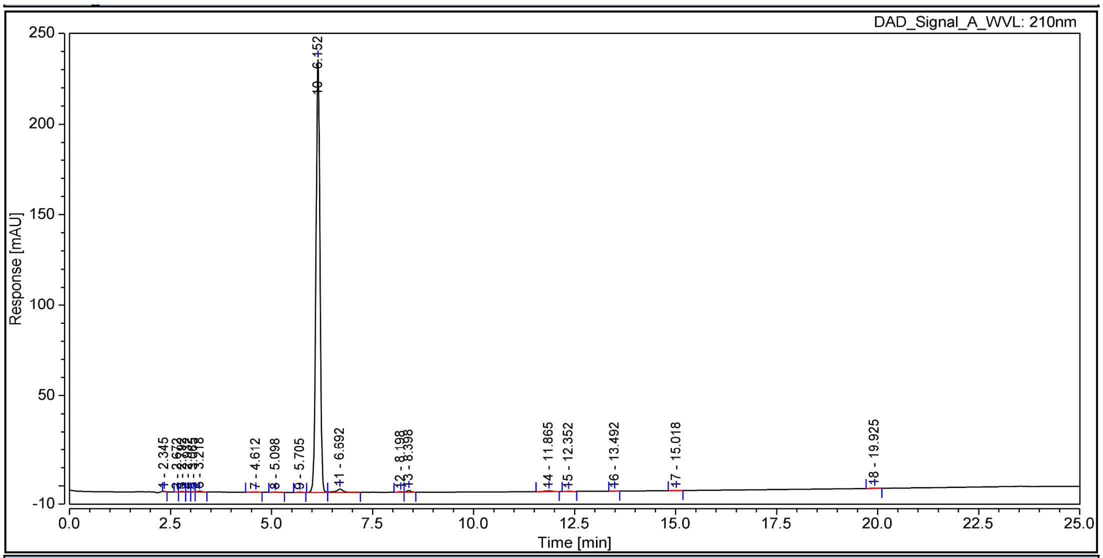

Integration Results

No.	Peak Name	Retention Time min	Area mAU*s	Height mAU	Relative Area %	S/N	Resolution (EP)
1		2.345	0.91009	0.300	0.0537	33.0	n.a.
2		2.672	0.36653	0.092	0.0216	10.2	n.a.
3		2.792	2.21101	0.377	0.1304	41.5	1.08
4		2.932	1.02245	0.257	0.0603	28.3	1.12
5		3.065	0.86020	0.200	0.0507	22.1	0.91
6		3.218	3.11457	0.404	0.1837	44.5	6.15
7		4.612	2.03991	0.205	0.1203	22.5	1.87
8		5.098	1.55163	0.170	0.0915	18.7	2.69
9		5.705	1.20697	0.183	0.0712	20.2	2.50
10		6.152	1642.46894	239.063	96.8762	26308.1	2.51
11		6.692	22.04923	1.845	1.3005	203.0	n.a.
12		8.198	2.08549	0.267	0.1230	29.4	n.a.
13		8.398	7.35665	0.997	0.4339	109.7	13.43
14		11.865	5.04408	0.406	0.2975	44.7	1.78
15		12.352	0.67387	0.076	0.0397	8.4	5.42
16		13.492	0.79269	0.108	0.0468	11.9	7.19
17		15.018	0.70648	0.076	0.0417	8.4	20.34
18		19.925	0.97027	0.099	0.0572	10.8	n.a.
**Total:**			**1695.431**	**245.125**	**100.0000**	**26975.27**	

HPLC of **11**

Fragmentor Voltage	Collision Energy	Ionization Mode
175	0	ESI

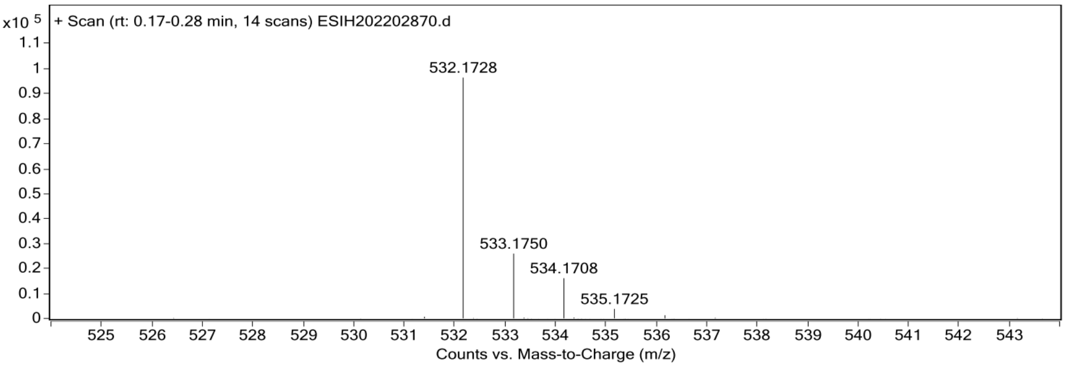

Formula Calculator Results

m/z	Calc m/z	Diff (mDa)	Diff (ppm)	Ion Formula	Ion
532.1728	532.1717	-1.12	-2.1	C21 H34 N5 O5 S3	(M+H)+

--- End Of Report ---

HRMS of **11**

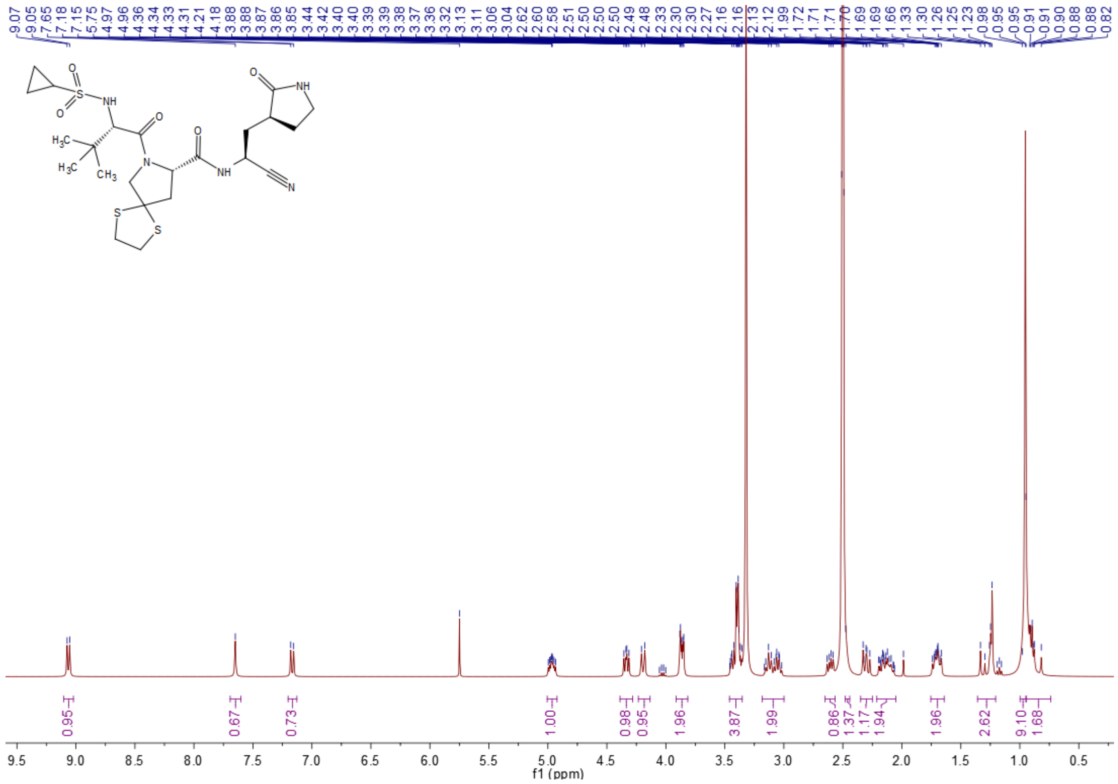

^{1}H NMR of **12**

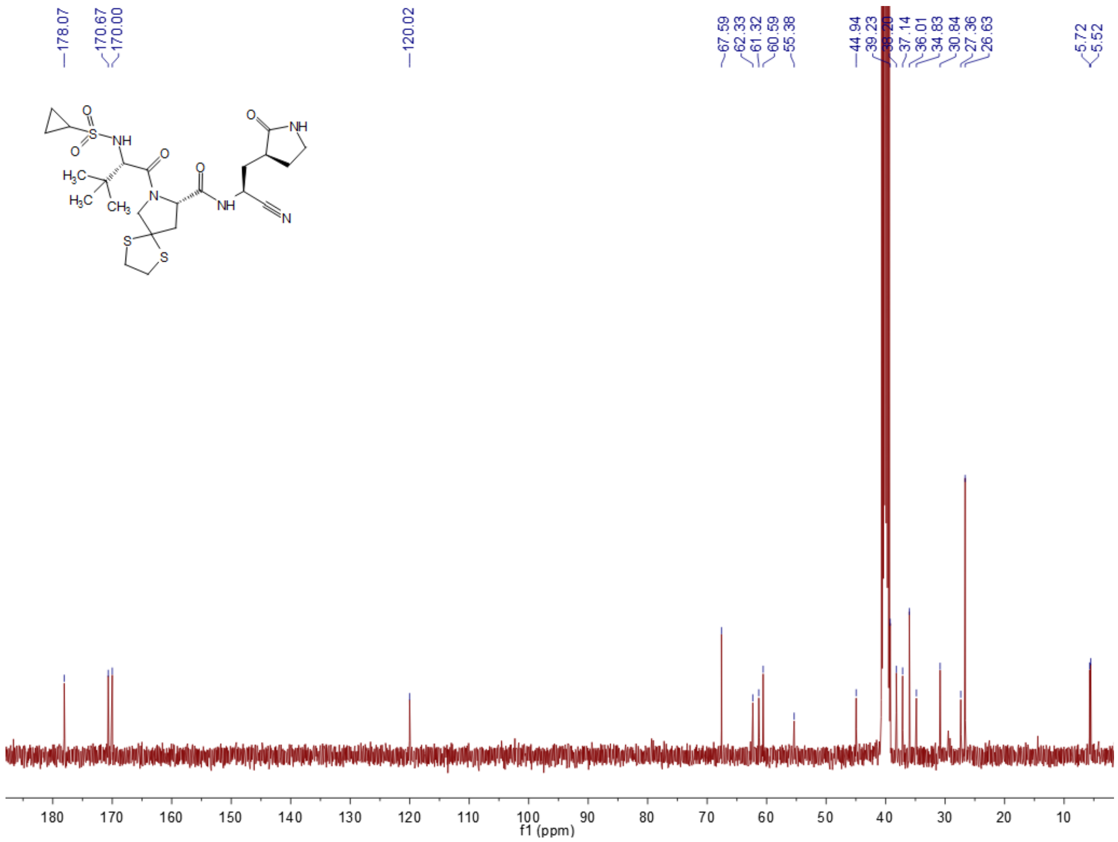

^{13}C NMR of **12**

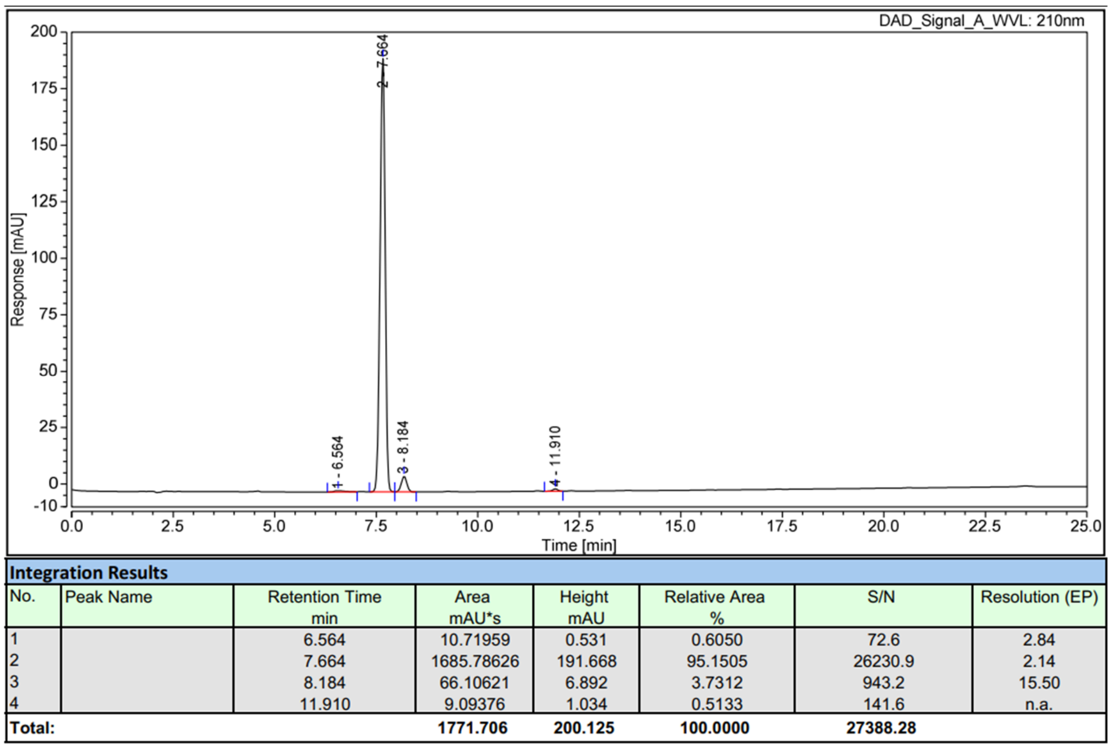

Integration Results

No.	Peak Name	Retention Time min	Area mAU*s	Height mAU	Relative Area %	S/N	Resolution (EP)
1		6.564	10.71959	0.531	0.6050	72.6	2.84
2		7.664	1685.78626	191.668	95.1505	26230.9	2.14
3		8.184	66.10621	6.892	3.7312	943.2	15.50
4		11.910	9.09376	1.034	0.5133	141.6	n.a.
**Total:**			**1771.706**	**200.125**	**100.0000**	**27388.28**	

HPLC of **12**

**Fragmentor Voltage**	**Collision Energy**	**Ionization Mode**
225	0	ESI

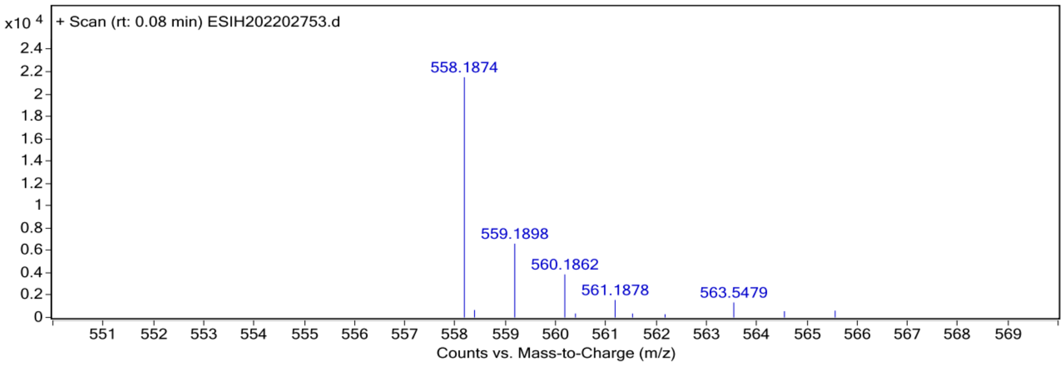

Formula Calculator Results

m/z	Calc m/z	Diff (mDa)	Diff (ppm)	Ion Formula	Ion
558.1874	558.1873	-0.06	-0.11	C23 H36 N5 O5 S3	(M+H)+

--- End Of Report ---

HRMS of **12**
