# Supplementary material for: Structure-based development and preclinical evaluation of the SARS-CoV-2 3C-like protease inhibitor simnotrelvir
Source: Nat Commun. 2023 Oct 13;14:6463. doi: 10.1038/s41467-023-42102-y (PMC10575921; doi:10.1038/s41467-023-42102-y)
Supplement: Supplementary file 3 — Reporting Summary [file 41467_2023_42102_MOESM3_ESM.pdf]

nature portfolio

Corresponding author(s): Renhong Tang, Leike Zhang, Jingshan Shen & Yechun Xu

Last updated by author(s): Aug 22, 2023

Reporting Summary

Nature Portfolio wishes to improve the reproducibility of the work that we publish. This form provides structure for consistency and transparency in reporting. For further information on Nature Portfolio policies, see our Editorial Policies and the Editorial Policy Checklist.

Statistics

For all statistical analyses, confirm that the following items are present in the figure legend, table legend, main text, or Methods section.

n/a	Confirmed	
☐	☒	The exact sample size (*n*) for each experimental group/condition, given as a discrete number and unit of measurement
☐	☒	A statement on whether measurements were taken from distinct samples or whether the same sample was measured repeatedly
☐	☒	The statistical test(s) used AND whether they are one- or two-sided<br>*Only common tests should be described solely by name; describe more complex techniques in the Methods section.*
☒	☐	A description of all covariates tested
☒	☐	A description of any assumptions or corrections, such as tests of normality and adjustment for multiple comparisons
☐	☒	A full description of the statistical parameters including central tendency (e.g. means) or other basic estimates (e.g. regression coefficient) AND variation (e.g. standard deviation) or associated estimates of uncertainty (e.g. confidence intervals)
☐	☒	For null hypothesis testing, the test statistic (e.g. *F*, *t*, *r*) with confidence intervals, effect sizes, degrees of freedom and *P* value noted<br>*Give P values as exact values whenever suitable.*
☒	☐	For Bayesian analysis, information on the choice of priors and Markov chain Monte Carlo settings
☒	☐	For hierarchical and complex designs, identification of the appropriate level for tests and full reporting of outcomes
☒	☐	Estimates of effect sizes (e.g. Cohen's *d*, Pearson's *r*), indicating how they were calculated

Our web collection on statistics for biologists contains articles on many of the points above.

Software and code

Policy information about availability of computer code

Data collection	For hERG study, data collection was performed using Patchcontrol HT (2.01.26) software and Patchmaster (V2 ×90.3 beta) software.<br>The data of clinical observations, body weight, food consumption, hematology, plasma chemistry, coagulation, urinalysis, terminal body weight, organ weights and histopathology in the two-week GLP-compliant toxicity studies in rats and monkeys recorded using Provantis (v10.2.3.1). Toxicokinetics parameters were collected using LCMS system Analyst software (v1.7) and Watson LIMS (v7.5 SP1). Cardiovascular system safety pharmacology parameters (HR, PR interval, QRS duration, QT interval, QTcF interval, and blood pressure) were collected using Iox2 (v10.0.40).<br>NMR data was recorded on a Bruker Avance 800 MHz, a Bruker Avance 600 MHz, a Bruker Avance 500 MHz or a Bruker Avance 400 MHz instrument using TopSpin software (v3.0 or v3.2 or v3.5) .<br>HPLC spectra were collected on an Agilent1100 system using Chemstation(B.04.02) software or an Agilent1260 Infinity II HPLC system using Chromeleon (7.2 SR5) software.<br>ESIMS spectra were collected on a Thermo Fisher FINNIGAN LTQ spectrometer using Xcalibur software (v2.0.5).<br>HRESIMS data were recorded on an Agilent G6520 Q-TOF mass spectrometer and using MassHuter Workstation software (vB.05.01).
Data analysis	The data of hERG current were analyzed using Igor Pro software (v6.2.2.2).<br>Plasma concentrations of simnotrelvir in mice, rats, and monkeys were analyzed using Phoenix WinNonlin (v7.0).<br>For CYP inhibition study, data were analyzed using Analyst (v1.7.1) and GraphPad Prism (v9.1.2).<br>The data collected from two-week GLP-compliant toxicity studies were analyzed using Provantis (v10.2.3.1). Cardiovascular system safety pharmacology data were analyzed using ecgAUTO (v3.3.5.10). Toxicokinetics parameters were analyzed using WinNonlin (v6.3).<br>The data from in vitro Ames test, chromosome aberration test and in vivo micronucleus test were analyzed using SPSS Statistics (v21).<br>Graphpad Prism software (v9.1.2) or XLfit (v5.5.0.5) was used to determine the IC50, EC50 and GSHt1/2 values.

For determination and analysis of crystal structures, we used HKL3000, CCP4 (v7.0.078), COOT (v0.8.9.2), PHENIX (v1.17.1-3660), Pymol (v2.4.0), and LigPlot (v2.2.8).
NMR data for all compounds was analyzed with MestReNova (v12.0.0-20080).
Purity analysis of compounds was analyzed with ChemStation (vB.04.02) or Chromeleon (v7.2 SR5);
The Xcalibur (v2.0.5) sofeware was used to analyze the ESIMS and HRESIMS data for all compounds.

For manuscripts utilizing custom algorithms or software that are central to the research but not yet described in published literature, software must be made available to editors and reviewers. We strongly encourage code deposition in a community repository (e.g. GitHub). See the Nature Portfolio guidelines for submitting code & software for further information.

Data

Policy information about availability of data

All manuscripts must include a data availability statement. This statement should provide the following information, where applicable:

- Accession codes, unique identifiers, or web links for publicly available datasets
- A description of any restrictions on data availability
- For clinical datasets or third party data, please ensure that the statement adheres to our policy

The atomic coordinates and structure factors have been deposited into the Protein Data Bank with accession codes 8IFP [https://doi.org/10.2210/pdb8IFP/pdb] (SARS-CoV-2 3CLpro in complex with compound 1), 8IFQ [https://doi.org/10.2210/pdb8IFQ/pdb] (SARS-CoV-2 3CLpro in complex with compound 2), 8IFR [https://doi.org/10.2210/pdb8IFR/pdb] (SARS-CoV-2 3CLpro in complex with compound 3), 8IFS [https://doi.org/10.2210/pdb8IFS/pdb] (SARS-CoV-2 3CLpro in complex with compound 7), 8IFT [https://doi.org/10.2210/pdb8IFT/pdb] (SARS-CoV-2 3CLpro in complex with compound 10), 8IGX [https://doi.org/10.2210/pdb8IGX/pdb] (SARS-CoV-2 3CLpro in complex with simnotrelvir), and 8IGY [https://doi.org/10.2210/pdb8IGY/pdb] (SARS-CoV-2 3CLpro in complex with nirmatrelvir).
The structure of SARS-CoV-2 3CLpro in complex with boceprevir (PDB code: 6XQU, https://doi.org/10.2210/pdb6XQU/pdb) was obtained from Protein Data Bank.
The cDNA of 3CLpros of SARS-CoV-2 (Gen-Bank: MN908947.3), SARS-CoV (Gen-Bank: AAP13442.1), MERS-CoV (Gen-Bank: MT387202.1), H229E-CoV (Gen-Bank: AF304460.1), HKU1-CoV (Gen-Bank: AY597011.2), NL63-CoV (Gen-Bank: AY567487.2), and OC43-CoV (Gen-Bank: AY903459.1) were obtained from Genbank [https://https.ncbi.nlm.nih.gov/genbank/].
Source data are provided with this paper. All the raw data generated in this study are provided in the Source Data file.

Research involving human participants, their data, or biological material

Policy information about studies with human participants or human data. See also policy information about sex, gender (identity/presentation), and sexual orientation and race, ethnicity and racism.

Reporting on sex and gender	N/A
Reporting on race, ethnicity, or other socially relevant groupings	N/A
Population characteristics	N/A
Recruitment	N/A
Ethics oversight	N/A

Note that full information on the approval of the study protocol must also be provided in the manuscript.

Field-specific reporting

Please select the one below that is the best fit for your research. If you are not sure, read the appropriate sections before making your selection.

- [x] Life sciences
- [ ] Behavioural & social sciences
- [ ] Ecological, evolutionary & environmental sciences

For a reference copy of the document with all sections, see nature.com/documents/nr-reporting-summary-flat.pdf

Life sciences study design

All studies must disclose on these points even when the disclosure is negative.

Sample size	No sample size calculation were performed for the experiments conducted. The sample size for each experiment is provided in the figure legends and in the main manuscript. Sample sizes were chosen to assure statistical differences and reproducibility of the results.<br>The sample sizes for pharmacokenetic studies and rat bone marrow micronucleus test were chosen according to "Technical Guidelines for Non-clinical Pharmacokinetic Studies" issued by CDE.<br>The sample sizes for toxicity studies was chosen according to "Technical Guidelines for Repeated Dose Toxicity Studies" issued by CDE.<br>The preliminary experiment was conducted to determine the proper sample sizes for In vivo efficacy study. The sample sizes for efficacy study were also chosen based on the reported literatures (J. Pharmacol. Pharmacother. 2013. 4(4):303-306, PLoS Biol. 2020. 18(7):e3000410).
Data exclusions	No data were excluded from analysis.
Replication	The experiments were repeated as described in the figure legends and Method section. All attempts at replication were successful.

Randomization	SD rats, cynomolgus monkeys, C57BL/6J mice and K18-hACE2 mice were randomly grouped for animal experiments including in vivo rat bone marrow micronucleus test, repeat dose toxicity studies, animal pharmacokinetics studies, and in vivo efficacy study. Ramdomization is not applied for the other experiments since randomization is not applicable for these in vitro experiments.
Blinding	Blinding was performed in scoring the histopathology of lung and brain. Representive pathology images were selected that represent the mean score after blinded quantification. Blinding was not performed in the determination of crystal structures based on the nature of structural biology. Blinding is not relevant to the other experiments since they are observational studies. All results were analyzed in unbiased ways.

Reporting for specific materials, systems and methods

We require information from authors about some types of materials, experimental systems and methods used in many studies. Here, indicate whether each material, system or method listed is relevant to your study. If you are not sure if a list item applies to your research, read the appropriate section before selecting a response.

Materials & experimental systems

n/a	Involved in the study
☐	☒ Antibodies
☐	☒ Eukaryotic cell lines
☒	☐ Palaeontology and archaeology
☐	☒ Animals and other organisms
☒	☐ Clinical data
☒	☐ Dual use research of concern
☒	☐ Plants

Methods

n/a	Involved in the study
☒	☐ ChIP-seq
☒	☐ Flow cytometry
☒	☐ MRI-based neuroimaging

Antibodies

Antibodies used	Anti-SARS-CoV-2 nucleocapsid protein monoclonal antibody used at a 1:400 dilution in IHC anlysis were purchased from Cell Signaling Technology (#26369).<br>The goat anti-rabbit IgG polymonal antibody (SeraCare, #5220-0336) (1:500) were applied as secondary antibodies in IHC analysis.
Validation	SARS-CoV-2 Nucleocapsid Protein (HL344) Rabbit mAb (CST, #26369) is commercially available, extensively used in prior studies and validated in IHC analysis for K18-hACE2 mice infected with SARS-CoV-2 by manufacturer.<br>The goat anti-rabbit IgG polymonal antibody (SeraCare, #5220-0336) is tested to assure specificity and lot-to-lot consistency using an in-house ELISA assay by manufacturer.

Eukaryotic cell lines

Policy information about cell lines and Sex and Gender in Research

Cell line source(s)	Chinese hamster lung (CHL) and Vero E6 cell line was purchased from ATCC with catalog number CRL-1935 and CRL-1586, respectively. Human PBMC was puchased from Oricells with catalog number FPB004. Human primary hepatocytes were purchased from BioIVT with catalog number F00995-P.
Authentication	The cell lines were not authenticated.
Mycoplasma contamination	Mycoplasma testing confirmed negative.
Commonly misidentified lines (See ICLAC register)	No commonly misidentified cell lines were used.

Animals and other research organisms

Policy information about studies involving animals; ARRIVE guidelines recommended for reporting animal research, and Sex and Gender in Research

Laboratory animals	SD rats (200-260 g, n = 6 for each group) , Male C57BL/6J mice (7-8 weeks old, n = 3 for time point) and cynomolgus monkeys(2-5 kg, 2.5-5 years old, n = 6 for each group) were used in pharmacokinetic (PK) studies. Male C57BL/6J mice (15-30 g, n = 24 for each group) were used in tissue distribution study. SD rats (6-8 weeks old, main group: n = 30 for each group; TK group: n = 8 for each group) and cynomolgus monkeys (2-5 kg, 2.5-5 years old, n = 6-10 for each group) were used in repeat dose toxicity studies. Male SD rats (6-9 weeks old, n = 6 for each group) were used in bone marrow micronucleus test. Male K18-hACE2 mice (7-8 weeks old, n = 2-5 for each group) were used to assess the antiviral activity of the compound.
Wild animals	The study did not involve wild animals.
Reporting on sex	Only male K18-hACE2 mice were used to assess the antiviral activity of the compound, because male mice have been proved to be

	more susceptible to SARS-CoV-2 in comparison to female ones. Only male C57BL/6J mice were used in mouse pharmacokinetic study and tissue distribution study to support the antiviral efficacy of the compound in male K18-hACE2 mice. The micronucleus response is similar between male and female animals in general. Therefore, only male SD rats were used in micronucleus test. For other studies including pharmacokinetic study and toxicology study in SD rats and cynomolgus monkeys, both male and female animals were used.
Field-collected samples	This study did not involve samples collected from the field.
Ethics oversight	The studies were approved by the Institute Animal Care and Use Committee of Shanghai Institute of Materia Medica (SIMM), Wuhan Institute of Virology, Kunming Biomed International (KBI) Ltd, and Simcere. All procedures related to animal handling, care and treatment in efficacy studies were performed according to approved guidelines.

Note that full information on the approval of the study protocol must also be provided in the manuscript.
